# Modulating chromatin accessibility by transactivation and targeting proximal dsgRNAs enhances Cas9 editing efficiency in vivo

**DOI:** 10.1186/s13059-019-1762-8

**Published:** 2019-07-26

**Authors:** Guanwen Liu, Kangquan Yin, Qianwei Zhang, Caixia Gao, Jin-Long Qiu

**Affiliations:** 1 State Key Laboratory of Plant Genomics, Institute of Microbiology, Chinese Academy of Sciences, Beijing, 100101 China; 2 University of Chinese Academy of Sciences, Beijing, 10049 China; 3 State Key Laboratory of Plant Cell and Chromosome Engineering, Institute of Genetics and Developmental Biology, Chinese Academy of Sciences, Beijing, 100101 China

**Keywords:** CRISPR/Cas9, Chromatin accessibility, Transcription activation domain, Proximal dsgRNA

## Abstract

The CRISPR/Cas9 system is unable to edit all targetable genomic sites with full efficiency in vivo. We show that Cas9-mediated editing is more efficient in open chromatin regions than in closed chromatin regions in rice. A construct (Cas9-TV) formed by fusing a synthetic transcription activation domain to Cas9 edits target sites more efficiently, even in closed chromatin regions. Moreover, combining Cas9-TV with a proximally binding dead sgRNA (dsgRNA) further improves editing efficiency up to several folds. The use of Cas9-TV/dsgRNA thus provides a novel strategy for obtaining efficient genome editing in vivo, especially at nuclease-refractory target sites.

**Electronic supplementary material:**

The online version of this article (10.1186/s13059-019-1762-8) contains supplementary material, which is available to authorized users.

## Background

The CRISPR/Cas9 system has been widely and successfully used for genome engineering in diverse eukaryotic species and is revolutionizing biology and providing powerful tools for gene therapy and plant breeding [1]. However, the efficiency of editing at different genomic loci varies greatly, both in animal and plant cells [2, 3]. The low CRISPR/Cas9 editing efficiency at some sites limits the availability of in vivo targets and thereby constrains further applications [4].

Unlike the prokaryotic DNA that Cas9 evolved to target, eukaryotic genomic DNA is wrapped around histones and further compacted to form higher-order chromatin structures [5] that may hinder the binding of Cas9 to its targets. Genome-wide mapping of the binding sites of catalytically inactive Cas9 (dCas9) in mammalian cells revealed that they were enriched in open chromatin regions [6, 7]. Moreover, the production of CRISPR/Cas9-induced insertions and deletions (indels) in human cells was higher at sites in open chromatin regions [8]. In vitro and in vivo experiments have demonstrated that Cas9 binding and cleavage are inhibited by nucleosomes, the basic units of chromatin [9–12]. In agreement with this, Cas9-mediated genome editing was more efficient in euchromatic than in heterochromatic regions in HEK293T, HeLa, and human fibroblast cells [4]. Interestingly, chromatin structure had an even more pronounced inhibitory effect on the off-target activity of CRISPR/Cas9 [13]. In contrast, chromatin accessibility was not found to affect CRISPR/Cas9 activity in zebrafish [14]. Whether chromatin accessibility affects Cas9 editing in plant cells is not yet known.

There have been some attempts to alter local accessibility to improve Cas9 activity in vivo. The proxy-CRISPR strategy uses an additional catalytically dead SpCas9 (dCas9) to bind at proximal locations. This rendered target sites accessible to FnCas9, CjCas9, NcCas9, and FnCpf1 and thus improved the editing efficiency [15]. However, this method relies on the genome being accessible to SpCas9 and requires co-expression of two different CRISPR/Cas systems, which inevitably increases vector size and the difficulty of in vivo application. Recently, a method called CRISPR-chrom, in which Cas9 orthologs are fused with chromatin-modulating peptides (CMPs), substantially improved Cas9 editing efficiency, particularly at refractory sites [16]. CMPs are truncated forms of endogenous proteins, and it is not yet known whether their overexpression can have dominant negative effects. Probably a variety of approaches are needed to gain full access to eukaryotic DNA for editing [17].

Here, we describe several lines of evidence indicating that Cas9 genome editing efficiency is correlated with chromatin accessibility in rice cells. We fused a synthetic transcription activation domain to Cas9, generating the construct Cas9-TV. This fusion significantly improved Cas9-mediated genome editing efficiency in both open and closed chromatin regions. Furthermore, when Cas9-TV was combined with proximally targeting dead sgRNAs (dsgRNAs), even higher genome editing efficiencies were obtained.

## Results

### Cas9 genome editing is more efficient in open chromatin regions of rice

**Fig. 1:**
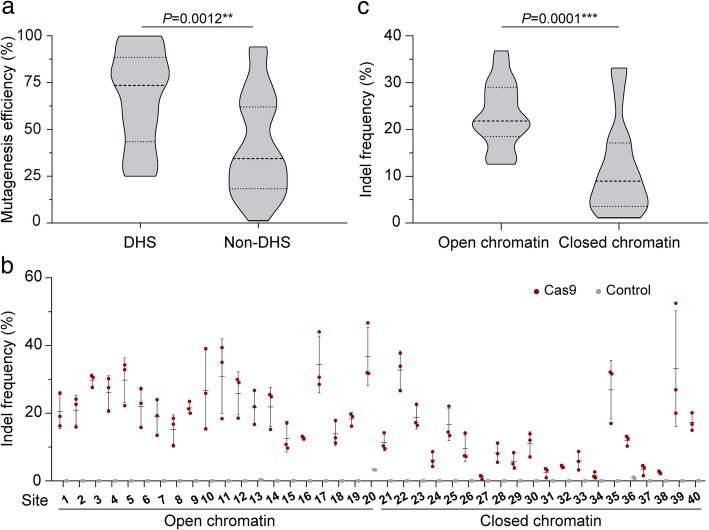
Effect of chromatin accessibility on Cas9 genome editing efficiency in rice. **a** Summary of CRISPR/Cas9-mediated mutation numbers and chromatin accessibility at 70 target sites. Mutagenesis efficiency was measured on regenerated T0 rice plants by PCR/RE. The accessibility of each target site was obtained from the high-resolution maps of DNase I hypersensitive sites (DHS) of rice generated by Zhang et al. [19]. **b** Indel frequencies at 40 target sites in 20 rice genes examined in protoplasts. Two sites were targeted in each gene by independent sgRNAs. Indel frequencies were measured by sequencing targeted amplicons. Data are from sets of three independent biological replicates (*n* = 3) and are shown as means ± s.e.m. **c** Summary of indel frequencies and chromatin states at the 40 target sites in **b**. In **a** and **c**, the distribution of indel frequencies is shown on a violin plot. The middle dashed line represents the mean. The outer dashed lines represent the interquartile ranges. *P* values were calculated by two-tailed Mann-Whitney tests. **P < 0.01, ***P < 0.001

We used the CRISPR/Cas9 system to edit 41 rice genes with 70 sgRNAs (Additional file 1: Table S1). Cas9 and the various sgRNAs were transformed into rice callus via *Agrobacterium* transformation. Editing in regenerated T0 plants was analyzed by PCR/RE and confirmed by Sanger sequencing. The insertion/deletion (indel) frequencies induced by CRISPR/Cas9 at various target sites were quite variable (Additional file 1: Table S1). We then analyzed whether the indel frequencies were associated with chromatin accessibility. Open chromatin is DNase I hypersensitive (DH) [18], and comprehensive DNase I hypersensitivity data for the rice genome [19] are available. Using these data, we found that the frequencies of Cas9-induced indels at the target sites we tested were significantly higher at DH sites (Fig. 1a), suggesting that CRISPR/Cas9 activity in rice is affected by chromatin openness. To confirm that chromatin structure influences Cas9 editing in rice, we tested another 20 genes in open and closed chromatin regions based on the rice open chromatin map [19]. Two sgRNAs were designed for each gene, one targeting the promoter and the other an exon (Additional file 1: Table S2). Cas9 and each of these sgRNAs were transformed into rice protoplasts, and the indel frequencies at all 40 target sites were measured by targeted deep sequencing (Fig. 1b). The result confirmed that editing efficiency was higher in open than in closed chromatin regions (Fig. 1c).

**Fig. 2:**
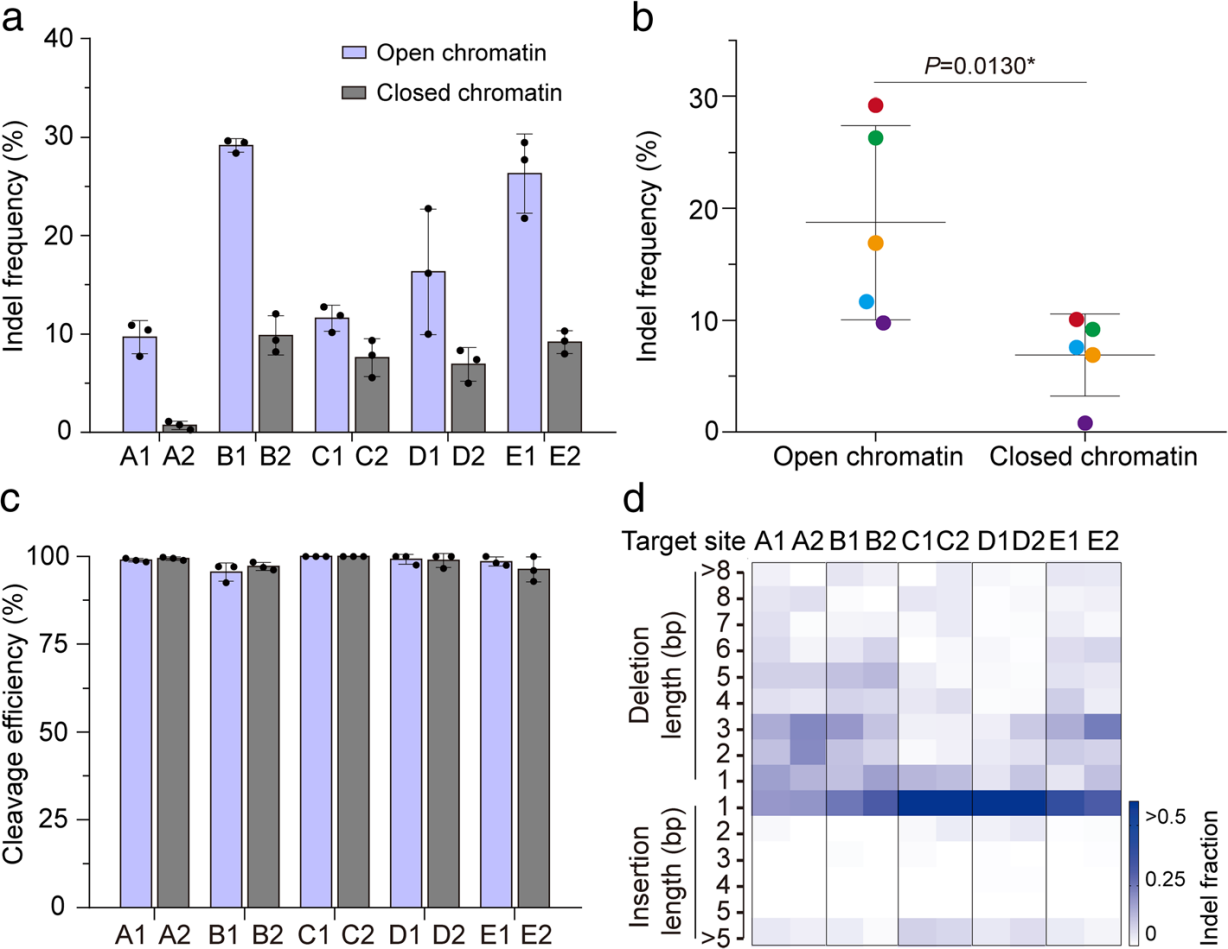
Cas9 editing in rice is more efficient in open chromatin regions than in closed chromatin regions. **a** Pairwise comparisons of indel frequencies at pairs of sgRNA-targeted sites in open and closed chromatin regions, respectively. Indel frequencies were measured in rice protoplasts by sequencing targeted amplicons. Data are from sets of three independent biological replicates (*n* = 3) and are shown as means ± s.e.m. **b** Summary of Cas9 editing efficiencies in **a**. Dots of different color represent different target sites. *P* values were calculated by two-tailed Mann-Whitney tests. *P < 0.05. **c** Cas9 cleaves all 10 target sites equally well in the chromatin-free state. PCR products containing corresponding target sites were incubated with Cas9 ribonucleoprotein (RNP) complexes and visualized and measured on agarose gels. The data are from three independent biological replicates (*n* = 3) and shown as means ± s.e.m. **d** Indel patterns generated at the 10 target sites. All experiments were repeated three times with similar results

To exclude the possible impact of sequence composition of spacers on editing efficiency, we identified five independent spacers (sgRNA A~E) with sequences in both open and closed chromatin regions (Additional file 1: Table S3). Pairwise comparisons of indel frequencies at these sites revealed that Cas9 activity was higher in open chromatin regions than in closed chromatin regions by factors of up to 13.4-fold, while the indel frequencies induced by different sgRNAs were highly variable (Fig. 2a, b). Interestingly, Cas9 is able to cleave all these target sites almost equally well when PCR products or chromatin-free DNA is targeted in vitro (Fig. 2c and Additional file 1: Figure S1). Moreover, the indel patterns generated at the pairs of target sites were similar (Fig. 2d). Together, these results show that CRISPR/Cas9 genome editing in rice cells is more efficient in open chromatin regions than in closed ones.

### Fusing a synthetic transcription activator to Cas9 increases its editing activity in rice cells

**Fig. 3:**
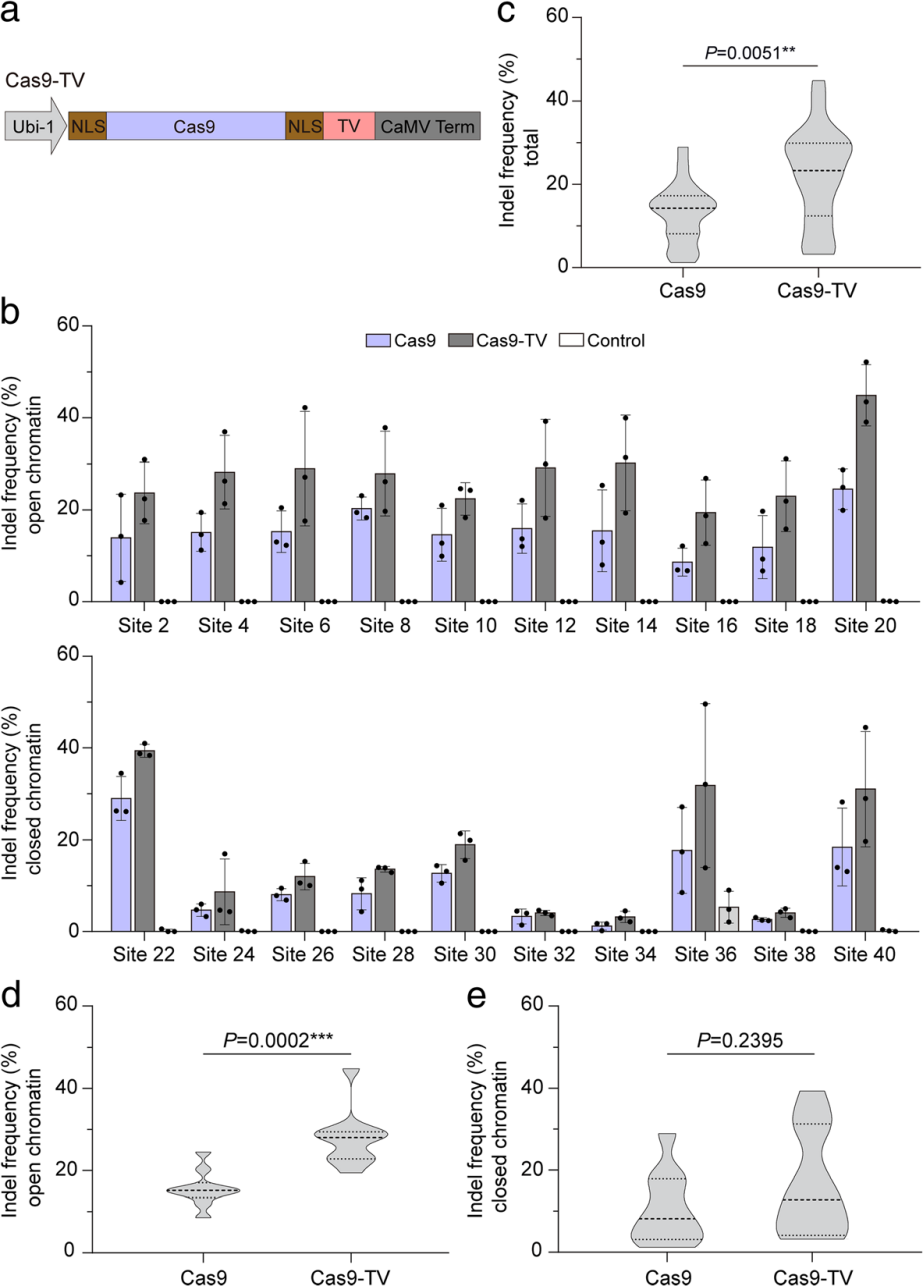
Fusion of a synthetic transcription activator to Cas9 improves its editing efficiency. **a** Diagrams of the Cas9-TV construct. **b** Indel frequencies induced by Cas9 and Cas9-TV at the 20 target sites in rice protoplasts. An untreated protoplast sample was used as a control. Data are from sets of three independent biological replicates (*n* = 3) and are shown as means ± s.e.m. **c** Summary of the indel frequencies induced by Cas9 and Cas9-TV at the 20 target sites. **d** Summary of indel frequencies induced by Cas9 and Cas9-TV at target sites in open chromatin regions. **e** Summary of indel frequencies induced by Cas9 and Cas9-TV at target sites in closed chromatin regions. In **c**, **d**, and **e**, the distribution of indel frequencies is shown on a violin plot. The middle dashed line represents the mean. The outer dashed lines represent the interquartile ranges. *P* values were calculated from two-tailed Mann-Whitney tests. *P < 0.05, ***P < 0.001

Highly expressed genes are frequently associated with open chromatin [20, 21]. VP16, a transcription activation domain, can induce remodeling and unfolding of condensed chromatin [22]. Furthermore, dCas9-VP64 (with four copies of VP16) can activate expression of target genes even in closed chromatin regions [23]. This led us to suppose that fusion of Cas9 with a transcription activation domain might improve Cas9 editing efficiency in the context of chromatin. To test this hypothesis, a synthetic transcription activation domain (thereafter called TV) [24], which contains six copies of the TAD (transcription activation domain) of the TALE (transcription activator-like effector) protein AvrBs3 from *Xanthomonas campestris* and eight copies of VP16, was fused to the C-terminus of Cas9 to generate Cas9-TV (Fig. 3a). The genome editing efficiency of Cas9-TV was investigated in rice protoplasts with the 20 sgRNAs that target different chromatin regions (Additional file 1: Table S2). The indel frequencies induced by Cas9 and Cas9-TV at the target sites ranged from 1.95 to 29.56% and 3.81 to 44.85%, respectively (Fig. 3b), and the genome editing efficiency of Cas9-TV was higher than that of Cas9 at all sites tested (Fig. 3c). On average, Cas9-TV-induced indel frequency was 1.87-fold and 1.44-fold higher than Cas9 in open and closed chromatin, respectively (Fig. 3d, e). We also analyzed the indel patterns generated by Cas9-TV and Cas9 and found that the patterns were similar (Additional file 1: Figure S2). These data suggest that Cas9-TV in vivo editing activity is increased at target sites in both open and closed chromatin regions.

### Proximal targeting with dsgRNA improves genome editing by Cas9-TV and Cas9

**Fig. 4:**
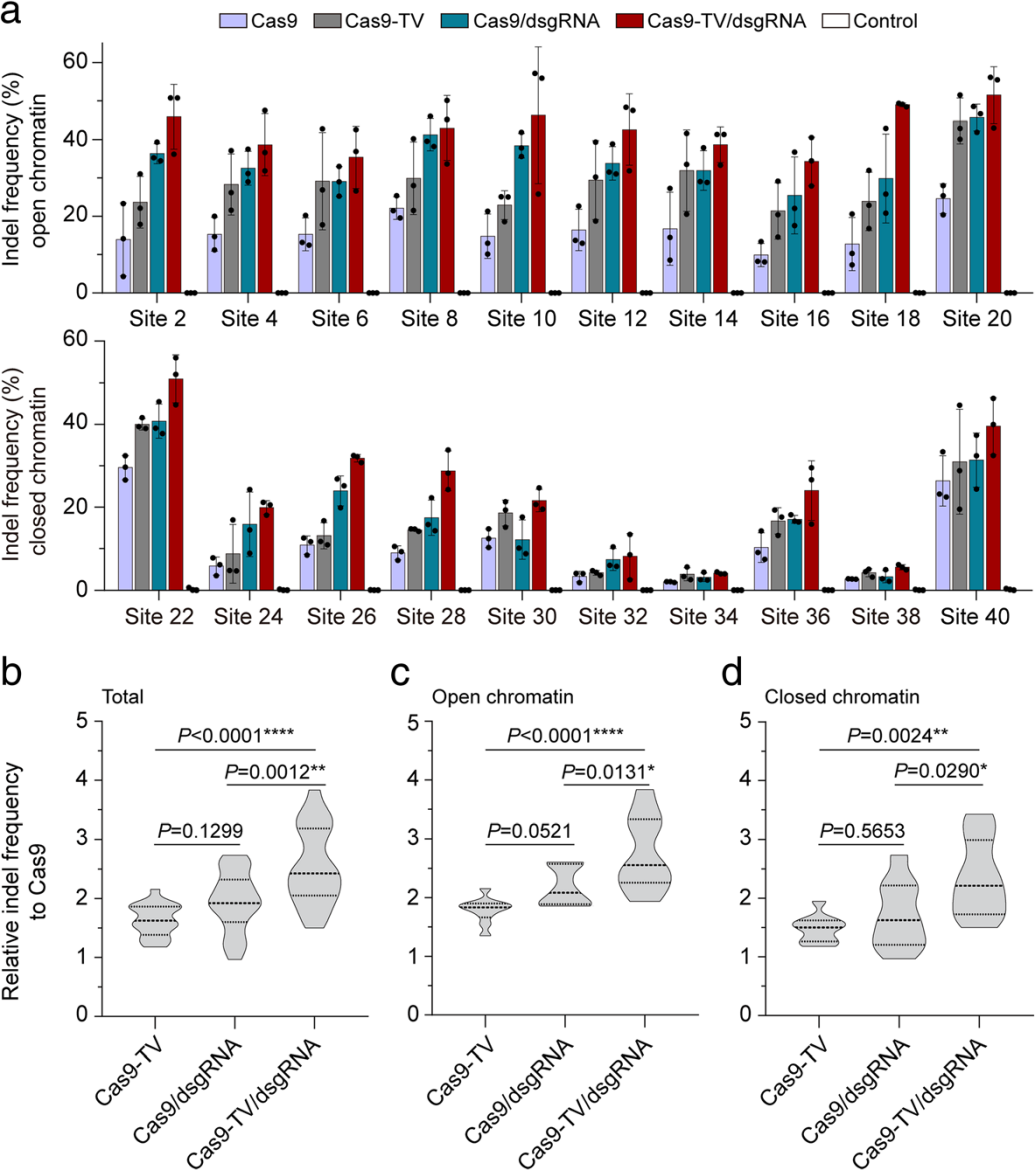
Proximal targeting by dsgRNA enhances Cas9-TV editing. **a** Indel frequencies induced by Cas9, Cas9-TV, Cas9/dsgRNA, and Cas9-TV/dsgRNA at 20 target sites in rice protoplasts. An untreated protoplast sample was used as a control. Indel frequencies were measured by sequencing targeted amplicons. Data are from sets of three independent biological replicates (*n* = 3) and are shown as means ± s.e.m. **b** Fold changes of indel frequencies induced by Cas9-TV, Cas9/dsgRNA, and Cas9-TV/dsgRNA relative to Cas9 at the 20 target sites. **c** Summary of the fold changes of indel frequencies at sites in open chromatin regions. **d** Summary of the fold changes of indel frequencies at target sites in closed chromatin regions. In **b**, **c** and **d**, the distribution of indel frequencies is shown on a violin plot. The middle dashed line represents the mean. The outer dashed lines represent the interquartile ranges. *P* values were calculated by one-way ANOVA. *P < 0.05, **P < 0.01, ****P < 0.0001

Next, we set out to further improve Cas9-TV genome editing efficiency, especially for target sites in closed chromatin regions. Dead sgRNAs (dsgRNAs) with 14- or 15-base-pair (bp) spacer sequences can guide Cas9 to target sites without inducing double-strand breaks (DSBs) [25–27]. Hence, proximal dsgRNAs might increase the accessibility of Cas9-TV to target genomic loci (Additional file 1: Figure S3), similar to that of proximal CRISPR targeting [15]. To test this idea, we used the 20 sgRNAs targeting sites in the rice genome (Additional file 1: Table S2) and designed dsgRNAs targeting proximal sites near each one (Additional file 1: Table S4). The distances between sgRNA targeting sites and dsgRNA binding sites ranged from 32 to 92 bp. When each of the combinations of dsgRNA with sgRNA or sgRNA alone was transformed along with Cas9-TV or Cas9 into rice protoplasts, the proximal dsgRNA improved the efficiency of Cas9-TV editing at all the target sites (Fig. 4a). On average, the indel frequency obtained with Cas9-TV combined with a proximal dsgRNA was 1.5-fold higher than that with Cas9-TV, and 2.5-fold higher than that with Cas9 (Fig. 4b). As expected, we detected no indels at the dsgRNA targeting sites (Additional file 1: Figure S4). The proximal dsgRNAs stimulated Cas9-TV editing in both open and closed chromatin regions (Fig. 4c, d) and did not affect the patterns of indels induced by Cas9-TV (Additional file 1: Figure S5). Moreover, we found that proximally targeting dsgRNAs also increased Cas9 activity at all target sites tested (Fig. 4a). These results suggest that proximal dsgRNA targeting improves Cas9-TV and Cas9 genome editing activities in vivo, in both open and closed chromatin regions.

**Fig. 5:**
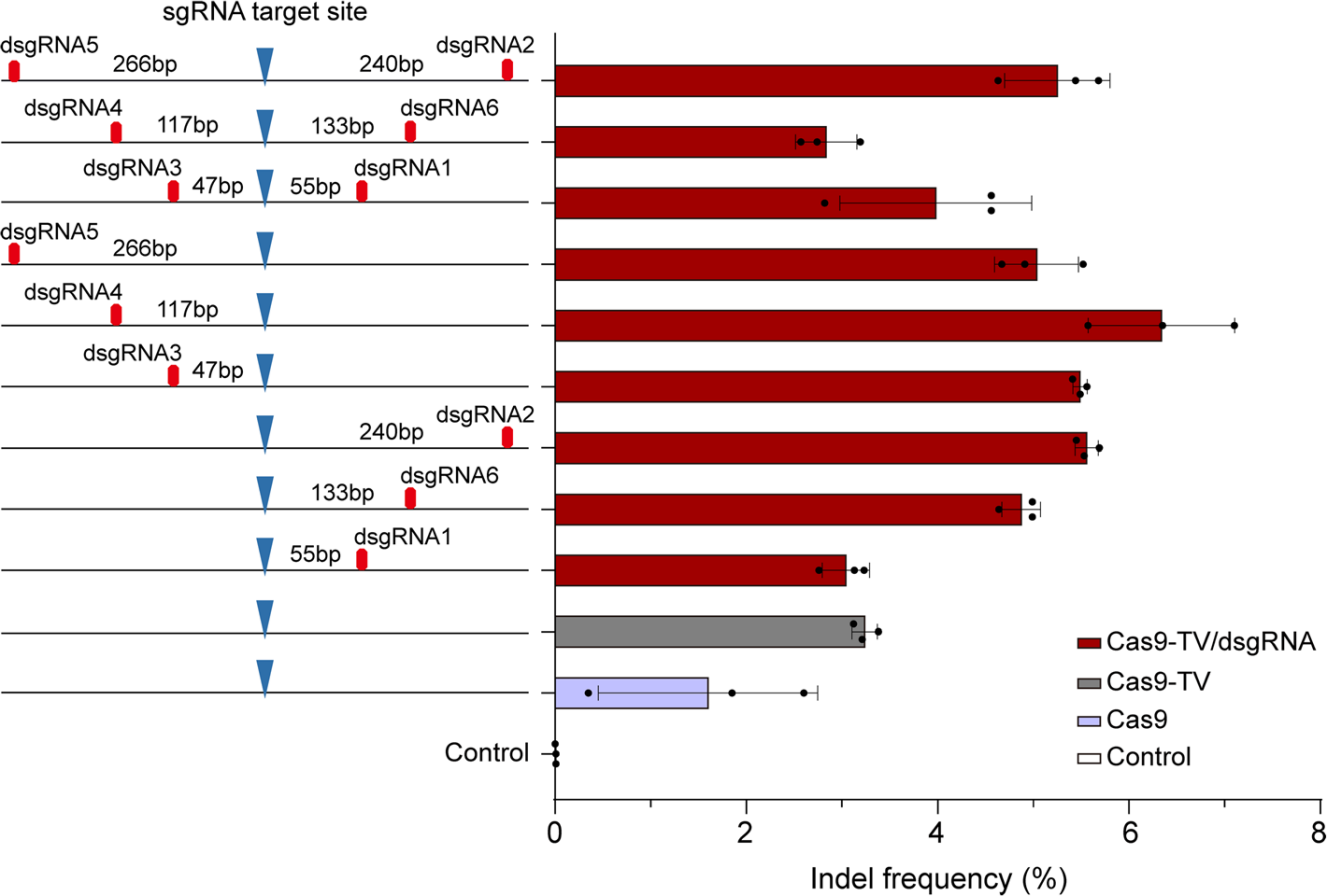
Effects of the position of proximal dsgRNA on Cas9-TV editing. The distances were calculated based on the nucleotides that separate the sgRNA and dsgRNA target sites. Indel frequencies were measured by sequencing targeted amplicons in rice protoplasts. An untreated protoplast sample was used as a control. Data are from sets of three independent biological replicates (*n* = 3) and are shown as means ± s.e.m

To optimize the proximal dsgRNA targeting, we designed dsgRNAs 1, 2, 6 and dsgRNAs 3, 4, 5 targeting sites on either side of the PAM sequence of sgRNA34 (Additional file 1: Table S5 and Figure S6). The distances that separate dsgRNA and sgRNA binding sites ranged from 47 to 266 bp (Fig. 5). Each dsgRNA or dsgRNA pair was co-transformed with Cas9-TV and the corresponding sgRNA into rice protoplasts, and indel frequencies were measured by targeted deep sequencing. While all the dsgRNAs enhanced editing, dsgRNA4 targeting a site 117 bp from the cleavage site had the greatest effect (Fig. 5). We also found that the position of dsgRNAs relative to the PAM (downstream vs upstream) did not significantly affect editing efficiency (Fig. 5). In addition, using pairs of dsgRNA rather than single dsgRNA did not further increase Cas9-TV-mediated editing and sometimes even compromised the effect of the single dsgRNA (Fig. 5). Similar results were obtained for Cas9-mediated editing (Additional file 1: Figure S7).

### Cas9-TV together with proximal dsgRNA increases chromatin accessibility

**Fig. 6:**
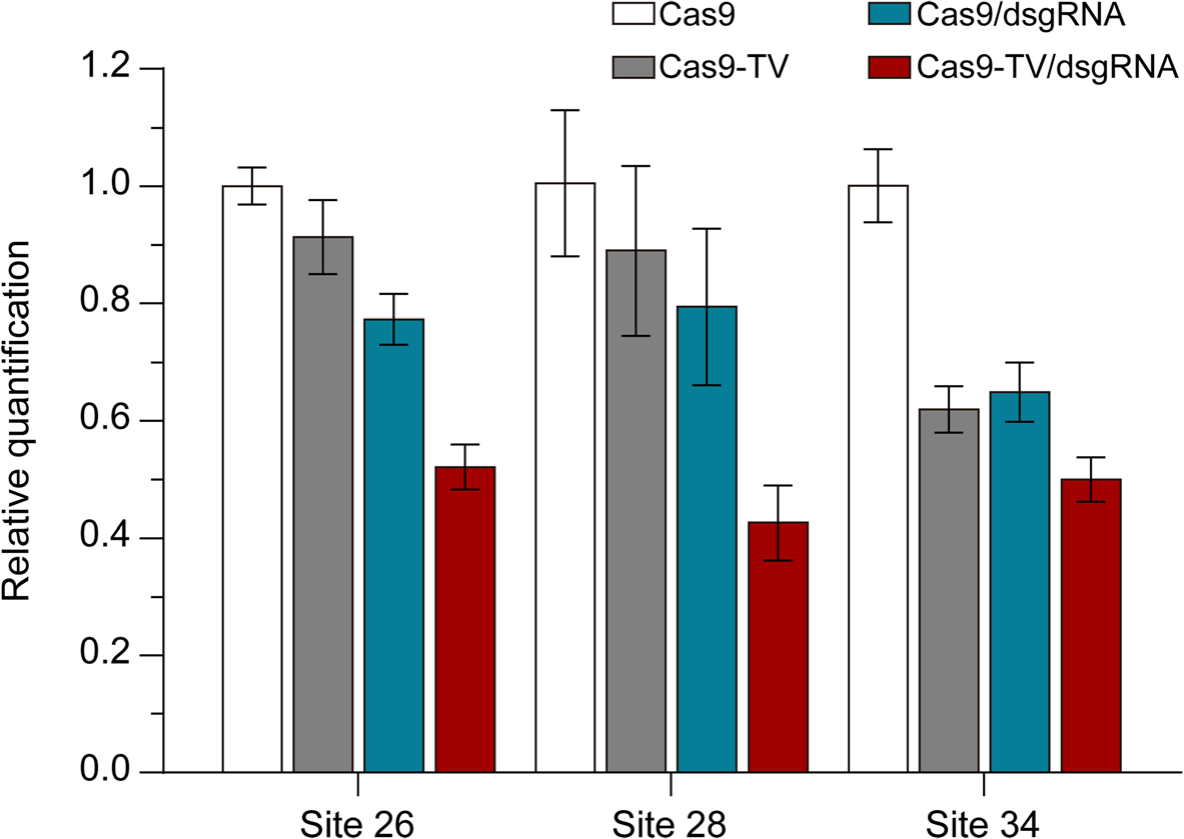
Cas9-TV and proximal dsgRNA alter local chromatin accessibility. Rice protoplasts were transfected with Cas9, Cas9-TV, Cas9/dsgRNA, and Cas9-TV/dsgRNA, respectively, and local chromatin accessibility around target sites was analyzed by low-input DNase I assays. The fractions of intact genomic DNA were quantified by real-time PCR. For each site, relative amount of intact genomic DNA in the Cas9-treated sample was set as one unit. Error bars indicate SD of three replicates

It seemed conceivable that the enhanced editing efficiency of Cas9-TV was due to an increased NHEJ mutagenesis rate as expression of the target gene is induced by the Cas9-TV. Although this was unlikely, since the target sites we chose are located in coding regions, we checked the expression levels of four target genes. As expected, Cas9-TV did not induce significant changes in the expression of these genes, whereas Cas9-TV targeting the promoter of *OsYUCCA4* increased its expression (Additional file 1: Figure S8). To see whether binding of Ca9-TV and dsgRNA altered the chromatin structure in the targeted regions, we measured chromatin accessibility at sites 26, 28, and 34 using DNase I digestion assays. We found that both Cas9-TV and dsgRNA targeting increased chromatin accessibility at the targeted sites, and the combination had an even greater effect at each of the sites (Fig. 6). These results indicate that Cas9-TV, proximal dsgRNA, and Cas9-TV/dsgRNA are able to increase chromatin accessibility at in vivo target sites, and their effectiveness in this respect seems to be correlated with the efficiency with which they perform genome editing.

### Neither the TV nor proximal dsgRNA increases the off-target activity of Cas9

**Fig. 7:**
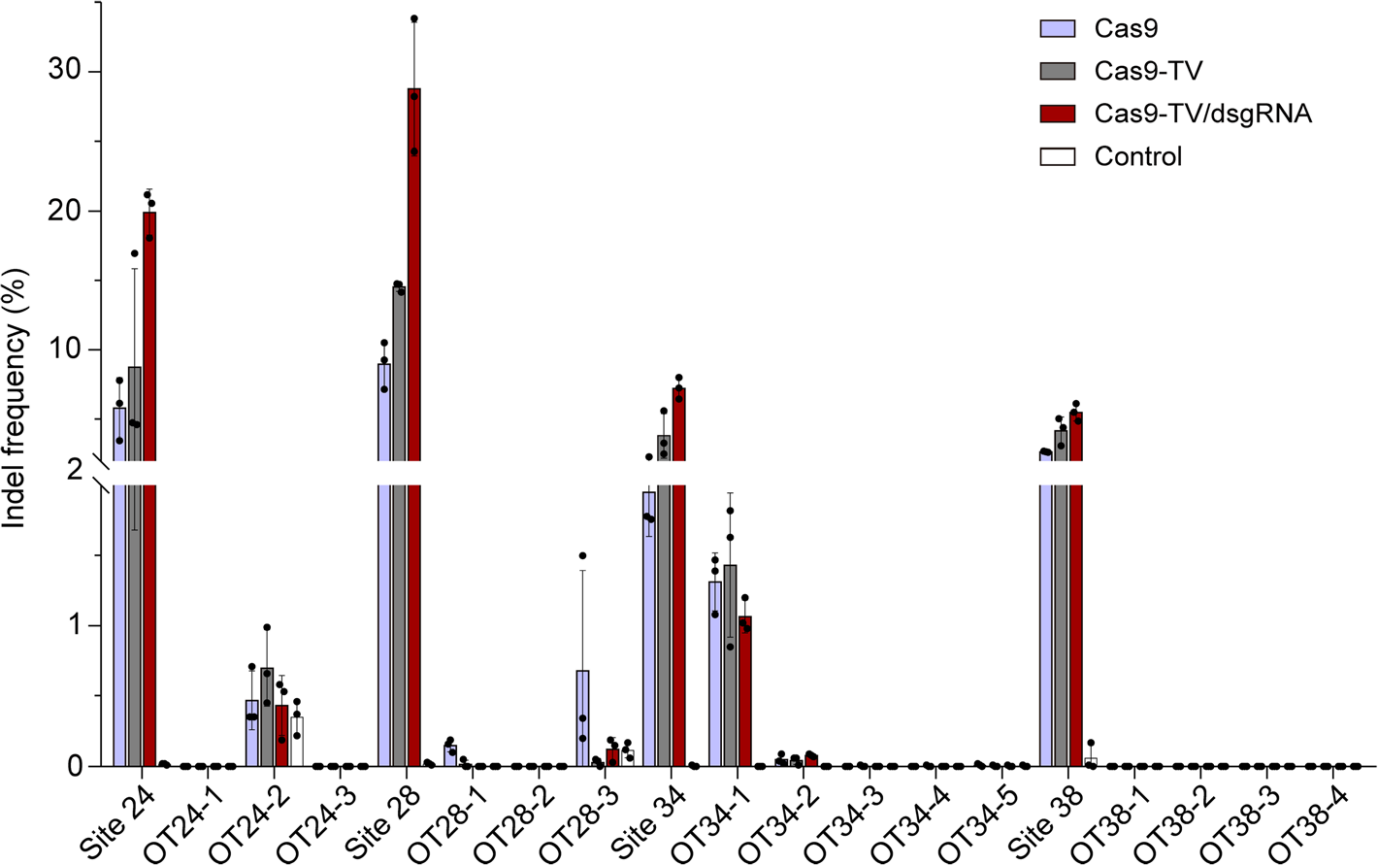
Comparison of off-target activities of Cas9, Cas9-TV, and Cas9-TV/dsgRNA. Indel frequencies were measured by sequencing targeted amplicons in rice protoplasts. An untreated protoplast sample was used as a control. Data are from sets of three independent biological replicates (*n* = 3) and are shown as means ± s.e.m

Finally, we examined the off-target effects of Cas9-TV and Cas9-TV/dsgRNA by measuring indel frequencies by sequencing targeted amplicons at target and off-target sites using sgRNAs 24, 28, 34, and 38. In this analysis, using the online tool, CRISPR-P [28], three potential off-target (OT) sites with 2 to 4 mismatches were identified for sgRNAs 24 and 28, respectively, as well as four off-target sites for sgRNA38 and five for sgRNA34 (Additional file 1: Table S6). Cas9-TV again had higher on-target activity than Cas9 at all the target sites (Fig. 7). On the other hand, even though Cas9, Cas9-TV, and Cas9-TV/dsgRNA induced indels at the OT24-2 site for sgRNA24 and OT34-1 site for sgRNA34, their frequencies were similar, and none of the nucleases induced significant numbers of indels at the OT24-1 and OT24-3 sites for sgRNA24; at the OT28-1 and OT28-2 sites for sgRNA28; at the OT34-2, OT34-3, OT34-4, and OT34-5 sites for sgRNA34; and at the off-target sites for sgRNA38. Surprisingly, indel frequencies induced by Cas9-TV and Cas9-TV/dsgRNA were lower than those by Cas9 at the OT28-3 site (Fig. 7). These results indicate that fusion of TV and proximal dsgRNAs do not alter the off-target activity of Cas9.

## Discussion

In this study, we demonstrate a correlation between chromatin openness and Cas9 editing efficiency in rice, an important crop worldwide. Since the spacer sequence also contributes to Cas9-mediated editing efficiency [8], we used sgRNAs that targeted sites in distinct chromatin states. This strategy excluded the effect of spacer sequence bias on genome editing efficiency and confirmed that Cas9-mediated genome editing efficiency was higher in open chromatin regions. There is growing focus on the effect of chromatin states on genome editing efficiency in different eukaryotic cells [6–8, 14]. However, methods to manipulate chromatin accessibility to enhance genome editing in vivo remain limited. Here, we have developed Cas9-TV, a hybrid protein generated by fusing a synthetic transcription domain (TV) to Cas9, as a strategy to modulate chromatin structure and enhance editing efficiency. Moreover, targeting with dsgRNAs around 120 bp proximal to the cleavage site further substantially increased editing efficiency at all sites tested. We conclude that Cas9-TV together with proximal dsgRNA targeting provides a robust method for enhancing genome editing efficiency in rice.

Interestingly, both Cas9-TV and Cas9-TV/dsgRNA enhance editing equally in open and closed chromatin regions (Fig. 4c, d). This may be a result of the dynamic nature of chromatin structure: nucleosomes themselves are highly dynamic and often undergo “site exposure” conformational fluctuations that transiently allow or prevent access of DNA binding proteins [29–31]. Genome-wide ATAC-seq profiling has also revealed dynamic changes in chromatin state from open to closed and closed to open during the production of human induced pluripotent stem (iPS) cells [32]. In the present study, when targets were in a fully open (chromatin-free) configuration, Cas9 cleaved them almost completely (Fig. 2c). However, the in vivo editing efficiencies at these target sites were quite variable (Figs. 3 and 4), even though Cas9-TV/dsgRNA switched chromatin structure to a more open state (Fig. 6). This suggests that chromatin openness is relative and that there are temporal windows for nuclease (Cas9 or Cas9-TV) binding in both open and closed chromatin. When Cas9-TV binds to a target in an “open” temporal window, it promotes a more open chromatin configuration in the vicinity. Chromatin structure actually involves an alternation between open and closed states [33], and making use of factors that effect this alternation may further improve access of Cas9 to target DNA. For example, trichostatin A (TSA), which modulates chromatin structure by inhibiting histone deacetylase, has been successfully used in the induction of iPS cells [34], and it should be worthwhile to examine its effect on genome editing in various other types of cell.

Our method has some advantages over others as a method for stimulating editing. For example, the proxy-CRISPR method uses two CRISPR/Cas systems, which inevitably increases vector size and reduces protoplast transformation efficiency. In the CRISPR-chrom method, the chromosome-modulating peptides originate from mammalian cells and may not work in plants. However, since the TV domain is effective as a transcriptional activator in yeast, plants, and animals, and dCas9-TV is also effective in human embryonic kidney (HEK) 293T cells [24], we speculate that combinations of Cas9-TV and Cas9-TV with dsgRNA will work in many types of eukaryotic cell. Moreover, while these constructs enhance genome editing efficiency, they do not increase off-target effects. The fact that our sgRNA-dsgRNA strategy improves genome editing in plants also suggests that two or more sgRNAs binding at nearby sites of the same target gene may have synergist effects, so that it might not be necessary to use the sgRNA-dsgRNA strategy when employing more than one sgRNA to target the same gene.

Other CRISPR/Cas systems are being discovered and engineered for genome editing in eukaryotes. However, some of them have low activity in vivo [15]. Since fusing TV to Cas9 can improve editing efficiency, this strategy may also work for other CRISPR/Cas systems, such as SaCas9 or Cas12a (formerly Cpf1), in plants. Nonetheless, our method also has some limitations. For example, binding of Cas9-TV may activate genes around the sgRNA and dsgRNA target sites. This may be particularly important when using Cas9-TV/dsgRNA to generate gene-edited plants, since these might have phenotypes caused by Cas9-TV/dsgRNA-mediated gene activation rather than editing. However, this side effect can be eliminated by transient expression of the editing components or isolating transgene-free plants. Additionally, there are most probably factors other than chromatin structures that affect genome editing efficiency in vivo, and not all closed target sites are efficiently edited by Cas9-TV/dsgRNA. Together, these considerations imply that efforts should continue to produce more powerful and universal genome editing tools.

## Conclusion

In this study, we describe a new strategy for improving CRISPR/Cas9-mediated genome editing efficiency in rice by fusing a synthetic transcription activation domain (TV) to Cas9. This fusion protein, Cas9-TV, increased editing efficiency at all the target sites tested. Moreover, adding a proximal dsgRNA further improved the editing efficiency by up to fourfold. Importantly, Cas9-TV and Cas9-TV/dsgRNA stimulated editing at sites in both open and closed chromatin regions. In addition, the off-target effects of Cas9-TV were similar to those of Cas9.

## Methods

### Plasmid construction

The VP64 and 2TAL (two copies of the TAD of the TALE protein AvrBs3 from *Xanthomonas campestris*) coding sequences were codon-optimized for rice (*Oryza sativa*) and synthesized commercially (GenScript, Nanjing, China). The VP64 coding sequence was fused to the 3′ end of Cas9 by overlapping PCR, and an *Avr* II site was introduced between Cas9 and VP64. The Cas9-VP64 fusion gene was cloned into pJIT163 to generate p163-Cas9-VP64. Then, one copy of VP64 and three copies of the 2TAL fragment were inserted into the *Avr* II site of p163-Cas9-VP64 to generate p163-Cas9-TV. Different sgRNAs were introduced into pOsU3-sgRNA as previously described [35]. The sgRNA-dsgRNA co-expression plasmid was constructed as previously reported [36]. All the primers used in this work are listed in Additional file 1: Table S7 and were synthesized by GenScript.

### DNase-seq data analysis

DNase-seq data for rice seedlings (GSE26610) were reported previously [19] and obtained from the NCBI Gene Expression Omnibus (GEO). The DNase-seq data were loaded into Gbrowse of the rice annotation project database (RAP-DB), and the chromatin states of target sites were viewed.

### Protoplast transfection

Two-week-old seedlings of rice cultivar “Nipponbare” were used for isolating protoplasts. Protoplast isolation and transfection were performed following the standard protocol [35]. Plasmids (10 μg each construct) were transfected into protoplasts via PEG-mediated transfection.

### *Agrobacterium-*mediated rice transformation

*Agrobacterium tumefaciens* strain AGL1 was transformed with binary vectors harboring Cas9 and sgRNA expression cassettes by electroporation. *Agrobacterium-*mediated transformation of embryogenic callus cells of rice cultivar Nipponbare was conducted according to Hiei et al. [37]. Transgenic seedlings were selected on hygromycin-containing (50 μg/mL) medium.

### Extraction of plant genomic DNA

The transfected protoplasts were incubated at 28 °C. After 48 h, the protoplasts were collected and genomic DNA was extracted by the CTAB method [38].

### PCR amplification of targeted regions and next-generation sequencing

Genomic DNA extracted from protoplasts was used as a template in PCR. In the first-round PCR, specific primers were used to amplify the genomic regions flanking the CRISPR target sites (Additional file 1: Table S7). In the second round, 150–250-bp PCR products were amplified using primers (Additional file 1: Table S7) to introduce forward and reverse barcodes into the first-round PCR products. Equal amounts of the final PCR products were pooled and sequenced commercially (GENEWIZ, Suzhou, China) by paired-end read sequencing using the Illumina NextSeq 500 platform. The sgRNA target sites were examined for indels. Sequencing of each amplicon was repeated three times, using genomic DNA from three independent protoplast samples.

### In vitro cleavage by Cas9 RNP

In vitro cleavage by Cas9 RNP was performed as previously reported [39]. Target DNA sequences were amplified by PCR using specific primers (Additional file 1: Table S7), then purified and eluted in RNase-free water. Cas9 protein (1 μg) and sgRNA (1 μg) were pre-mixed and incubated with the target DNA (200 ng) at 37 °C for 1 h. The products were then separated on 2% agarose gel, and band intensities were measured using ImageJ to calculate Cas9 cleavage activity.

### Real-time PCR analysis

Transfected protoplasts were incubated at 28 °C for 24 h. Samples of 2 × 10^6^ transfected rice protoplasts were used for RNA extraction. Total RNA was extracted with TRNzol reagent (Tiangen Biotech), then reverse transcribed into first-strand cDNA using a Maxima H Minus First Strand cDNA Synthesis Kit with dsDNase (Thermo Fisher Scientific). Real-time PCR analysis was performed using SYBR Premix Ex Taq II (TaKaRa Bio) and run on a Bio-Rad CFX96 PCR System (Bio-Rad Laboratories). Normalized expression levels were calculated with CFX Manager Software (Bio-Rad) using the 2^*-ΔΔ(t)*^ method. The *Ubiquitin* (*Ubi*) was used as the internal reference gene. The primers used are listed in Additional file 1: Table S7.

### Detection of chromatin accessibility

Low-input DNase I digestion assays were performed as previously reported [40]. Transfected protoplasts were incubated at 28 °C for 24 h. Samples of 4 × 10^5^ transfected rice protoplasts were resuspended in 45 μL lysis buffer (10 mM Tris-HCl [pH 7.5], 10 mM NaCl, 3 mM MgCl_2_, 0.1% Triton X-100) and incubated on ice for 5 min, then DNase I (1000 U/ml, Sigma, AMPD1-1KT) was added to a final concentration of 2 U/mL. The samples were incubated at 37 °C for another 5 min before the reaction was terminated by adding 50 μL stop buffer (10 mM Tris-HCl [pH 7.5], 10 mM NaCl, 0.15% SDS, 10 mM EDTA) containing 1 U Proteinase K and incubating at 55 °C for 1 h. Genomic DNA was extracted from each sample by the phenol-chloroform method [41] and analyzed by real-time qPCR (SYBR Premix Ex TaqTM II, Takara). The primers used are listed in Additional file 1: Table S7 and were synthesized by GenScript.

### Detection of off-targets

Potential off-target sites for sgRNAs 24, 28, 34, and 38 were predicted by the online tool CRISPR-P [28]. Locus-specific primers for these sites were designed to produce PCR products of ~ 150 to 250 bp. In the first round of PCR, specific primers were used to amplify the genomic regions flanking the on-target and off-target sites. The resulting PCR products were used as templates for second-round PCR, with barcodes added to each end of the PCR products using the primers listed in Additional file 1: Table S7. The PCR products were then pooled in equal quantities for next-generation sequencing. On-target and potential off-target sites were examined for indels. Sequencing of each amplicon was repeated three times, using genomic DNA from three independent protoplast samples.

## Additional files

Additional file 1: **Figure S1.** Agarose gels of the PCR products containing the 10 target sites shown in Fig. 2 after incubation with Cas9 ribonucleoprotein (RNP) complexes. **Figure S2.** Indel patterns generated at the target sites by Cas9 and Cas9-TV. **Figure S3.** Diagrams of the Cas9-TV and sgRNA-dsgRNA constructs. **Figure S4.** No indels detected at the dsgRNA target sites. **Figure S5.** Indel patterns generated at the indicated target sites by Cas9, Cas9/dsgRNA and Cas9-TV/dsgRNA. **Figure S6.** Partial genomic DNA sequence of *LOC_Os11g08760* showing sgRNA and dsgRNA target sites. **Figure S7.** Effects of location of proximal dsgRNA on Cas9 editing activity. **Figure S8.** Expression of target genes is not affected when Cas9-TV target coding regions. **Table S1.** Summary of mutagenesis efficiency in rice T0 plants induced by CRISPR/Cas9 at different genomic loci. **Table S2.** Information of the 40 target sites chosen. **Table S3.** Each of the chosen sgRNAs targets two genomic sites with opposite chromatin states. **Table S4.** The chosen sgRNAs and their corresponding proximal dsgRNA targeting sites. **Table S5.** dsgRNA target sequences and their distances to sgRNA34 target site. **Table S6.** Potential off-target sites for the four sgRNAs identified in rice genome. **Table S7.** Primers used in this study. Sequence of the vectors used in this study. (PDF 10459 kb)

Additional file 2: Review history. (DOCX 19 kb)

## Data Availability

Deep sequencing data are available under BioProject IDs PRJNA551128 [42] (https://www.ncbi.nlm.nih.gov/sra/PRJNA551128).
