# Supplementary material for: Modulating chromatin accessibility by transactivation and targeting proximal dsgRNAs enhances Cas9 editing efficiency in vivo
Source: Genome Biol. 2019 Jul 26;20:145. doi: 10.1186/s13059-019-1762-8 (PMC6660936; doi:10.1186/s13059-019-1762-8)
Supplement: Supplementary file 1 — Figure S1. Agarose gels of the PCR products containing the 10 target sites shown in Fig. 2 after incubation with Cas9 ribonucleoprotein (RNP) complexes. Figure S2. Indel patterns generated at the target sites by Cas9 and Cas9-TV. Figure S3. Diagrams of the Cas9-TV and sgRNA-dsgRNA constructs. Figure S4. No indels detected at the dsgRNA target sites. Figure S5. Indel patterns generated at the indicated target sites by Cas9, Cas9/dsgRNA and Cas9-TV/dsgRNA. Figure S6. Partial genomic DNA sequence of LOC_Os11g08760 showing sgRNA and dsgRNA target sites. Figure S7. Effects of location of proximal dsgRNA on Cas9 editing activity. Figure S8. Expression of target genes is not affected when Cas9-TV target coding regions. Table S1. Summary of mutagenesis efficiency in rice T0 plants induced by CRISPR/Cas9 at different genomic loci. Table S2. Information of the 40 target sites chosen. Table S3. Each of the chosen sgRNAs targets two genomic sites with opposite chromatin states. Table S4. The chosen sgRNAs and their corresponding proximal dsgRNA targeting sites. Table S5. dsgRNA target sequences and their distances to sgRNA34 target site. Table S6. Potential off-target sites for the four sgRNAs identified in rice genome. Table S7. Primers used in this study. Sequence of the vectors used in this study. (PDF 10459 kb) [file 13059_2019_1762_MOESM1_ESM.pdf]

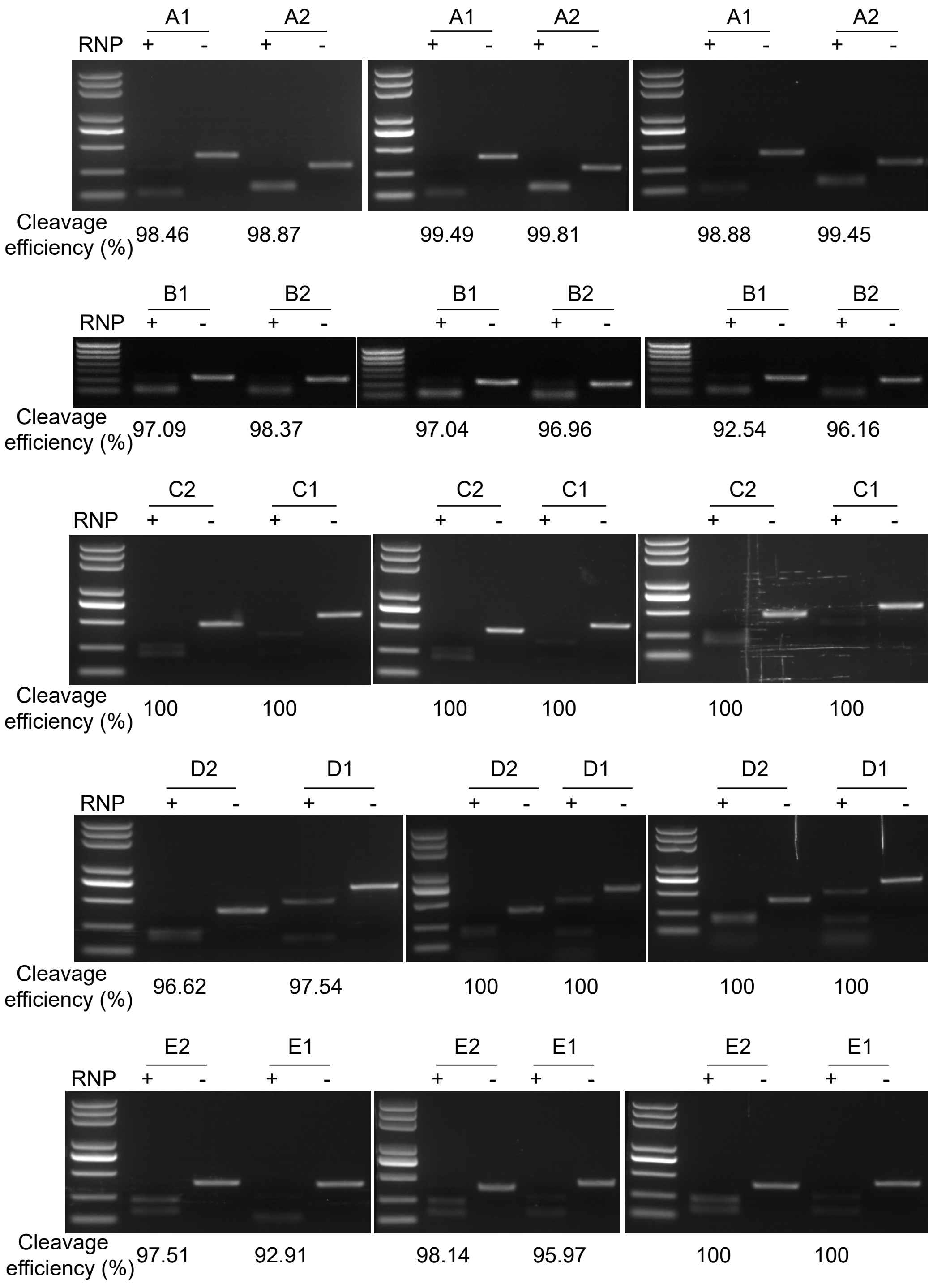

Fig. S1 Agarose gels of the PCR products containing the 10 target sites shown in Fig. 2 after incubation with Cas9 ribonucleoprotein (RNP) complexes. Each experiment was performed three times (n=3).

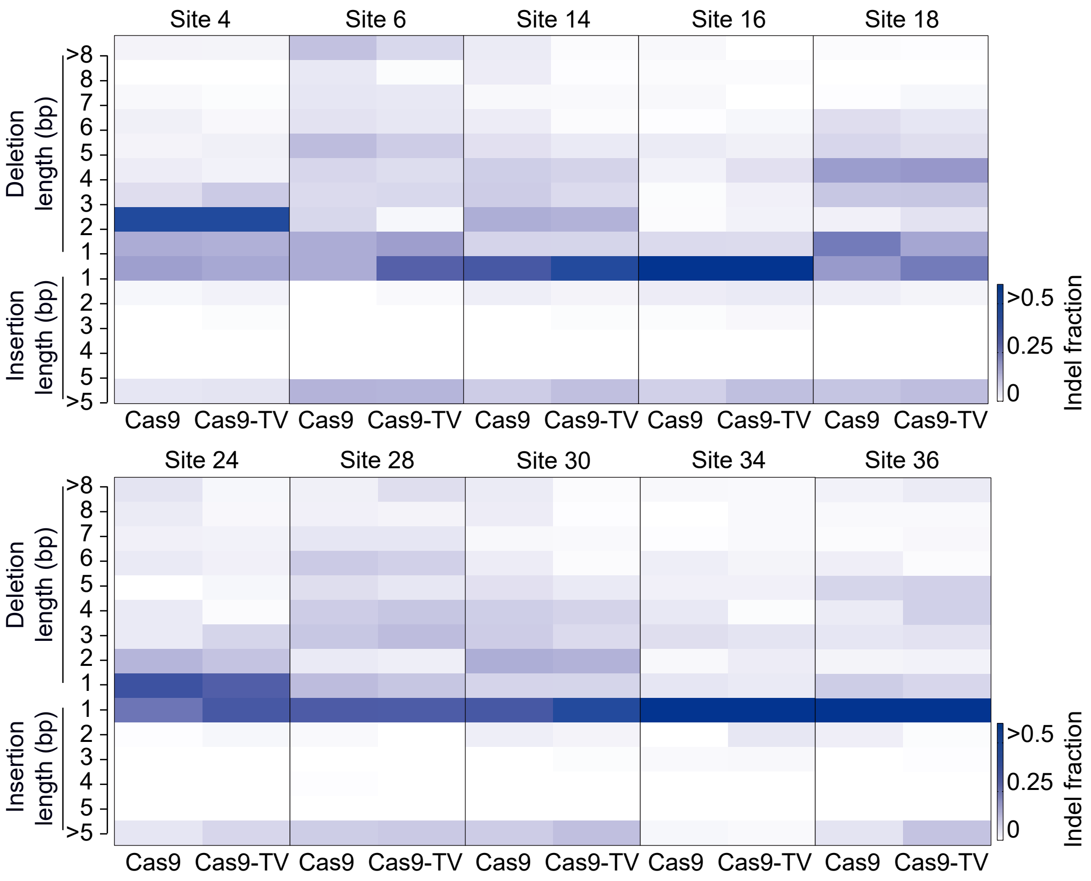

Fig. S2 Indel patterns generated at the target sites by Cas9 and Cas9-TV. The graphs show representative results of one of three independent experiments. All three experiments gave similar results.

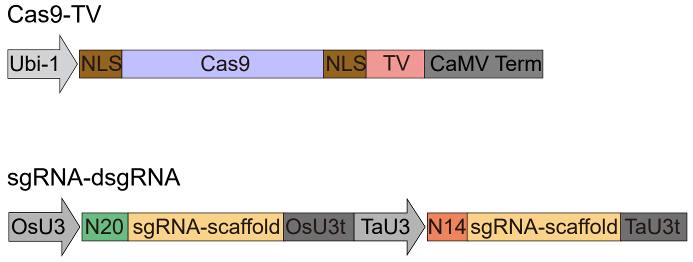

Fig. S3 Diagrams of the Cas9-TV and sgRNA-dsgRNA constructs.

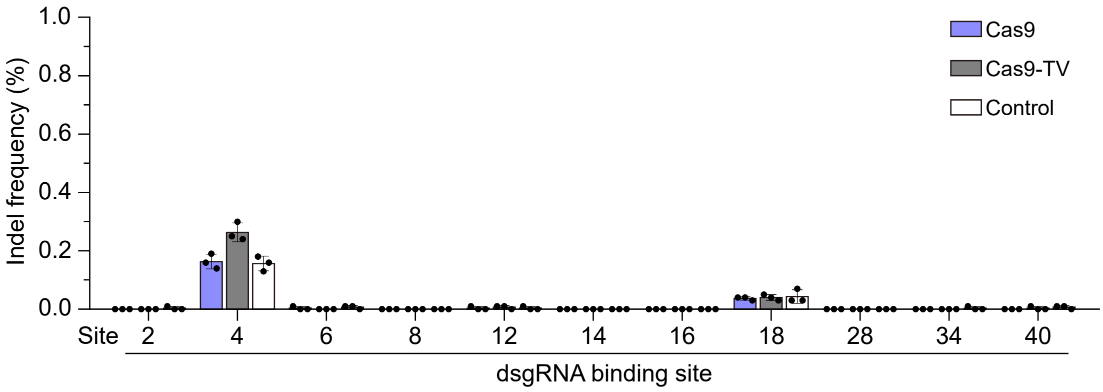

Fig. S4 No indels detected at the dsgRNA target sites. The dsgRNAs were co-transformed into rice protoplasts with Cas9 or Cas9-TV, respectively. Indel frequencies were measured by sequencing targeted amplicons. An untreated protoplast sample was used as control. Data are from sets of three independent biological replicates (n = 3) and are shown as means ± s.e.m.

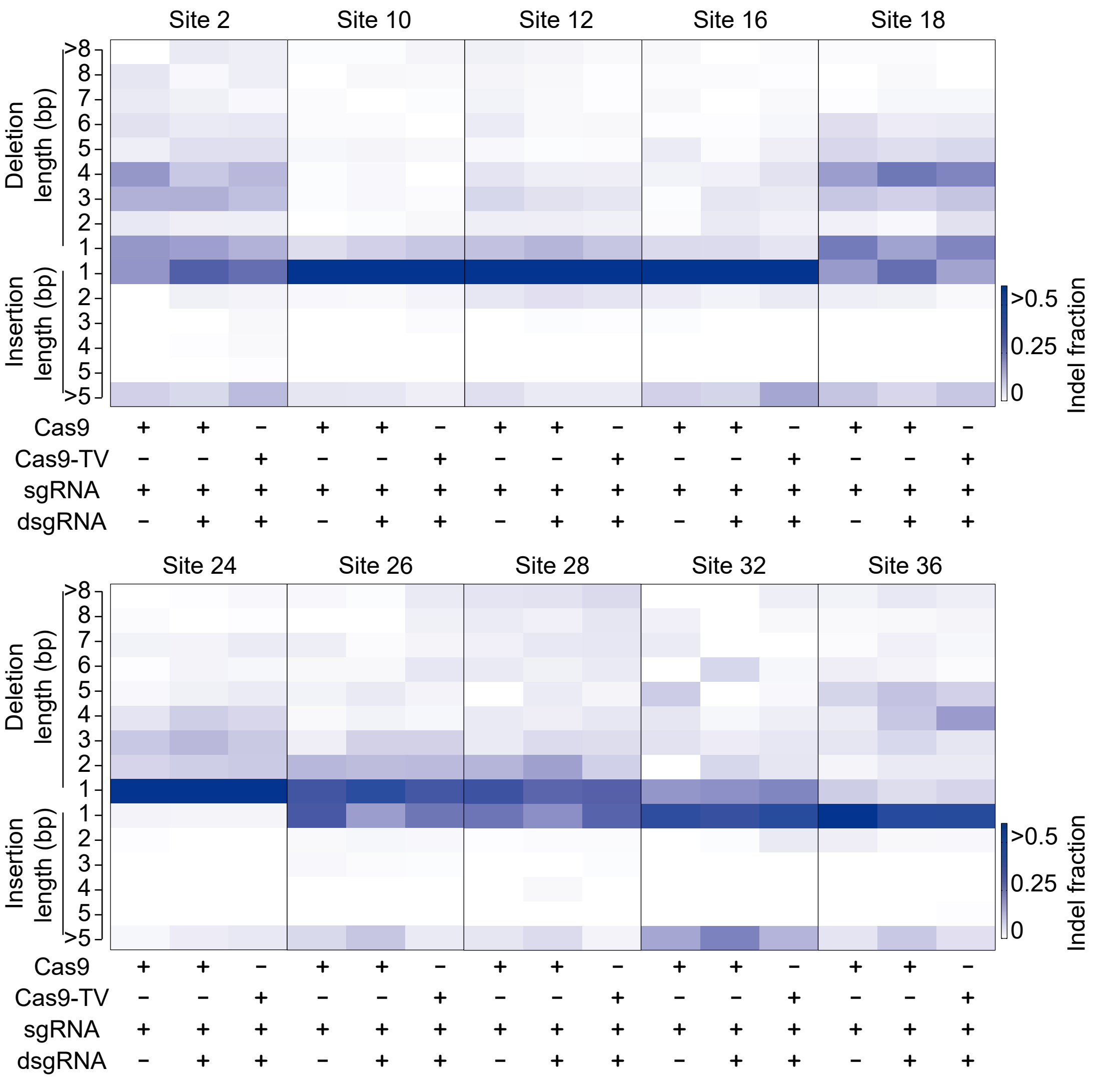

Fig. S5 Indel patterns generated at the indicated target sites by Cas9, Cas9/dsgRNA and Cas9-TV/dsgRNA. The graphs show representative results of one of three independent experiments yielding similar results.

```
5′-GCTCTCCTGATTACTAGCCAGCGATGCATGCAGGGAAAAGAAATTTTCCTACATATATAGAGAGAAAGAGAGTATATA-3′
   ||||||||||||||||||||||||||||||||||||||||||||||||||||||||||||||||||||||||||||||
3′-CGAGAGGACTAATGATCGGTCGCTACGTACGTCCCTTTTCTTTAAAAGGATGTATATATCTCTCTTTCTCTCATATAT-5′
                    dsgRNA5 target site

5′-TATCAAACGACATCATCACCATGTGCATATGTTAGTTACCCTCAATTCCCATCCTAGCAAGCATGGCCGGCTCTTCCT-3′
   ||||||||||||||||||||||||||||||||||||||||||||||||||||||||||||||||||||||||||||||
3′-ATAGTTTGCTGTAGTAGTGGTACACGTATACAATCAATGGGAGTTAAGGGTAGGATCGTTCGTACCGGCCGAGAAGGA-5′

5′-CTCTTGCATCGCCTCGCTCACTACAGCTACCACTCCTCCTCCTTTTCCTCCTCGTTGCCGCTGCGATAGGCAGCTACG-3′
   ||||||||||||||||||||||||||||||||||||||||||||||||||||||||||||||||||||||||||||||
3′-GAGAACGTAGCGGAGCGAGTGATGTCGATGGTGAGGAGGAGGAAAAGGAGGAGCAACGGCGACGCTATCCGTCGATGC-5′
              dsgRNA4 target site

5′-ACCCCAAGGCGTTCTGTTCCAAGACGACAGACGTAGCATCGTGCCTTAAGGTGTTCCCGACACTCCCAGACATCGTCA-3′
   ||||||||||||||||||||||||||||||||||||||||||||||||||||||||||||||||||||||||||||||
3′-TGGGGTTCCGCAAGACAAGGTTCTGCTGTCTGCATCGTAGCACGGAATTCCACAAGGGCTGTGAGGGTCTGTAGCAGT-5′
      dsgRNA3 target site                                              target site 34

5′-CCAAGGCGCAGGACAACCAAGAGTTGTACAAACGGCTGGTCCGCTACTGCTCATTCAAGACCTACGAGGCCACGTCGC-3′
   ||||||||||||||||||||||||||||||||||||||||||||||||||||||||||||||||||||||||||||||
3′-GGTTCCGCGTCCTGTTGGTTCTCAACATGTTTGCCGACCAGGCGATGACGAGTAAGTTCTGGATGCTCCGGTGCAGCG-5′
                                                                   dsgRNA1 target site

5′-TCGCGGAATCCATGATTGCCACCACCACCGCCGCCAACCCCGCGAATATCGCCACCTTCTTCGAGCAGTGGAAAGGGG-3′
   ||||||||||||||||||||||||||||||||||||||||||||||||||||||||||||||||||||||||||||||
3′-AGCGCCTTAGGTACTAACGGTGGTGGTGGCGGCGGTTGGGGCGCTTATAGCGGTGGAAGAAGCTCGTCACCTTTCCCC-5′
                                                                   dsgRNA6 target site

5′-ACGAGGCGATAACGACAAAAACACCTCCAGGCAAGTGCCTCTTGAGCTGCAACAAGACCATCGGCGAAGTTAACGCCA-3′
   ||||||||||||||||||||||||||||||||||||||||||||||||||||||||||||||||||||||||||||||
3′-TGCTCCGCTATTGCTGTTTTTGTGGAGGTCCGTTCACGGAGAACTCGACGTTGTTCTGGTAGCCGCTTCAATTGCGGT-5′

5′-TCCTAACCTGCGGACACACCTACATGGAAGACAGGCCCCCCATCATCCACCAGAACCTCACCGTCTTGTTCCATGGCG-3′
   ||||||||||||||||||||||||||||||||||||||||||||||||||||||||||||||||||||||||||||||
3′-AGGATTGGACGCCTGTGTGGATGTACCTTCTGTCCGGGGGGTAGTAGGTGGTCTTGGAGTGGCAGAACAAGGTACCGC-5′
                    dsgRNA2 target site
```

Fig. S6 Partial genomic DNA sequence of *LOC_Os11g08760* showing sgRNA and dsgRNA target sites. The green line indicates the sgRNA target site and the red lines the dsgRNAs target sites.

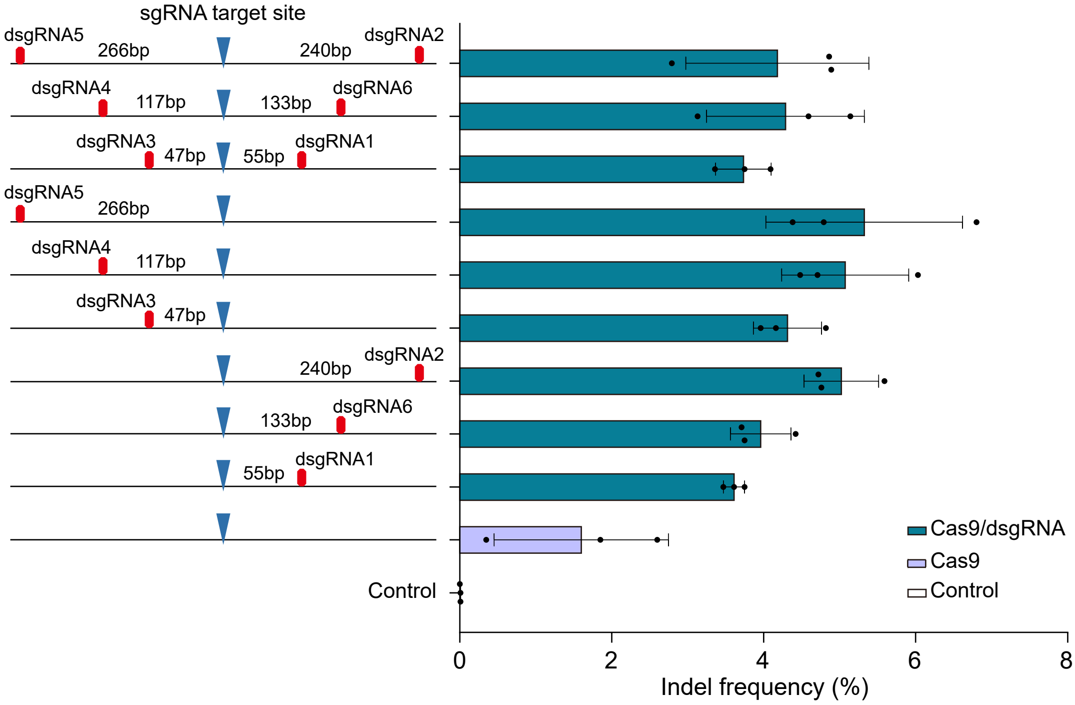

Fig. S7 Effects of location of proximal dsgRNA on Cas9 editing activity. dsgRNA target sites and Cas9-TV target sites are separated from each other by a distance in bp indicated with a number respectively. An untreated protoplast sample was used as control. Indel frequencies were measured by sequencing targeted amplicons. Data are from sets of three independent biological replicates (n = 3) and are shown as means ± s.e.m.

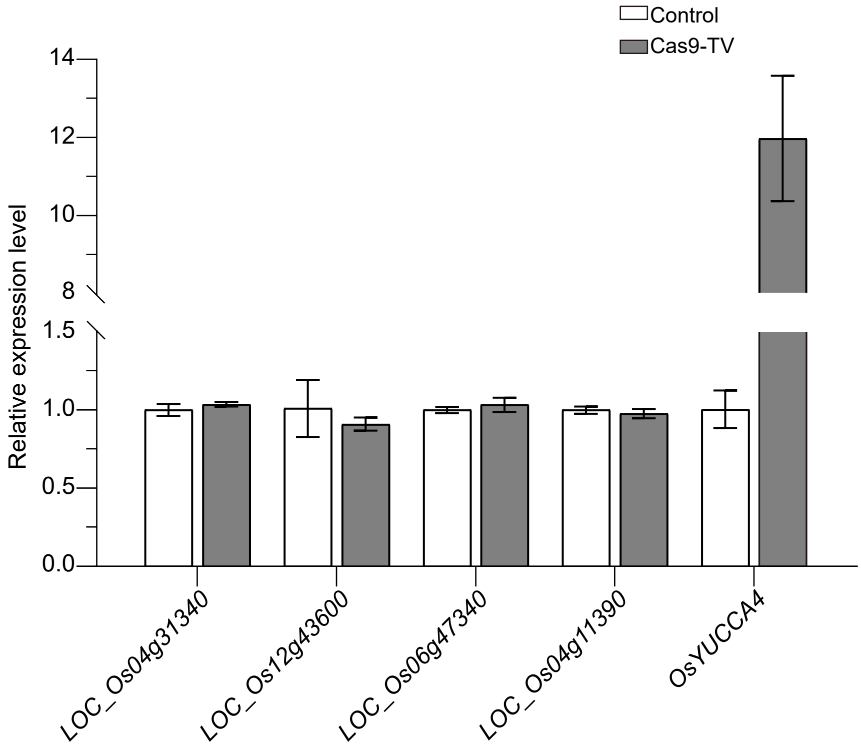

Fig. S8 Expression of target genes is not affected when Cas9-TV targets coding regions. Quantitative gene expression analysis were performed in rice protoplasts transformed with Cas9-TV and corresponding dsgRNA. The dsgRNAs for *LOC_Os04g31340*, *LOC_Os12g43600, LOC_Os06g47340* and *LOC_Os04g11390* are same as Fig. 4 and listed in Table S4 and target coding regions, while the dsgRNA for *OsYUCCA4* targets its promoter region. An untreated protoplasts sample was used as control. Data were normalized to *Ubiquitin* (*Ubi*) expression and the expression level of each gene in the control sample was set at one unit. Error bars indicate SD of three replicates.

Table S1 Summary of mutagenesis efficiency in rice T0 plants induced by CRISPR/Cas9 at different genomic loci

Locus name	Target sequence	Positions	NO. of transgenic lines	NO. of mutant lines	Mutation rate (%)	Chromatin states
LOC_Os03g08550	ATGAGCCAAAACAACCTTGGTGG	chr3:4398956..4398978	22	22	100.00	DH sites
LOC_Os03g17020	AGTGGGCGAGCGTCTCGTACGGG	chr3:9458718..9458740	13	13	100.00	DH sites
LOC_Os02g54600	GAGCAGGATCTGGAGCTCGCCGG	chr2:33443338..33443360	30	29	96.70	DH sites
LOC_Os01g55540	CATGGTGGGGAAAGCTTGGAGGG	chr1:32003246..32003268	42	38	90.50	DH sites
LOC_Os02g54600	CCGGACGACGACGTCGACGACGG	chr2:33443645..33443667	30	25	83.30	DH sites
LOC_Os02g54600	GACGTCGGCGAGGAAGGCCTCGG	chr2:33443272..33443294	30	24	80.00	DH sites
LOC_Os01g55540	CGGACGACGACGCCATGGTGGGG	chr1:32003233..32003255	42	33	78.60	DH sites
LOC_Os05g49140	GAGGGTGGTGAGGAATCCGATGG	chr5:28189687..28189709	21	16	76.20	DH sites
LOC_Os02g46760	GCACGGACAGCCGCAGCTGCCGG	chr2:28552759..28552781	24	17	70.80	DH sites
LOC_Os02g48000	AATTTTAACCGTGATCAGAATGG	chr2:29375178..29375200	25	15	60.00	DH sites
LOC_Os02g46760	GGCCACGCGGAGGTGCGCGCCGG	chr2:28552992..28553014	24	14	58.30	DH sites
LOC_Os02g53700	ATCAGCTGCAGTGTCCTGCCAGG	chr2:32864545..32864567	16	8	50.00	DH sites
LOC_Os01g04409	AGAAGCAAGAGTCACATAATAGG	chr1:1959592..1959614	22	9	40.91	DH sites
LOC_Os03g05200	AATTCCATGGCCGCGCAGTTGGG	chr3:2525483..2525505	44	15	34.09	DH sites
LOC_Os08g33370	AACTATCTGTGGCAGGAGGACGG	chr8:20812965..20812987	23	6	26.09	DH sites
LOC_Os02g46760	ACAGCATCTCGAGCTCCGCCAGG	chr2:28552858..28552880	24	6	25.00	DH sites
LOC_Os12g01200	ATGATGTAAAAATAAATAGCTGG	chr12:114104..114126	24	3	12.50	Non-DH sites
LOC_Os10g25090	AATATAGCAAACCACTCAGCTGG	chr10:12924597..12924616	27	21	77.78	Non-DH sites
LOC_Os01g47530	AGCTTGAGAAAGATCGCATGCGG	chr1:27177431..27177453	153	24	13.70	Non-DH sites
LOC_Os02g53700	AGACATCCGATGTACATTCCAGG	chr2:32862128..32862150	27	3	11.11	Non-DH sites

LOC_Os05g38950	AAACAGGATTGCAAGAGTTAAGG	chr5:22838317..22838339	22	8	36.36	Non-DH sites
LOC_Os03g08550	AACCAATGTGGCACCTATAAAGG	chr3:4400430..4400452	16	3	18.75	Non-DH sites
LOC_Os03g08550	ATGGACAACCTTGTTGGGGAAGG	chr3:4400503..4400525	34	32	94.12	Non-DH sites
LOC_Os10g25090	AGAGAAAACGCAAAGGTACAAGG	chr10:12925639..12925661	14	13	92.86	Non-DH sites
LOC_Os11g17080	TGTGCGAATAGAGTTCCTCAAGG	chr11:9477665..9477687	12	9	75.00	Non-DH sites
LOC_Os07g07194	AAAGACCTGTTACAGGAATCCGG	chr7:3562066..3562088	17	7	41.18	Non-DH sites
LOC_Os07g07194	ACTACGAACCAATTGGCTCATGG	chr7:3561913..3561935	44	20	45.45	Non-DH sites
LOC_Os06g26340	GAAAATATCTGACATGCATGCGG	chr6:15417435..15417457	23	7	30.40	Non-DH sites
LOC_Os02g49070	AGCAGTATCTCTAGCGCTTCAGG	chr2:30000058..30000080	44	30	68.18	Non-DH sites
LOC_Os02g57080	ACGCGTCTACCATGGGACCATGG	chr2:34947055..34947077	20	7	60.00	Non-DH sites
LOC_Os02g06930	TATTGCTTGCTTTGATATCCCGG	chr2:3506669..3506691	48	27	56.30	Non-DH sites
LOC_Os05g05160	GATATAATTACAGATCTACTAGG	chr5:2515783..2515805	109	20	18.30	Non-DH sites
LOC_Os08g33370	ATCTCCGAGTTGGATACCCTCGG	chr8:20813145..20813167	15	9	60.00	Non-DH sites
LOC_Os02g06930	CTATCTTTAGAGTCAAGACAGGG	chr2:3506268..3506290	48	19	39.60	Non-DH sites
LOC_Os02g04230	AACCACATAATTCAGGAGCTCGG	chr2:1853822..1853844	48	8	16.70	Non-DH sites
LOC_Os10g38950	AGGGCTAAAATATGTGCACTCGG	chr10:20758294..20758316	96	14	14.60	Non-DH sites
LOC_Os02g53700	AAAATCCTCTGAATCAAGTGTGG	chr2:32865365..32865387	64	39	60.94	Non-DH sites
LOC_Os04g56160	AACCTGTTGGTCCTCTTGATCGG	chr4:33461383..33461405	48	11	22.92	Non-DH sites
LOC_Os01g32660	GTGGTATCGTCCAACTAGTTCGG	chr1:17906168..17906190	87	27	31.00	Non-DH sites
LOC_Os04g38600	CTCATCACCGCCCCCGGCAAGGG	chr4:22937954..22937976	42	11	26.20	Non-DH sites
LOC_Os06g48590	CTGAATATGTCGTGACCCGGTGG	chr6:29401605..29401627	128	29	22.70	Non-DH sites
LOC_Os11g17080	ACTGCGACACCCAGATATCGTGG	chr11:9472567..9472589	12	9	75.00	Non-DH sites
LOC_Os05g34070	GTAGCAGATAGCTTAGTAGATGG	chr5:20128891..20128913	36	24	66.70	Non-DH sites
LOC_Os04g38600	GCAGCGACGACGAGCTCCTCAGG	chr4:22937368..22937390	42	21	50.00	Non-DH sites
LOC_Os01g45620	CGTCGAAGGACACTCGAGCAAGG	chr1:25922196..25922218	133	41	30.80	Non-DH sites

LOC_Os08g33370	ATCTACTGCTGCAGAATCTAAGG	chr8:20814142..20814164	22	4	18.18	Non-DH sites
LOC_Os04g56160	AGCTGAAATGTACGCGCGAGGGG	chr4:33459738..33459760	22	5	22.73	Non-DH sites
LOC_Os10g35450	AAGCCACGCGATATCGGTGCAGG	chr10:18971698..18971720	40	2	5.00	Non-DH sites
LOC_Os10g35450	AAACATTACGAGAAAGATTCAGG	chr10:18971723..18971745	38	10	26.32	Non-DH sites
LOC_Os10g35450	AAGGCAGCTCGGGCAATGCTAGG	chr10:18971856..18971878	24	7	29.17	Non-DH sites
LOC_Os04g59320	ATGACGTTGTTGGAAGCTGCAGG	chr4:35271894..35271916	44	15	34.10	Non-DH sites
LOC_Os04g59320	AAGTGCTTCTTCGGTGTACAAGG	chr4:35272085..35272107	13	2	15.38	Non-DH sites
LOC_Os06g49430	GTCGTCGGCGACCTCGAGGTCGG	chr6:29950247..29950269	13	9	69.20	Non-DH sites
LOC_Os03g11614	GCAGAGGAGGGAGAGCTCGTAGG	chr3:6061158..6061180	78	53	67.90	Non-DH sites
LOC_Os03g11614	CTCGAAGAGGCGGCCGCGGCCGG	chr3:6061107..6061129	78	51	65.40	Non-DH sites
LOC_Os10g30860	GAAGAGGACCCTCCCGGCGAAGG	chr10:16092786..16092808	83	45	54.10	Non-DH sites
LOC_Os07g14700	GATGGTCTCCCGGGTGTCGTAGG	chr7:8382918..8382940	112	46	40.10	Non-DH sites
LOC_Os07g14700	CAAGAACACCAAGGACCTGGAGG	chr7:8382683..8382705	112	39	34.80	Non-DH sites
LOC_Os06g09180	CCGGAGTTCAGAAGCTTCGTCGG	chr6:4617592..4617614	94	16	17.00	Non-DH sites
LOC_Os01g04409	AGGTACCCATTCTGGCTAGGCGG	chr1:1967198..1967220	79	1	1.30	Non-DH sites
LOC_Os01g62430	AACGACTTCCTAGGTGAGATAGG	chr1:36136005..36136027	44	2	4.55	Non-DH sites
LOC_Os03g14260	ATGTACAAATCTGCTCTTGAGGG	chr3:7762634..7762656	14	13	92.86	Non-DH sites
LOC_Os03g21540	CCTCAGGCTCCACTTCTACCCGG	chr3:12309229..12309251	12	1	8.33	Non-DH sites
LOC_Os03g21540	ATCTCCCCCTCCAAGGACACGGG	chr3:12309614..12309636	21	8	38.10	Non-DH sites
LOC_Os01g56330	CCTCAGGCTCCACTTCTACCCGG	chr1:32462465..32462487	12	1	8.33	Non-DH sites
LOC_Os01g56330	ATCTCCCCCTCCAAGGACACGGG	chr1:32462850..32462872	21	6	28.57	Non-DH sites
LOC_Os06g26340	TCAGCATCATGTTTCTAGAGAGG	chr6:15415163..15415185	23	14	60.90	Non-DH sites
LOC_Os02g49070	ATAGATTCCATGCTTGAGATTGG	chr2:30000475..30000497	28	23	82.14	Non-DH sites
LOC_Os05g49140	GATGGATACAGACCTCCACCAGG	chr5:28192421..28192443	21	6	28.60	Non-DH sites
LOC_Os02g49070	AAGTCAAACAAGCCTGTTCTAGG	chr2:30001366..30001388	34	31	91.18	Non-DH sites

Table S2 Information of the 40 target sites chosen

sgRNA	Target site	Locus name	Target sequence	Region	Chromatin state
sgRNA1	Site 1	LOC_Os04g31340	GCACACATGTCGTCGTACGGTGG	promoter	open chromatin
sgRNA2	Site 2		GCACGGCTGGTTCTGCCGTAGGG	exon	
sgRNA3	Site 3	LOC_Os04g33830	TCAGCTAGTAGTTCCTACCGAGG	promoter	open chromatin
sgRNA4	Site 4		ACGAGGAACCTGGAGAGGAACGG	exon	
sgRNA5	Site 5	LOC_Os04g58720	TACTATTCAGTCCAGCCGCGCGG	promoter	open chromatin
sgRNA6	Site 6		CCTTGTCCTTGATAGCCGTGCGG	exon	
sgRNA7	Site 7	LOC_Os04g41560	CCATCACACAAACCGCCGCGCGG	promoter	open chromatin
sgRNA8	Site 8		GGCCACGGTGGTGTGCTGCGCGG	exon	
sgRNA9	Site 9	LOC_Os04g42020	CTACCTACCATGCCTCGCCGCGG	promoter	open chromatin
sgRNA10	Site 10		CCCGAGCTCGACATCACCGGCGG	exon	
sgRNA11	Site 11	LOC_Os02g02890	TCCCTAACCCAACCACCCCACGG	promoter	open chromatin
sgRNA12	Site 12		TGTACGCGAAGGACGTGCCGCGG	exon	
sgRNA13	Site 13	LOC_Os05g41060	GCCCTCACCACAAACCCACGTGG	promoter	open chromatin
sgRNA14	Site 14		TCTCCTTCTTGGCGAAGAGGCGG	exon	
sgRNA15	Site 15	LOC_Os12g43600	GTGCCTTTGGAATATACGGGGGG	promoter	open chromatin
sgRNA16	Site 16		CGAGGCCGCCGACGAAGCAGCGG	exon	
sgRNA17	Site 17	LOC_Os06g47340	TCACCTCACCCGCACGACGACGG	promoter	open chromatin
sgRNA18	Site 18		GCAGCACCGGTTCTACCAAGGGG	exon	
sgRNA19	Site 19	LOC_Os08g03290	TGTCATTAGCTAGTGACGCGCGG	promoter	open chromatin
sgRNA20	Site 20		GAGTATGTCGTGGAGTCCACCGG	exon	
sgRNA21	Site 21	LOC_Os04g22730	GGTGGGGTGGGACTTATGGATGG	promoter	closed chromatin
sgRNA22	Site 22		AGGGGTGTATCGATGATGCCCGG	exon	
sgRNA23	Site 23	LOC_Os04g18650	GAGATGATGCGTGTGCCGGCCGG	promoter	closed chromatin

sgRNA24	Site 24		ACGGCCGCCTCCGTACGCCGCGG	exon	
sgRNA25	Site 25	LOC_Os04g11390	GGACTGGAGGACCTATTCACAGG	promoter	closed chromatin
sgRNA26	Site 26		GGAACAGCCTCATCCCTTGGCGG	exon	
sgRNA27	Site 27	LOC_Os04g12220	GGGATACCTGTGACGAGCCGCGG	promoter	closed chromatin
sgRNA28	Site 28		GTCTTTGGACGTAGCCATGGTGG	exon	
sgRNA29	Site 29	LOC_Os04g13530	GCTGTGGAGTTGAAGCACAGCGG	promoter	closed chromatin
sgRNA30	Site 30		TGTGCTGCCGAACATCAACCCGG	exon	
sgRNA31	Site 31	LOC_Os04g13640	CCAAGCATCTCAGCACCGGCGGG	promoter	closed chromatin
sgRNA32	Site 32		CCATGACATTGTCCGAGCCACGG	exon	
sgRNA33	Site 33	LOC_Os11g08760	ATTACTCCGCATGTGACCACCGG	promoter	closed chromatin
sgRNA34	Site 34		AGACATCGTCACCAAGGCGCAGG	exon	
sgRNA35	Site 35	LOC_Os07g43820	TCATCCCTCGCCATCGGCGGCGG	promoter	closed chromatin
sgRNA36	Site 36		CTCGCTCCCGACCAACCAGTCGG	exon	
sgRNA37	Site 37	LOC_Os09g24280	ACACTCAACCAGATGTTACGTGG	promoter	closed chromatin
sgRNA38	Site 38		TGGGTAATGGTGATATCCCATGG	exon	
sgRNA39	Site 39	LOC_Os07g43850	ACGTATCGTCGAACTTGTACCGG	promoter	closed chromatin
sgRNA40	Site 40		CGACGATGACCATGTCCACGTGG	exon	

Table S3 Each of the chosen sgRNAs targets two genomic sites with opposite chromatin states

	Target site	Target sequence	Positon	Locus name	Region	Chromatin states
sgRNA A	A1	ACGCAAACGAATCGAGGAGTAGG	chr1:209699..209721	--	Intergenic	DH sites
	A2	ACGCAAACGAATCGAGGAGTAGG	chr2:33815506..33815528	--	Intergenic	Non-DH sites
sgRNA B	B1	ATCGCCATCGCCATCGCCGCAGG	chr9:20073880..20073858	LOC_Os09g34000	CDS	DH site
	B2	ATCGCCATCGCCATCGCCGCCGG	chr1:23313634..23313612	LOC_Os01g41180	CDS	non-DH site
sgRNA C	C1	CGAAGGCGGCGGCGGCGATCTGG	chr1:1179280..1179299	LOC_Os01g03070	UTR	DH sites
	C2	CGAAGGCGGCGGCGGCGATCCGG	chr7:22797802..22797824	LOC_Os07g37990	CDS	Non-DH sites
sgRNA D	D1	TCTTCTTCTAATCTTGCTGTTGG	chr1:1103803..1103825	LOC_Os01g02960	UTR	DH sites
	D2	TCTTCTTCTAATCTTGCTGTTGG	chr1:1319901..1319923	--	Intergenic	Non-DH sites
sgRNA E	E1	GTTGCTGCTGCTGCTGCGATGG	chr1:1791437..1791458	LOC_Os01g04110	UTR	DH sites
	E2	GTTGCTGCTGCTGCTGCGACGG	chr1:198166..198187	LOC_Os01g01390	CDS	Non-DH sites

Table S4 The chosen sgRNAs and their corresponding proximal dsgRNA targeting sites

sgRNA [a]	dsgRNA target sequence [b]	Distance [c]
sgRNA 2	GACATCATCTGGCAGGG	50bp
sgRNA 4	TGCAGGCTTCACGACGG	32bp
sgRNA 6	TGACCTGATGCCCAAGG	55bp
sgRNA 8	GCGCTGGTGCTTGCTGG	57bp
sgRNA 10	CTTCGCGCGCTCCATGG	35bp
sgRNA 12	GGCGTGGGCAAGAGCGG	39bp
sgRNA 14	TACAAGCTCAAGCTCGG	50bp
sgRNA 16	GGACCTTGGACTCGAGG	55bp
sgRNA 18	ACCTGATTGGGTGAAGG	60bp
sgRNA 20	TATGGTAGCGAGCGTGG	68bp
sgRNA 22	AACAGCTAGGCTCTTGG	39bp
sgRNA 24	ACTGCAGGCGCTGCAGG	59bp
sgRNA 26	ACTCATCGGTGTGTAGG	92bp
sgRNA 28	GTTGATGGACGAGGTGG	61bp
sgRNA 30	AGCAGCACGTGCCTCGG	62bp
sgRNA 32	GGCCAACTGAACGACGG	56bp
sgRNA 34	GGCCACGTCGCTCGCGG	55bp
sgRNA 36	CCGATGCAGCCCACCGG	66bp
sgRNA 38	GCGCATTAGACCAAGGG	83bp
sgRNA 40	GGCGCGACCAACCACGG	40bp

a, sgRNAs are same to table S2; b, 14nt guide sequence + PAM; c, dsgRNA target sites and Cas9-TV target sites are separated from each other by a distance in bp indicated with a number.

Table S5 dsgRNA target sequences and their distances to sgRNA34 target site

	dsgRNA	dsgRNA target sequence [a]	Distance [b]
Single dsgRNA	dsgRNA1	GGCCACGTCGCTCGCGG	55
	dsgRNA2	CCTACATGGAAGACAGG	240
	dsgRNA3	GGAACAGAACGCCTTGG	47
	dsgRNA4	AGCTGTAGTGAGCGAGG	117
	dsgRNA5	CAGCGATGCATGCAGGG	266
	dsgRNA6	GTGGAAAGGGGACGAGG	133
Paired dsgRNA	dsgRNA1&3	GGCCACGTCGCTCGCGG	55
		GGAACAGAACGCCTTGG	47
	dsgRNA2&5	CCTACATGGAAGACAGG	240
		CAGCGATGCATGCAGGG	266
	dsgRNA4&6	GGAACAGAACGCCTTGG	117
		GTGGAAAGGGGACGAGG	133

a, 14nt guide sequence + PAM; b, dsgRNA target sites and Cas9-TV target sites are separated from each other by a distance in bp indicated with a number.

Table S6 Potential off-target sites for the four sgRNAs identified in rice genome

Target site	Sequence[a]	Target gene loci
Site 24	ACGGCCGCCTCCGTACGCCG**CGG**	LOC_Os04g18650
OT24-1	ACGGCCGCTTCCGCACGCCG**CGG**	LOC_Os03g05590
OT24-2	CCGCTCGCCCCCGTACGCCG**CGG**	LOC_Os06g11400
OT24-3	GCGGCCGCGGCCGTACGCTG**GGG**	LOC_Os01g73410
Site 28	GTCTTTGGACGTAGCCATGG**TGG**	LOC_Os04g12220
OT28-1	GTCTTTGCACATAGCCATGG**CGG**	LOC_Os05g04110
OT28-2	GTCTTTTGATGCAGCAATGG**AGG**	LOC_Os01g56140
OT28-3	GTTTTTGGACTTAGCCAAGG**AGG**	LOC_Os04g57390
Site 34	AGACATCGTCACCAAGGCGC**AGG**	LOC_Os11g08760
OT34-1	CGACGCCGACACCAAGGCGC**TGG**	LOC_Os04g56110
OT34-2	GGACGTCCTCGCCAAGGCGC**AGG**	LOC_Os09g38050
OT34-3	GGACATCGTCGTCGAGGCGC**TGG**	LOC_Os04g32010
OT34-4	CGACGTCGTGACCAAGGTGC**CGG**	LOC_Os11g04940
OT34-5	AGTCATCCTCAACAAGGCCC**AGG**	LOC_Os02g14059
Site 38	TGGGTAATGGTGATATCCCA**TGG**	LOC_Os09g24280
OT38-1	TAGGTGATGATGATATACCA**AGG**	LOC_Os12g29220
OT38-2	TAGGTAGTTGTGATATCACA**GGG**	LOC_Os12g39430
OT38-3	TGGGTGATGATGATATCCAT**CGG**	LOC_Os03g37411
OT38-4	TATGTGATGGTGATATCCTA**CGG**	LOC_Os12g40790

a, The mismatch bases are shown in red. The PAM motif is written in bold.

Table S7 Primers used in this study.

Primer name	Primer sequence (5'-3')	Application
Cas9-F	GAGGATCCCCATGGCCCCTAAGAAGAAGAGAAAG	Constructing 163-Cas9-TV
Cas9-64-R	TCCAGGGCGTCTGAGCCCCTAGGCTTCTTCTTCTTCGCTTG TCCTG	
64-Cas9-F	GCGAAGAAGAAGAAGCCTAGGGGCTCAGACGCCCTG	
64-R	CGCAATTGCTACCTAGCGAGCATGTCGAGGTCGAAG	
S1-SG-F	GGCAGCACACATGTCGTCGTACGG	Constructing vector expressing sgRNA for Site 1
S1-SG-R	AAACCCGTACGACGACATGTGTGC	
S2-SG-F	GGCAGCACGGCTGGTTCTGCCGTA	Constructing vector expressing sgRNA for Site 2
S2-SG-R	AAACTACGGCAGAACCAGCCGTGC	
S3-SG-F	GGCATCAGCTAGTAGTTCCTACCG	Constructing vector expressing sgRNA for Site 3
S3-SG-R	AAACCGGTAGGAACTACTAGCTGA	
S4-SG-F	GGCACGAGGAACCTGGAGAGGAA	Constructing vector expressing sgRNA for Site 4
S4-SG-R	AAACTTCCTCTCCAGGTTCCTCG	
S5-SG-F	GGCATACTATTCAGTCCAGCCGCG	Constructing vector expressing sgRNA for Site 5
S5-SG-R	AAACCGCGGCTGGACTGAATAGTA	
S6-SG-F	GGCACCTTGTCCTTGATAGCCGTG	Constructing vector expressing sgRNA for Site 6
S6-SG-R	AAACCACGGCTATCAAGGACAAGG	
S7-SG-F	GGCACCATCACACAAACCGCCGCG	Constructing vector expressing sgRNA for Site 7
S7-SG-R	AAACCGCGGCGGTTTGTGTGATGG	
S8-SG-F	GGCAGGCCACGGTGGTGTGCTGCG	Constructing vector expressing sgRNA for Site 8
S8-SG-R	AAACCGCAGCACACCACCGTGGCC	
S9-SG-F	GGCACTACCTACCATGCCTCGCCG	Constructing vector expressing sgRNA for Site 9
S9-SG-R	AAACCGGCGAGGCATGGTAGGTAG	

S10-SG-F	GGCACCCGAGCTCGACATCACCGG	Constructing vector expressing sgRNA for Site 10
S10-SG-R	AAACCCGGTGATGTCGAGCTCGGG	
S11-SG-F	GGCATCCCTAACCCAACCACCCCA	Constructing vector expressing sgRNA for Site 11
S11-SG-R	AAACTGGGGTGGTTGGGTTAGGGA	
S12-SG-F	GGCATGTACGCGAAGGACGTGCCG	Constructing vector expressing sgRNA for Site 12
S12-SG-R	AAACCGGCACGTCCTTCGCGTACA	
S13-SG-F	GGCAGCCCTCACCACAAACCCACG	Constructing vector expressing sgRNA for Site 13
S13-SG-R	AAACCGTGGGTTTGTGGTGAGGGC	
S14-SG-F	GGCATCTCCTTCTTGGCGAAGAGG	Constructing vector expressing sgRNA for Site 14
S14-SG-R	AAACCCTCTTCGCCAAGAAGGAGA	
S15-SG-F	GGCAGTGCCTTTGGAATATACGGG	Constructing vector expressing sgRNA for Site 15
S15-SG-R	AAACCCCGTATATTCCAAAGGCAC	
S16-SG-F	GGCACGAGGCCGCCGACGAAGCAG	Constructing vector expressing sgRNA for Site 16
S16-SG-R	AAACCTGCTTCGTCGGCGGCCTCG	
S17-SG-F	GGCATCACCTCACCCGCACGACGA	Constructing vector expressing sgRNA for Site 17
S17-SG-R	AAACTCGTCGTGCGGGTGAGGTGA	
S18-SG-F	GGCAGCAGCACCGGTTCTACCAAG	Constructing vector expressing sgRNA for Site 18
S18-SG-R	AAACCTTGGTAGAACCGGTGCTGC	
S19-SG-F	GGCATGTCATTAGCTAGTGACGCG	Constructing vector expressing sgRNA for Site 19
S19-SG-R	AAACCGCGTCACTAGCTAATGACA	
S20-SG-F	GGCAGAGTATGTCGTGGAGTCCAC	Constructing vector expressing sgRNA for Site 20
S20-SG-R	AAACGTGGACTCCACGACATACTC	
S21-SG-F	GGCAGGTGGGGTGGGACTTATGGA	Constructing vector expressing sgRNA for Site 21
S21-SG-R	AAACTCCATAAGTCCCACCCCACC	
S22-SG-F	GGCAGGGGTGTATCGATGATGCC	Constructing vector expressing sgRNA for Site 22

S22-SG-R	AAACGGCATCATCGATACACCCCT	
S23-SG-F	GGCAGAGATGATGCGTGTGCCGGC	Constructing vector expressing sgRNA for Site 23
S23-SG-R	AAACGCCGGCACACGCATCATCTC	
S24-SG-F	GGCACGGCCGCCTCCGTACGCCG	Constructing vector expressing sgRNA for Site 24
S24-SG-R	AAACCGGCGTACGGAGGCGGCCG	
S25-SG-F	GGCAGGACTGGAGGACCTATTCAC	Constructing vector expressing sgRNA for Site 25
S25-SG-R	AAACGTGAATAGGTCCTCCAGTCC	
S26-SG-F	GGCAGGAACAGCCTCATCCCTTGG	Constructing vector expressing sgRNA for Site 26
S26-SG-R	AAACCCAAGGGATGAGGCTGTTCC	
S27-SG-F	GGCAGGGATACCTGTGACGAGCCG	Constructing vector expressing sgRNA for Site 27
S27-SG-R	AAACCGGCTCGTCACAGGTATCCC	
S28-SG-F	GGCAGTCTTTGGACGTAGCCATGG	Constructing vector expressing sgRNA for Site 28
S28-SG-R	AAACCCATGGCTACGTCCAAAGAC	
S29-SG-F	GGCAGCTGTGGAGTTGAAGCACAG	Constructing vector expressing sgRNA for Site 29
S29-SG-R	AAACCTGTGCTTCAACTCCACAGC	
S30-SG-F	GGCATGTGCTGCCGAACATCAACC	Constructing vector expressing sgRNA for Site 30
S30-SG-R	AAACGGTTGATGTTCGGCAGCACA	
S31-SG-F	GGCACCAAGCATCTCAGCACCGGC	Constructing vector expressing sgRNA for Site 31
S31-SG-R	AAACGCCGGTGCTGAGATGCTTGG	
S32-SG-F	GGCACCATGACATTGTCCGAGCCA	Constructing vector expressing sgRNA for Site 32
S32-SG-R	AAACTGGCTCGGACAATGTCATGG	
S33-SG-F	GGCATTACTCCGCATGTGACCAC	Constructing vector expressing sgRNA for Site 33
S33-SG-R	AAACGTGGTCACATGCGGAGTAA	
S34-SG-F	GGCAGACATCGTCACCAAGGCGC	Constructing vector expressing sgRNA for Site 34
S34-SG-R	AAACGCGCCTTGGTGACGATGTC	

S35-SG-F	GGCATCATCCCTCGCCATCGGCGG	Constructing vector expressing sgRNA for Site 35
S35-SG-R	AAACCCGCCGATGGCGAGGGATGA	
S36-SG-F	GGCACTCGCTCCCGACCAACCAGT	Constructing vector expressing sgRNA for Site 36
S36-SG-R	AAACACTGGTTGGTCGGGAGCGAG	
S37-SG-F	GGCACACTCAACCAGATGTTACG	Constructing vector expressing sgRNA for Site 37
S37-SG-R	AAACCGTAACATCTGGTTGAGTG	
S38-SG-F	GGCATGGGTAATGGTGATATCCCA	Constructing vector expressing sgRNA for Site 38
S38-SG-R	AAACTGGGATATCACCATTACCCA	
S39-SG-F	GGCACGTATCGTCGAACTTGTAC	Constructing vector expressing sgRNA for Site 39
S39-SG-R	AAACGTACAAGTTCGACGATACG	
S40-SG-F	GGCACGACGATGACCATGTCCACG	Constructing vector expressing sgRNA for Site 40
S40-SG-R	AAACCGTGGACATGGTCATCGTCG	
S1-F	GTGTCTCTAACCTTGCAGCAAG	1st PCR for deep sequencing of Site 1
S1-R	CGAAAGGCGAAATGCTGATCC	
S2-F	CCCATGCATGGGAAGTACCTCG	1st PCR for deep sequencing of Site 2
S2-R	CGCCAACCTATGGTGGCACAG	
S3-F	CAGTCGTAACCGACTAGAATCCCG	1st PCR for deep sequencing of Site 3
S3-R	TTGTGGCAGCGTAGGTTAGGC	
S4-F	CGGTGCGTGCGTCACAGTCA	1st PCR for deep sequencing of Site 4
S4-R	CGCGATCCACCCGATCAGCC	
S5-F	CCGGGTCATCCTGCCTCTGAG	1st PCR for deep sequencing of Site 5
S5-R	TCGATGGGCCGAAACGCTAGC	
S6-F	CCCTTGTTGATGCTACAGATGTTC	1st PCR for deep sequencing of Site 6
S6-R	GAACATCCCACGCTTCTCCAG	
S7-F	AGTGCGCGTTCTCGTTCTCACG	1st PCR for deep sequencing of Site 7

S7-R	CGTCGCACTGGATCCTCATGCC	
S8-F	GCAGCCTAGCCATCCGGTGT	1st PCR for deep sequencing of Site 8
S8-R	ACCAGTGCCTCAGCGACACG	
S9-F	GGCATGCCTAAGAAAATAGGCC	1st PCR for deep sequencing of Site 9
S9-R	AATGTGCTCGTCGTTGTCGAG	
S10-F	GGGCAGAAAGATGGCGCCGTC	1st PCR for deep sequencing of Site 10
S10-R	GCTTCTCCCTGTACCGCATCAGC	
S11-F	GAATATCGCCCACTCGCTCA	1st PCR for deep sequencing of Site 11
S11-R	TTCACTTCAGATCGCTGCGT	
S12-F	CAGGCCACGCAGCGATCTGAA	1st PCR for deep sequencing of Site 12
S12-R	CCGTAGATCGACTCCCCTCCCG	
S13-F	ACCGACGCGTACCGCACAT	1st PCR for deep sequencing of Site 13
S13-R	TCGGATCGGAAGCAGACGCAG	
S14-F	GCGGATCTAGCTGCGGTTGC	1st PCR for deep sequencing of Site 14
S14-R	TGGGACAAATGGAGACCGTGCATC	
S15-F	TGCATACGTGGTGGTGACTCACAT	1st PCR for deep sequencing of Site 15
S15-R	AGCGGTACTCAACATCCGGCG	
S16-F	TCCCACCTGAGGTGCAGCCTTC	1st PCR for deep sequencing of Site 16
S16-R	CGCTCGAGAACGTCACGAACCC	
S17-F	GTCCTCTAGACTCCCGGACCGAC	1st PCR for deep sequencing of Site 17
S17-R	GCGTGCGTGGATGGGTGGATAC	
S18-F	TGGAACAGCAGACCAGCCACGAC	1st PCR for deep sequencing of Site 18
S18-R	ACCCCTGAGGAAATTTGCTGTCAGC	
S19-F	CCAAACGACGACACGTAGCCAG	1st PCR for deep sequencing of Site 19
S19-R	GAGGTGCGTGGGTGACCTGTC	

S20-F	TACCAAACGACGACACGTAGCCAG	1st PCR for deep sequencing of Site 20
S20-R	GAGGTGCGTGGGTGACCTGTC	
S21-F	TGAGGGGGTGGACGGATAGA	1st PCR for deep sequencing of Site 21
S21-R	GATTAGTAAGAGCATTATCTGGCGA	
S22-F	AGCTGTTGAATGGGCAAGCGAC	1st PCR for deep sequencing of Site 22
S22-R	AAGTCCGTCCCGCTAACACCC	
S23-F	CCGGGACGGTGCAAGTTGGTTC	1st PCR for deep sequencing of Site 23
S23-R	TGCGTGGACGGACGTTCTCCTG	
S24-F	CGGGCATCATTTCGCGCCAG	1st PCR for deep sequencing of Site 24
S24-R	TGACGACGAAGAGCCGGTGC	
S25-F	TAATGGCCATGGTGTTACGCA	1st PCR for deep sequencing of Site 25
S25-R	ACGTGTTCCAAACACCCCAC	
S26-F	GCGATGTTCCATTCTTGCACTAC	1st PCR for deep sequencing of Site 26
S26-R	GTATAAGGACGATGCTGCACCA	
S27-F	CCTCCCAACCGATTTGTGAACAG	1st PCR for deep sequencing of Site 27
S27-R	CCATTGGTGGGATGAGTTGTTGC	
S28-F	ACCAATGGCGCCATTGCGTC	1st PCR for deep sequencing of Site 28
S28-R	TAGGTGCCCTCGTAGGCGGT	
S29-F	TGTTGGGATAGATGACTTGGATTG	1st PCR for deep sequencing of Site 29
S29-R	TTGCTATTGTCTGCCGGTGAA	
S30-F	ACGTGGTAGTGGTCGTATGGTCG	1st PCR for deep sequencing of Site 30
S30-R	CACCTTCTGCGCACCTGCTTC	
S31-F	CGGGTGTTGCAAGTCCTCCCT	1st PCR for deep sequencing of Site 31
S31-R	TGGTAGGATCCCTGACTCCTTTGGA	
S32-F	AGTCAGGGATCCTACCAAACAACCG	1st PCR for deep sequencing of Site 32

S32-R	ACCGTCGTTCAGTTGGCCCT	
S33-F	GAAAGATCGATGGTGTGCGTG	1st PCR for deep sequencing of Site 33
S33-R	TATGCACATGGTGATGATGTCGT	
S34-F	GCCGGCTCTTCCTCTCTTGCATC	1st PCR for deep sequencing of Site 34
S34-R	GCTCGAAGAAGGTGGCGATATTCG	
S35-F	CCACGCATGCAACCAATGAG	1st PCR for deep sequencing of Site 35
S35-R	CGAGGAAGAAGAGCTGGAGAAG	
S36-F	GGGTCTCTCCGGGAGGCGTG	1st PCR for deep sequencing of Site 36
S36-R	CCGTGGCGAGCACGTCGTAG	
S37-F	TCCGGTCCAACATATGCACTCATC	1st PCR for deep sequencing of Site 37
S37-R	TCTAGGCATGGCCCTCGTGC	
S38-F	AGACACATATGCTATCATGGCAC	1st PCR for deep sequencing of Site 38
S38-R	GGAACAAATGACCAAAAGGAATTG	
S39-F	CACAGTTCACAAGCGAGGGA	1st PCR for deep sequencing of Site 39
S39-R	GGCATATCCGGTCAACGAGA	
S40-F	GCGCGTTGCACGCGAATATACG	1st PCR for deep sequencing of Site 40
S40-R	TCGTCGTCGTTCGTCAGGTGAGG	
S1-A-F	GACGACCGAGATCGATCAGATCGCACG	2nd PCR for deep sequencing of Site 1 treated by Cas9 in rice protoplasts (Fig. 1b)
S1-A-R	CGATGTGGTGTGGCAGTTGTGTTCTG	
S1-B-F	TGACCACGAGATCGATCAGATCGCACG	2nd PCR for deep sequencing of Site 1 in control rice protoplasts (Fig. 1b)
S1-B-R	ACAGTGGGTGTGGCAGTTGTGTTCTG	
S2-A-F	GACGACCATGAACATCCCATCTTTCTG	2nd PCR for deep sequencing of Site 2 treated by Cas9/sgRNA (Fig. 1b; Fig. 3b; Fig. 4a) or Cas9-TV/sgRNA (Fig. 3b and Fig. 4a) or Cas9/sgRNA-dsgRNA (Fig. 4a) in rice protoplasts
S2-A-R	CGATGTCTGATTCTGGGTCAGTGGTG	
S2-B-F	TGACCACATGAACATCCCATCTTTCTG	2nd PCR for deep sequencing of Site 2 in control rice protoplasts (Fig.

S2-B-R	ACAGTGCTGATTCTGGGTCAGTGGTG	1b; Fig. 3b and Fig.4a) or treated by Cas9-TV/sgRNA-dsgRNA in rice protoplasts (Fig. 4a)
S3-A-F	GCCAATTGGGGTATCCATAATCTCCAC	2nd PCR for deep sequencing of Site 3 treated by Cas9 in rice protoplasts (Fig. 1b)
S3-A-R	CAGATCGGATGAAATAGATTCGTCGCC	
S3-B-F	CTTGTATGGGGTATCCATAATCTCCAC	2nd PCR for deep sequencing of Site 3 in control rice protoplasts (Fig. 1b)
S3-B-R	ATCACGGGATGAAATAGATTCGTCGCC	
S4-A-F	GCCAATCGTGCGTCACAGTCACAATC	2nd PCR for deep sequencing of Site 4 treaed by Cas9/sgRNA (Fig. 1b; Fig. 3b; Fig. 4a) or Cas9-TV/sgRNA (Fig. 3b and Fig. 4a) or Cas9/sgRNA-dsgRNA (Fig. 4a) in rice protoplasts
S4-A-R	CAGATCACGCTTGCTGCTCTCAGAAT	
S4-B-F	CTTGTACGTGCGTCACAGTCACAATC	2nd PCR for deep sequencing of Site 4 in control rice protoplasts (Fig. 1b; Fig. 3b and Fig.4a) or treated by Cas9-TV/sgRNA-dsgRNA in rice protoplasts (Fig. 4a)
S4-B-R	ATCACGACGCTTGCTGCTCTCAGAAT	
S5-A-F	TTAGGCCCGGGTCATCCTGCCTCTGAG	2nd PCR for deep sequencing of Site 5 treated by Cas9 in rice protoplasts (Fig. 1b)
S5-A-R	ACTTGAGCAGCCAGGGGTTGGTCGT	
S5-B-F	GATCAGCCGGGTCATCCTGCCTCTGAG	2nd PCR for deep sequencing of Site 5 in control rice protoplasts (Fig. 1b)
S5-B-R	TAGCTTGCAGCCAGGGGTTGGTCGT	
S6-A-F	TTAGGCGGTGTGGAGGTTGCAAGTGCT	2nd PCR for deep sequencing of Site 6 treaed by Cas9/sgRNA (Fig. 1b; Fig. 3b; Fig. 4a) or Cas9-TV/sgRNA (Fig. 3b and Fig. 4a) or Cas9/sgRNA-dsgRNA (Fig. 4a) in rice protoplasts
S6-A-R	ACTTGATTCAAGCGCAAGCTCAGGAAGA	
S6-B-F	GATCAGGGTGTGGAGGTTGCAAGTGCT	2nd PCR for deep sequencing of Site 6 in control rice protoplasts (Fig. 1b; Fig. 3b and Fig.4a) or treated by Cas9-TV/sgRNA-dsgRNA in rice protoplasts (Fig. 4a)
S6-B-R	TAGCTTTTCAAGCGCAAGCTCAGGAAGA	
S7-A-F	GGCTACTACTCCCCCGGTGCTACCACG	2nd PCR for deep sequencing of Site 7 treated by Cas9 in rice protoplasts (Fig. 1b)
S7-A-R	AGTCAAAAAGGCGCAACGTGCGTCAC	
S7-B-F	AGTTCCTACTCCCCCGGTGCTACCACG	2nd PCR for deep sequencing of Site 7 in control rice protoplasts (Fig. 1b)
S7-B-R	ATGTCAAAAGGCGCAACGTGCGTCAC	

S8-A-F	GGCTACGAGGGGAGTACATCGGCATG	2nd PCR for deep sequencing of Site 8 treaed by Cas9/sgRNA (Fig. 1b; Fig. 3b; Fig. 4a) or Cas9-TV/sgRNA (Fig. 3b and Fig. 4a) or Cas9/sgRNA-dsgRNA (Fig. 4a) in rice protoplasts
S8-A-R	AGTCAACGTAGGAGTACGTTACCTGGC	
S8-B-F	AGTTCCGAGGGGAGTACATCGGCATG	2nd PCR for deep sequencing of Site 8 in control rice protoplasts (Fig. 1b; Fig. 3b and Fig.4a) or treated by Cas9-TV/sgRNA-dsgRNA in rice protoplasts (Fig. 4a)
S8-B-R	ATGTCACGTAGGAGTACGTTACCTGGC	
S9-A-F	CCGTCCGGGCACACACCAAACCAGCC	2nd PCR for deep sequencing of Site 9 treated by Cas9 in rice protoplasts (Fig. 1b)
S9-A-R	GTAGAGGATGCGACGACGACGGCTGC	
S9-B-F	GTCCGCGGGCACACACCAAACCAGCC	2nd PCR for deep sequencing of Site 9 in control rice protoplasts (Fig. 1b)
S9-B-R	GTGAAAGATGCGACGACGACGGCTGC	
S10-A-F	CCGTCCCCTCGACCTCGACTTCGCGC	2nd PCR for deep sequencing of Site 10 treaed by Cas9/sgRNA (Fig. 1b; Fig. 3b; Fig. 4a) or Cas9-TV/sgRNA (Fig. 3b and Fig. 4a) or Cas9/sgRNA-dsgRNA (Fig. 4a) in rice protoplasts
S10-A-R	GTAGAGGCATCCATCTCGTCTCAGGACACTC	
S10-B-F	GTCCGCCCTCGACCTCGACTTCGCGC	2nd PCR for deep sequencing of Site 10 in control rice protoplasts (Fig. 1b; Fig. 3b and Fig.4a) or treated by Cas9-TV/sgRNA-dsgRNA in rice protoplasts (Fig. 4a)
S10-B-R	GTGAAAGCATCCATCTCGTCTCAGGACACTC	
S11-A-F	GTGGCCCCGATCCGTAAACCTCGCCCA	2nd PCR for deep sequencing of Site 11 treated by Cas9 in rice protoplasts (Fig. 1b)
S11-A-R	GTTTCGCGGTTGGATCTTTGCGGGGG	
S11-B-F	CGTACGCCGATCCGTAAACCTCGCCCA	2nd PCR for deep sequencing of Site 11 in control rice protoplasts (Fig. 1b)
S11-B-R	GAGTGGCGGTTGGATCTTTGCGGGGG	
S12-A-F	GTGGCCTTCGACATGACCGTCGGCGGAG	2nd PCR for deep sequencing of Site 12 treaed by Cas9/sgRNA (Fig. 1b; Fig. 3b; Fig. 4a) or Cas9-TV/sgRNA (Fig. 3b and Fig. 4a) or Cas9/sgRNA-dsgRNA (Fig. 4a) in rice protoplasts
S12-A-R	GTTTCGTGGAAGGTGCTCCCCTTGTAGTGC	
S12-B-F	CGTACGTTCGACATGACCGTCGGCGGAG	2nd PCR for deep sequencing of Site 12 in control rice protoplasts (Fig. 1b; Fig. 3b and Fig.4a) or treated by Cas9-TV/sgRNA-dsgRNA in rice protoplasts (Fig. 4a)
S12-B-R	GAGTGGTGGAAGGTGCTCCCCTTGTAGTGC	

S13-A-F	GGTAGCGCGTACCGCACATTTCCGGGG	2nd PCR for deep sequencing of Site 13 treated by Cas9 (Fig. 1b) in rice protoplasts
S13-A-R	ACTGATGGGCGGAGAGGGAGAGGAGATAG	
S13-B-F	ATGAGCGCGTACCGCACATTTCCGGGG	2nd PCR for deep sequencing of Site 13 in control rice protoplasts (Fig. 1b)
S13-B-R	ATTCCTGGGCGGAGAGGGAGAGGAGATAG	
S14-A-F	GGTAGCGAGCGGAGATGGGGCTCACG	2nd PCR for deep sequencing of Site 14 treaed by Cas9/sgRNA (Fig. 1b; Fig. 3b; Fig. 4a) or Cas9-TV/sgRNA (Fig. 3b and Fig. 4a) or Cas9/sgRNA-dsgRNA (Fig. 4a) in rice protoplasts
S14-A-R	ACTGATGTGGATTGGAAACGCACCGATGG	
S14-B-F	ATGAGCGAGCGGAGATGGGGCTCACG	2nd PCR for deep sequencing of Site 14 in control rice protoplasts (Fig. 1b; Fig. 3b and Fig.4a) or treated by Cas9-TV/sgRNA-dsgRNA in rice protoplasts (Fig. 4a)
S14-B-R	ATTCCTGTGGATTGGAAACGCACCGATGG	
S15-A-F	CAAAAGCACGTCATGTAGCAAAAGGGCGG	2nd PCR for deep sequencing of Site 15 treated by Cas9 in rice protoplasts (Fig. 1b)
S15-A-R	CAACTAGGGATTTATAGCCGGCTCAGGGG	
S15-B-F	CACCGGCACGTCATGTAGCAAAAGGGCGG	2nd PCR for deep sequencing of Site 15 in control rice protoplasts (Fig. 1b)
S15-B-R	CACGATGGGATTTATAGCCGGCTCAGGGG	
S16-A-F	CAAAAGTTCGTGGGGTTGGTGGGAAA	2nd PCR for deep sequencing of Site 16 treaed by Cas9/sgRNA (Fig. 1b; Fig. 3b; Fig. 4a) or Cas9-TV/sgRNA (Fig. 3b and Fig. 4a) or Cas9/sgRNA-dsgRNA (Fig. 4a) in rice protoplasts
S16-A-R	CAACTAGGAATCAATCCATCGGACCTTGG	
S16-B-F	CACCGGTTCGTGGGGTTGGTGGGAAA	2nd PCR for deep sequencing of Site 16 in control rice protoplasts (Fig. 1b; Fig. 3b and Fig.4a) or treated by Cas9-TV/sgRNA-dsgRNA in rice protoplasts (Fig. 4a)
S16-B-R	CACGATGGAATCAATCCATCGGACCTTGG	
S17-A-F	CACTCAGTCCAACCCAACCCCCACCG	2nd PCR for deep sequencing of Site 17 treated by Cas9 in rice protoplasts (Fig. 1b)
S17-A-R	CAGGCGAAGGGGAGCGTTGGGTTGGG	
S17-B-F	CATGGCGTCCAACCCAACCCCCACCG	2nd PCR for deep sequencing of Site 17 in control rice protoplasts (Fig. 1b)
S17-B-R	CATTTTAAGGGGAGCGTTGGGTTGGG	
S18-A-F	CACTCACACCTAGCCGGGTGGCACAC	2nd PCR for deep sequencing of Site 18 treaed by Cas9/sgRNA (Fig. 1b; Fig. 3b; Fig. 4a) or Cas9-TV/sgRNA (Fig. 3b and Fig. 4a) or
S18-A-R	CAGGCGGCCGTTCTCACCCACAGCATCC	

		Cas9/sgRNA-dsgRNA (Fig. 4a) in rice protoplasts
S18-B-F	CATGGCCACCTAGCCGGGTGGCACAC	2nd PCR for deep sequencing of Site 18 in control rice protoplasts (Fig. 1b; Fig. 3b and Fig.4a) or treated by Cas9-TV/sgRNA-dsgRNA in rice protoplasts (Fig. 4a)
S18-B-R	CATTTTGCCGTTCTCACCCACAGCATCC	
S19-A-F	CCAACACTTGGGCGACAAACCACAACCTG	2nd PCR for deep sequencing of Site 19 treated by Cas9 in rice protoplasts (Fig. 1b)
S19-A-R	CGGAATTTTCTCCAGAAGGCGCAGCCG	
S19-B-F	CTAGCTCTTGGGCGACAAACCACAACCTG	2nd PCR for deep sequencing of Site 19 in control rice protoplasts (Fig. 1b)
S19-B-R	CTATACTTTCTCCAGAAGGCGCAGCCG	
S20-A-F	CCAACAGGTAGCGAGCGTGGAACTGATG	2nd PCR for deep sequencing of Site 20 treaed by Cas9/sgRNA (Fig. 1b; Fig. 3b; Fig. 4a) or Cas9-TV/sgRNA (Fig. 3b and Fig. 4a) or Cas9/sgRNA-dsgRNA (Fig. 4a) in rice protoplasts
S20-A-R	CGGAATCTTGCTTGTATGCAATCCCATGGG	
S20-B-F	CTAGCTGGTAGCGAGCGTGGAACTGATG	2nd PCR for deep sequencing of Site 20 in control rice protoplasts (Fig. 1b; Fig. 3b and Fig.4a) or treated by Cas9-TV/sgRNA-dsgRNA in rice protoplasts (Fig. 4a)
S20-B-R	CTATACCTTGCTTGTATGCAATCCCATGGG	
S21-A-F	CTCAGAAGACGGACGTAAGTACGTAC	2nd PCR for deep sequencing of Site 21 treated by Cas9 in rice protoplasts (Fig. 1b)
S21-A-R	GCGCTAAGCAAAACATCCCACCCTTA	
S21-B-F	TAATCGAGACGGACGTAAGTACGTAC	2nd PCR for deep sequencing of Site 21 in control rice protoplasts (Fig. 1b)
S21-B-R	TACAGCAGCAAAACATCCCACCCTTA	
S22-A-F	CTCAGATGTTGAATGGGCAAGCGAC	2nd PCR for deep sequencing of Site 22 treaed by Cas9/sgRNA (Fig. 1b; Fig. 3b; Fig. 4a) or Cas9-TV/sgRNA (Fig. 3b and Fig. 4a) or Cas9/sgRNA-dsgRNA (Fig. 4a) in rice protoplasts
S22-A-R	GCGCTAAATAAAATCTAAAACCGGGGAAC	
S22-B-F	TAATCGTGTTGAATGGGCAAGCGAC	2nd PCR for deep sequencing of Site 22 in control rice protoplasts (Fig. 1b; Fig. 3b and Fig.4a) or treated by Cas9-TV/sgRNA-dsgRNA in rice protoplasts (Fig. 4a)
S22-B-R	TACAGCAATAAAATCTAAAACCGGGGAAC	
S22-C-F	CTGCAGTGTTGAATGGGCAAGCGAC	2nd PCR for deep sequencing of Site 22 treaed by Cas9/sgRNA in rice protoplasts (Fig. 1b)
S22-C-R	GAAGTTAATAAAATCTAAAACCGGGGAAC	

S23-A-F	TATAATCCGGTCGATCCATCCATCCATCC	2nd PCR for deep sequencing of Site 23 treated by Cas9 in rice protoplasts (Fig. 1b)
S23-A-R	TCATTCGCAAGTCGCTGGGAAAGCGG	
S23-B-F	TCCCGACCGGTCGATCCATCCATCCATCC	2nd PCR for deep sequencing of Site 23 in control rice protoplasts (Fig. 1b)
S23-B-R	TCGAAGGCAAGTCGCTGGGAAAGCGG	
S24-A-F	TATAATTAGTGACCTGCAGCGCCTGCAG	2nd PCR for deep sequencing of Site 24 treaed by Cas9/sgRNA (Fig. 1b; Fig. 3b; Fig. 4a; Fig. 7) or Cas9-TV/sgRNA (Fig. 3b; Fig. 4a and Fig. 7) or Cas9/sgRNA-dsgRNA (Fig. 4a) in rice protoplasts
S24-A-R	TCATTCCGGTGTCGAACGTGCCAAGC	
S24-B-F	TCCCGATAGTGACCTGCAGCGCCTGCAG	2nd PCR for deep sequencing of Site 24 in control rice protoplasts (Fig. 1b; Fig. 3b and Fig.4a) or treated by Cas9-TV/sgRNA-dsgRNA in rice protoplasts (Fig. 4a and Fig. 7)
S24-B-R	TCGAAGCGGTGTCGAACGTGCCAAGC	
S24-C-F	GCACCCTAGTGACCTGCAGCGCCTGCAG	2nd PCR for deep sequencing of Site 24 treaed by Cas9/sgRNA in rice protoplasts (Fig. 1b)
S24-C-R	GCAGGACGGTGTCGAACGTGCCAAGC	
S25-A-F	TCGGCAGGTGTTACGCAATGACTAAAGTTG	2nd PCR for deep sequencing of Site 25 treated by Cas9 in rice protoplasts (Fig. 1b)
S25-A-R	AAACACCTGATCTTTTCTATAGCTAACCTAGC	
S25-B-F	TGAAGGGGTGTTACGCAATGACTAAAGTTG	2nd PCR for deep sequencing of Site 25 in control rice protoplasts (Fig. 1b)
S25-B-R	AACATACTGATCTTTTCTATAGCTAACCTAGC	
S26-A-F	TCGGCAGAACAAAGCGATCTTTTCTCG	2nd PCR for deep sequencing of Site 26 treaed by Cas9/sgRNA (Fig. 1b; Fig. 3b; Fig. 4a) or Cas9-TV/sgRNA (Fig. 3b and Fig. 4a) or Cas9/sgRNA-dsgRNA (Fig. 4a) in rice protoplasts
S26-A-R	AAACACCAGTACTCATCGGTGTGTAG	
S26-B-F	TGAAGGGAACAAAGCGATCTTTTCTCG	2nd PCR for deep sequencing of Site 26 in control rice protoplasts (Fig. 1b; Fig. 3b and Fig.4a) or treated by Cas9-TV/sgRNA-dsgRNA in rice protoplasts (Fig. 4a)
S26-B-R	AACATACAGTACTCATCGGTGTGTAG	
S26-C-F	GCCGCGGAACAAAGCGATCTTTTCTCG	2nd PCR for deep sequencing of Site 26 treaed by Cas9/sgRNA in rice protoplasts (Fig. 1b)
S26-C-R	GGCGGTCAGTACTCATCGGTGTGTAG	
S27-A-F	CGCGTCTGAGTTGCCCGTCACTGGTATTC	2nd PCR for deep sequencing of Site 27 treated by Cas9 in rice protoplasts (Fig. 1b)
S27-A-R	GATACAGCCAGATCCTCACACCTCTACC	

S27-B-F	GGTGTGTGAGTTGCCCGTCACTGGTATTC	2nd PCR for deep sequencing of Site 27 in control rice protoplasts (Fig. 1b)
S27-B-R	TAAGAAGCCAGATCCTCACACCTCTACC	
S28-A-F	CGCGTCCCTGCAGCAAGAGCATCGTCTTC	2nd PCR for deep sequencing of Site 28 treaed by Cas9/sgRNA (Fig. 1b; Fig. 3b; Fig. 4a; Fig. 7) or Cas9-TV/sgRNA (Fig. 3b; Fig. 4a and Fig. 7) or Cas9/sgRNA-dsgRNA (Fig. 4a) in rice protoplasts
S28-A-R	GATACACACGTTGGCCTGGAACGTGC	
S28-B-F	GGTGTGCCTGCAGCAAGAGCATCGTCTTC	2nd PCR for deep sequencing of Site 28 in control rice protoplasts (Fig. 1b; Fig. 3b and Fig.4a) or treated by Cas9-TV/sgRNA-dsgRNA in rice protoplasts (Fig. 4a and Fig. 7)
S28-B-R	TAAGAACACGTTGGCCTGGAACGTGC	
S28-C-F	GTATTACCTGCAGCAAGAGCATCGTCTTC	2nd PCR for deep sequencing of Site 29 treaed by Cas9/sgRNA in rice protoplasts (Fig. 1b)
S28-C-R	TACGTGCACGTTGGCCTGGAACGTGC	
S29-A-F	AGCGAGGTAGGGAGAATGAAAAGCACAGTGA	2nd PCR for deep sequencing of Site 29 treated by Cas9 in rice protoplasts (Fig. 1b)
S29-A-R	CGGTTACTGCCACTACTCGTCTTTCCC	
S29-B-F	AGCTTTGTAGGGAGAATGAAAAGCACAGTGA	2nd PCR for deep sequencing of Site 29 in control rice protoplasts (Fig. 1b)
S29-B-R	TGGTCTCTGCCACTACTCGTCTTTCCC	
S30-A-F	AGCGAGTGCTGCTGGCGATCTACAAC	2nd PCR for deep sequencing of Site 30 treaed by Cas9/sgRNA (Fig. 1b; Fig. 3b; Fig. 4a) or Cas9-TV/sgRNA (Fig. 3b and Fig. 4a) or Cas9/sgRNA-dsgRNA (Fig. 4a) in rice protoplasts
S30-A-R	CGGTTACTTCTTGGGGGACTTGGTGG	
S30-B-F	AGCTTTTGCTGCTGGCGATCTACAAC	2nd PCR for deep sequencing of Site 30 in control rice protoplasts (Fig. 1b; Fig. 3b and Fig.4a) or treated by Cas9-TV/sgRNA-dsgRNA in rice protoplasts (Fig. 4a)
S30-B-R	TGGTCTCTTCTTGGGGGACTTGGTGG	
S30-C-F	TCACATTGCTGCTGGCGATCTACAAC	2nd PCR for deep sequencing of Site 30 treaed by Cas9/sgRNA in rice protoplasts (Fig. 1b)
S30-C-R	TCTATACTTCTTGGGGGACTTGGTGG	
S31-A-F	TATCCCGTATGTCGATGCAAAGGGAAGC	2nd PCR for deep sequencing of Site 31 treated by Cas9 in rice protoplasts (Fig. 1b)
S31-A-R	TGTCGTCACGCAACTCATCTCGTTGG	
S31-B-F	CCCCACGTATGTCGATGCAAAGGGAAGC	2nd PCR for deep sequencing of Site 31 in control rice protoplasts (Fig. 1b)
S31-B-R	ATACGACACGCAACTCATCTCGTTGG	

S32-A-F	TATCCCTGCATGTACATGATGATGAGG	2nd PCR for deep sequencing of Site 32 treaed by Cas9/sgRNA (Fig. 1b; Fig. 3b; Fig. 4a) or Cas9-TV/sgRNA (Fig. 3b and Fig. 4a) or Cas9/sgRNA-dsgRNA (Fig. 4a) in rice protoplasts
S32-A-R	TGTCGTGCCCTTGAAAACTTTTCCAAAG	
S32-B-F	CCCCACTGCATGTACATGATGATGAGG	2nd PCR for deep sequencing of Site 32 in control rice protoplasts (Fig. 1b; Fig. 3b and Fig.4a) or treated by Cas9-TV/sgRNA-dsgRNA in rice protoplasts (Fig. 4a)
S32-B-R	ATACGAGCCCTTGAAAACTTTTCCAAAG	
S32-C-F	TGCAAATGCATGTACATGATGATGAGG	2nd PCR for deep sequencing of Site 32 treaed by Cas9/sgRNA in rice protoplasts (Fig. 1b)
S32-C-R	TGGCACGCCCTTGAAAACTTTTCCAAAG	
S33-A-F	CCCTTGGCAAATGTGCCACTGGAACAGA	2nd PCR for deep sequencing of Site 33 treated by Cas9 in rice protoplasts (Fig. 1b)
S33-A-R	ACCGGCCCGCTAGAACCAAACTTATTTGCC	
S33-B-F	TTACTGGCAAATGTGCCACTGGAACAGA	2nd PCR for deep sequencing of Site 33 in control rice protoplasts (Fig. 1b)
S33-B-R	GGAACTCCGCTAGAACCAAACTTATTTGCC	
S34-A-F	CCCTTGGCCGCTGCGATAGGCAGCTA	2nd PCR for deep sequencing of Site 34 treaed by Cas9/sgRNA (Fig. 1b; Fig. 3b; Fig. 4a; Fig. 7; and Fig. S7) or Cas9-TV/sgRNA (Fig. 3b; Fig. 4a; Fig. 5 and Fig. 7) or Cas9/sgRNA-dsgRNA (Fig. 4a) in rice protoplasts
S34-A-R	ACCGGCGGTGGCGATATTCGCGGGGT	
S34-B-F	TTACTGGCCGCTGCGATAGGCAGCTA	2nd PCR for deep sequencing of Site 34 in control rice protoplasts (Fig. 1b; Fig. 3b and Fig.4a) or treated by Cas9-TV/sgRNA-dsgRNA in rice protoplasts (Fig. 4a; and Fig. 7)
S34-B-R	GGAACTGGTGGCGATATTCGCGGGGT	
S34-C-F	TGTTAGGCCGCTGCGATAGGCAGCTA	2nd PCR for deep sequencing of Site 34 treaed by Cas9/sgRNA in rice protoplasts (Fig. 1b)
S34-C-R	TTCTATGGTGGCGATATTCGCGGGGT	
S35-A-F	GTTATTAACTTTTACATCTCGCAACG	2nd PCR for deep sequencing of Site 35 treated by Cas9 in rice protoplasts (Fig. 1b)
S35-A-R	AAAAGTTAAGCGCCATTGATGATCTC	
S35-B-F	AAGGGAAACTTTTACATCTCGCAACG	2nd PCR for deep sequencing of Site 35 in control rice protoplasts (Fig. 1b)
S35-B-R	AAGTATTAAGCGCCATTGATGATCTC	
S36-A-F	GTTATTGACACCCGGTGGGCTGCATC	2nd PCR for deep sequencing of Site 36 treaed by Cas9/sgRNA (Fig.

S36-A-R	AAAAGTTCGAGCACGGCGTCGCCGAAC	1b; Fig. 3b; Fig. 4a) or Cas9-TV/sgRNA (Fig. 3b and Fig. 4a) or Cas9/sgRNA-dsgRNA (Fig. 4a) in rice protoplasts
S36-B-F	AAGGGAGACACCCGGTGGGCTGCATC	2nd PCR for deep sequencing of Site 36 in control rice protoplasts (Fig. 1b; Fig. 3b and Fig.4a) or treated by Cas9-TV/sgRNA-dsgRNA in rice protoplasts (Fig. 4a)
S36-B-R	AAGTATTCGAGCACGGCGTCGCCGAAC	
S36-C-F	GGTACGGACACCCGGTGGGCTGCATC	2nd PCR for deep sequencing of Site 36 treaed by Cas9/sgRNA in rice protoplasts (Fig. 1b)
S36-C-R	GCTACGTCGAGCACGGCGTCGCCGAAC	
S37-A-F	ACATCTCCGGTCCAACATATGCACTCATCA	2nd PCR for deep sequencing of Site 37 treated by Cas9 in rice protoplasts (Fig. 1b)
S37-A-R	ACGATTTCATGGCCCATAGGTTTTTGACCCT	
S37-B-F	ACGCCGCCGGTCCAACATATGCACTCATCA	2nd PCR for deep sequencing of Site 37 in control rice protoplasts (Fig. 1b)
S37-B-R	ACTCTCTCATGGCCCATAGGTTTTTGACCCT	
S38-A-F	ACATCTGCGTGATGTAAACGGTGATTAG	2nd PCR for deep sequencing of Site 38 treaed by Cas9/sgRNA (Fig. 1b; Fig. 3b; Fig. 4a; Fig. 7) or Cas9-TV/sgRNA (Fig. 3b; Fig. 4a and Fig. 7) or Cas9/sgRNA-dsgRNA (Fig. 4a) in rice protoplasts
S38-A-R	ACGATTCCAAAGATGCACATCACAAGATG	
S38-B-F	ACGCCGGCGTGATGTAAACGGTGATTAG	2nd PCR for deep sequencing of Site 38 in control rice protoplasts (Fig. 1b; Fig. 3b and Fig.4a) or treated by Cas9-TV/sgRNA-dsgRNA in rice protoplasts (Fig. 4a and Fig. 7)
S38-B-R	ACTCTCCCAAAGATGCACATCACAAGATG	
S38-C-F	TGGAATGCGTGATGTAAACGGTGATTAG	2nd PCR for deep sequencing of Site 38 treaed by Cas9/sgRNA in rice protoplasts (Fig. 1b)
S38-C-R	CATGTGCCAAAGATGCACATCACAAGATG	
S39-A-F	AGAATCTGGCTCGTGAGTGCGCGTTG	2nd PCR for deep sequencing of Site 39 treated by Cas9 in rice protoplasts (Fig. 1b)
S39-A-R	ATTGGGTGAGCTCGTAACTGAGCAGGCG	
S39-B-F	CCGCGTTGGCTCGTGAGTGCGCGTTG	2nd PCR for deep sequencing of Site 39 in control rice protoplasts (Fig. 1b)
S39-B-R	CGCCCTTGAGCTCGTAACTGAGCAGGCG	
S40-A-F	AGAATCTACGCACGCATATATCGCCTG	2nd PCR for deep sequencing of Site 40 treaed by Cas9/sgRNA (Fig. 1b; Fig. 3b; Fig. 4a) or Cas9-TV/sgRNA (Fig. 3b and Fig. 4a) or Cas9/sgRNA-dsgRNA (Fig. 4a) in rice protoplasts
S40-A-R	ATTGGGTCGTCGTTCGTCAGGTGAGG	

S40-B-F	CCGCGTTACGCACGCATATATCGCCTG	2nd PCR for deep sequencing of Site 40 in control rice protoplasts (Fig. 1b; Fig. 3b and Fig.4a) or treated by Cas9-TV/sgRNA-dsgRNA in rice protoplasts (Fig. 4a)
S40-B-R	CGCCCTTCGTCGTTCGTCAGGTGAGG	
S40-C-F	GAGATGTACGCACGCATATATCGCCTG	2nd PCR for deep sequencing of Site 40 treaed by Cas9/sgRNA in rice protoplasts (Fig. 1b)
S40-C-R	GATACCTCGTCGTTCGTCAGGTGAGG	
A-SG-F	GGCACGCAAACGAATCGAGGAGT	Constructing vector expressing sgRNA_A
A-SG-R	AAACACTCCTCGATTCGTTTGCG	
B-SG-F	GGCATCGCCATCGCCATCGCCGC	Constructing vector expressing sgRNA_B
B-SG-R	AAACGCGGCGATGGCGATGGCGA	
C-SG-F	GGCACGAAGGCGGCGGCGGCGATC	Constructing vector expressing sgRNA_C
C-SG-R	AAACGATCGCCGCCGCCGCCTTCG	
D-SG-F	GGCATCTTCTTCTAATCTTGCTGT	Constructing vector expressing sgRNA_D
D-SG-R	AAACACAGCAAGATTAGAAGAAGA	
E-SG-F	GGCAGTTGCTGCTGCTGCTGCGA	Constructing vector expressing sgRNA_E
E-SG-R	AAACTCGCAGCAGCAGCAGCAAC	
A1-F	GGAGTAGAGGTGGGAGGAAGCG	1st PCR for deep sequencing of A1
A1-R	GGACGATTCATCCCCGATGCATG	
A2-F	GAGTCGGCGGGAATCTGGGTG	1st PCR for deep sequencing of A2
A2-R	AAAAGCGGTGGTGGACAGTCCC	
B1-F	GTAGCTCTTGCCGCACGTGTCG	1st PCR for deep sequencing of B1
B1-R	CTTCCACGCGTGCTTCGACCAG	
B2-F	TACGCGGAGCGGATATACCGGTAC	1st PCR for deep sequencing of B2
B2-R	CATGAACTTGGCGGCGACCATGAC	
C1-F	TCAGGTTTCGCGAAGGTCGCC	1st PCR for deep sequencing of C1
C1-R	GATCGGACGGCCAGAAGAGGC	

C2-F	GGACCGCAGCATCCGATCGC	1st PCR for deep sequencing of C2
C2-R	GCCGCTGATTCCACCACCGATG	
D1-F	AGCGTGGTGAATGGCCTTAGGGT	1st PCR for deep sequencing of D1
D1-R	TGGCCGCAACATCTGGGTCTC	
D2-F	CGGAACCCATCATCTGCACTC	1st PCR for deep sequencing of D2
D2-R	CTATAGTGGTGGGAGTGGCGT	
E1-F	GGCGGGTCGTTGACCTGAGA	1st PCR for deep sequencing of E1
E1-R	GCGCATTCCGTGGCCCAAGA	
E2-F	AGCTCAGCTCGGATCCGTAGGAC	1st PCR for deep sequencing of E2
E2-R	GATGGCCCAATTGCAAGTTGCAACC	
A1-A-F	GGCTACGGTGGGGTGGGGACTACCACC	2nd PCR for deep sequencing of A1 treated by Cas9 in rice protoplasts (Fig. 2a)
A1-A-R	AGTCAACGAGCGGATGAGAGGGGATCG	
A1-B-F	AGTTCCGGTGGGGTGGGGACTACCACC	2nd PCR for deep sequencing of A1 in control rice protoplasts (Fig. 2a)
A1-B-R	ATGTCACGAGCGGATGAGAGGGGATCG	
A2-A-F	CCGTCCGGGATCGATTGATTGCCAACCG	2nd PCR for deep sequencing of A2 treated by Cas9 in rice protoplasts (Fig. 2a)
A2-A-R	GTAGAGGACTGACTTGTGGTGGGGACTAC	
A2-B-F	GTCCGCGGGATCGATTGATTGCCAACCG	2nd PCR for deep sequencing of A2 in control rice protoplasts (Fig. 2a)
A2-B-R	GTGAAAGACTGACTTGTGGTGGGGACTAC	
B1-A-F	GACGACGTAGCTCTTGCCGCACGTGTCG	2nd PCR for deep sequencing of B1 treated by Cas9 in rice protoplasts (Fig. 2a)
B1-A-R	CGATGTCTTCCACGCGTGCTTCGACCAG	
B1-B-F	TGACCAGTAGCTCTTGCCGCACGTGTCG	2nd PCR for deep sequencing of B1 in control rice protoplasts (Fig. 2a)
B1-B-R	ACAGTGCTTCCACGCGTGCTTCGACCAG	
B2-A-F	GCCAATTACGCGGAGCGGATATACCGGTAC	2nd PCR for deep sequencing of B2 treated by Cas9 in rice protoplasts (Fig. 2a)
B2-A-R	CAGATCCATGAACTTGGCGGCGACCATGAC	
B2-B-F	CTTGTATACGCGGAGCGGATATACCGGTAC	2nd PCR for deep sequencing of B2 in control rice protoplasts (Fig. 2a)

B2-B-R	ATCACGCATGAACTTGGCGGCGACCATGAC	
C1-A-F	GGTAGCGTAGATCGCCGGAGACGTGCG	2nd PCR for deep sequencing of C1 treated by Cas9 in rice protoplasts (Fig. 2a)
C1-A-R	ACTGATGGCCGCGTCGTATTTCACTTGG	
C1-B-F	ATGAGCGTAGATCGCCGGAGACGTGCG	2nd PCR for deep sequencing of C1 in control rice protoplasts (Fig. 2a)
C1-B-R	ATTCCTGGCCGCGTCGTATTTCACTTGG	
C2-A-F	GTGGCCCGATGGACGAGATCTCCACCCG	2nd PCR for deep sequencing of C2 treated by Cas9 in rice protoplasts (Fig. 2a)
C2-A-R	GTTTCGTCCGCGTCGTCCCTCGTCTG	
C2-B-F	CGTACGCGATGGACGAGATCTCCACCCG	2nd PCR for deep sequencing of C2 in control rice protoplasts (Fig. 2a)
C2-B-R	GAGTGGTCCGCGTCGTCCCTCGTCTG	
D1-A-F	CTCAGAACCTTGTGCACGGGTGTGTGTG	2nd PCR for deep sequencing of D1 treated by Cas9 in rice protoplasts (Fig. 2a)
D1-A-R	GCGCTATGGTCTGCCATGATTGCTGCCT	
D1-B-F	TAATCGACCTTGTGCACGGGTGTGTGTG	2nd PCR for deep sequencing of D1 treated by Cas9 in rice protoplasts (Fig. 2a)
D1-B-R	TACAGCTGGTCTGCCATGATTGCTGCCT	
D2-A-F	CCAACATTCCACGAAACGCACCACGC	2nd PCR for deep sequencing of D2 treated by Cas9 in rice protoplasts (Fig. 2a)
D2-A-R	CGGAATTGAGTGGCATATAAGGAAACTGGCC	
D2-B-F	CTAGCTTTCCACGAAACGCACCACGC	2nd PCR for deep sequencing of D2 in control rice protoplasts (Fig. 2a)
D2-B-R	CTATACTGAGTGGCATATAAGGAAACTGGCC	
E1-A-F	TCGGCAGCGTCTTCTGCTTCTGCACCG	2nd PCR for deep sequencing of E1 treated by Cas9 in rice protoplasts (Fig. 2a)
E1-A-R	AAACACCGTGGTGCTATGAAGTGGGCCTC	
E1-B-F	TGAAGGGCGTCTTCTGCTTCTGCACCG	2nd PCR for deep sequencing of E1 in control rice protoplasts (Fig. 2a)
E1-B-R	AACATACGTGGTGCTATGAAGTGGGCCTC	
E2-A-F	TATAATGCCTTCTCTTATCACAAATGGCG	2nd PCR for deep sequencing of E2 treated by Cas9 in rice protoplasts (Fig. 2a)
E2-A-R	TCATTCGATGAAGCAATTGCATGAGCAGC	
E2-B-F	TCCCGAGCCTTCTCTTATCACAAATGGCG	2nd PCR for deep sequencing of E2 in control rice protoplasts (Fig. 2a)
E2-B-R	TCGAAGGATGAAGCAATTGCATGAGCAGC	

T7-A-F	TAATACGACTCACTATAGGACGCAAACGA	Transcript sgRNA _A *in vitro*
T7-B-F	TAATACGACTCACTATAGGATCGCCATCG	Transcript sgRNA _B *in vitro*
T7-C-F	TAATACGACTCACTATAGGCGAAGGCGGC	Transcript sgRNA _C *in vitro*
T7-D-F	TAATACGACTCACTATAGGTCTTCTTCTA	Transcript sgRNA _D *in vitro*
T7-E-F	TAATACGACTCACTATAGGTTGCTGCTGC	Transcript sgRNA _E *in vitro*
S2-MT1-BsF	ATATATGGTCTCTGGCAGCACGGCTGGTTCTGCCGTAGTT	Constructing vector expressing sgRNA-dsgRNA of Site 2
S2-MT1-F0	CAGCACGGCTGGTTCTGCCGTAGTTTTAGAGCTAGAAATAGC	
S2-MT2-R0	AACTGCCAGATGATGTCGCTTCTTGGTGCC	
S2-MT2-BsR	ATTATTGGTCTCTAAACTGCCAGATGATGTCG	
S4-MT1-BsF	ATATATGGTCTCTGGCACGAGGAACCTGGAGAGGAAGTT	Constructing vector expressing sgRNA-dsgRNA of Site 4
S4-MT1-F0	CACGAGGAACCTGGAGAGGAAGTTTTAGAGCTAGAAATAGC	
S4-MT2-R0	AACTCGTGAAGCCTGCACGCTTCTTGGTGCC	
S4-MT2-BsR	ATTATTGGTCTCTAAACTCGTGAAGCCTGCACG	
S6-MT1-BsF	ATATATGGTCTCTGGCACCTTGTCCTTGATAGCCGTGGTT	Constructing vector expressing sgRNA-dsgRNA of Site 6
S6-MT1-F0	CACCTTGTCCTTGATAGCCGTGGTTTTAGAGCTAGAAATAGC	
S6-MT2-R0	AACTGGGCATCAGGTCACGCTTCTTGGTGCC	
S6-MT2-BsR	ATTATTGGTCTCTAAACTGGGCATCAGGTCACG	
S8-MT1-BsF	ATATATGGTCTCTGGCAGGCCACGGTGGTGTGCTGCGGTT	Constructing vector expressing sgRNA-dsgRNA of Site 8
S8-MT1-F0	CAGGCCACGGTGGTGTGCTGCGGTTTTAGAGCTAGAAATAGC	
S8-MT2-R0	AACGCAAGCACCAGCGCGCTTCTTGGTGCC	
S8-MT2-BsR	ATTATTGGTCTCTAAACGCAAGCACCAGCGCG	

S10-MT1-BsF	ATATATGGTCTCTGGCACCCGAGCTCGACATCACCGGGTT	Constructing vector expressing sgRNA-dsgRNA of Site 10
S10-MT1-F0	CACCCGAGCTCGACATCACCGGGTTTTAGAGCTAGAAATAGC	
S10-MT2-R0	AACTGGAGCGCGCGAAGCGCTTCTTGGTGCC	
S10-MT2-BsR	ATTATTGGTCTCTAAACTGGAGCGCGCGAAGCG	
S12-MT1-BsF	ATATATGGTCTCTGGCATGTACGCGAAGGACGTGCCGGTT	Constructing vector expressing sgRNA-dsgRNA of Site 12
S12-MT1-F0	CATGTACGCGAAGGACGTGCCGGTTTTAGAGCTAGAAATAGC	
S12-MT2-R0	AACCTCTTGCCCACGCCGCTTCTTGGTGCC	
S12-MT2-BsR	ATTATTGGTCTCTAAACCTCTTGCCCACGCCG	
S14-MT1-BsF	ATATATGGTCTCTGGCATCTCCTTCTTGGCGAAGAGGGTT	Constructing vector expressing sgRNA-dsgRNA of Site 14
S14-MT1-F0	CATCTCCTTCTTGGCGAAGAGGGTTTTAGAGCTAGAAATAGC	
S14-MT2-R0	AACAGCTTGAGCTTGTACGCTTCTTGGTGCC	
S14-MT2-BsR	ATTATTGGTCTCTAAACAGCTTGAGCTTGTACG	
S16-MT1-BsF	ATATATGGTCTCTGGCACGAGGCCGCCGACGAAGCAGGTT	Constructing vector expressing sgRNA-dsgRNA of Site 16
S16-MT1-F0	CACGAGGCCGCCGACGAAGCAGGTTTTAGAGCTAGAAATAGC	
S16-MT2-R0	AACCGAGTCCAAGGTCCGCTTCTTGGTGCC	
S16-MT2-BsR	ATTATTGGTCTCTAAACCGAGTCCAAGGTCCG	
S18-MT1-BsF	ATATATGGTCTCTGGCAGCAGCACCGGTTCTACCAAGGTT	Constructing vector expressing sgRNA-dsgRNA of Site 18
S18-MT1-F0	CAGCAGCACCGGTTCTACCAAGGTTTTAGAGCTAGAAATAGC	
S18-MT2-R0	AACTCACCCAATCAGGTCGCTTCTTGGTGCC	
S18-MT2-BsR	ATTATTGGTCTCTAAACTCACCCAATCAGGTCG	

S20-MT1-BsF	ATATATGGTCTCTGGCAGAGTATGTCGTGGAGTCCACGTT	Constructing vector expressing sgRNA-dsgRNA of Site 20
S20-MT1-F0	CAGAGTATGTCGTGGAGTCCACGTTTTAGAGCTAGAAATAGC	
S20-MT2-R0	AACCGCTCGCTACCATACGCTTCTTGGTGCC	
S20-MT2-BsR	ATTATTGGTCTCTAAACCGCTCGCTACCATACG	
S22-MT1-BsF	ATATATGGTCTCTGGCAGGGGTGTATCGATGATGCCGTT	Constructing vector expressing sgRNA-dsgRNA of Site 22
S22-MT1-F0	CAGGGGTGTATCGATGATGCCGTTTTAGAGCTAGAAATAGC	
S22-MT2-R0	AACAGAGCCTAGCTGTTCGCTTCTTGGTGCC	
S22-MT2-BsR	ATTATTGGTCTCTAAACAGAGCCTAGCTGTTCG	
S24-MT1-BsF	ATATATGGTCTCTGGCACGGCCGCCTCCGTACGCCGGTT	Constructing vector expressing sgRNA-dsgRNA of Site 24
S24-MT1-F0	CACGGCCGCCTCCGTACGCCGGTTTTAGAGCTAGAAATAGC	
S24-MT2-R0	AACGCAGCGCCTGCAGTCGCTTCTTGGTGCC	
S24-MT2-BsR	ATTATTGGTCTCTAAACGCAGCGCCTGCAGTC	
S26-MT1-BsF	ATATATGGTCTCTGGCAGGAACAGCCTCATCCCTTGGGTT	Constructing vector expressing sgRNA-dsgRNA of Site 26
S26-MT1-F0	CAGGAACAGCCTCATCCCTTGGGTTTTAGAGCTAGAAATAGC	
S26-MT2-R0	AACACACACCGATGAGTCGCTTCTTGGTGCC	
S26-MT2-BsR	ATTATTGGTCTCTAAACACACACCGATGAGTCG	
S28-MT1-BsF	ATATATGGTCTCTGGCAGTCTTTGGACGTAGCCATGGGTT	Constructing vector expressing sgRNA-dsgRNA of Site 28
S28-MT1-F0	CAGTCTTTGGACGTAGCCATGGGTTTTAGAGCTAGAAATAGC	
S28-MT2-R0	AACCCTCGTCCATCAACGCTTCTTGGTGCC	
S28-MT2-BsR	ATTATTGGTCTCTAAACCCTCGTCCATCAACG	
S30-MT1-BsF	ATATATGGTCTCTGGCATGTGCTGCCGAACATCAACCGTT	Constructing vector expressing sgRNA-dsgRNA of Site 30

S30-MT1-F0	CATGTGCTGCCGAACATCAACCGTTTTAGAGCTAGAAATAGC	
S30-MT2-R0	AACAGGCACGTGCTGCTCGCTTCTTGGTGCC	
S30-MT2-BsR	ATTATTGGTCTCTAAACAGGCACGTGCTGCTCG	
S32-MT1-BsF	ATATATGGTCTCTGGCACCATGACATTGTCCGAGCCAGTT	Constructing vector expressing sgRNA-dsgRNA of Site 32
S32-MT1-F0	CACCATGACATTGTCCGAGCCAGTTTTAGAGCTAGAAATAGC	
S32-MT2-R0	AACTCGTTCAGTTGGCCGCTTCTTGGTGCC	
S32-MT2-BsR	ATTATTGGTCTCTAAACTCGTTCAGTTGGCCG	
S34-MT1-BsF	ATATATGGTCTCTGGCAGACATCGTCACCAAGGCGCGTT	Constructing vector expressing sgRNA-dsgRNA of Site 34
S34-MT1-F0	CAGACATCGTCACCAAGGCGCGTTTTAGAGCTAGAAATAGC	
S34-MT2-R0	AACCGAGCGACGTGGCCGCTTCTTGGTGCC	
S34-MT2-BsR	ATTATTGGTCTCTAAACCGAGCGACGTGGCC	
S36-MT1-BsF	ATATATGGTCTCTGGCACTCGCTCCCGACCAACCAGTGTT	Constructing vector expressing sgRNA-dsgRNA of Site 36
S36-MT1-F0	CACTCGCTCCCGACCAACCAGTGTTTTAGAGCTAGAAATAGC	
S36-MT2-R0	AACGTGGGCTGCATCGGCGCTTCTTGGTGCC	
S36-MT2-BsR	ATTATTGGTCTCTAAACGTGGGCTGCATCGGCG	
S38-MT1-BsF	ATATATGGTCTCTGGCATGGGTAATGGTGATATCCCAGTT	Constructing vector expressing sgRNA-dsgRNA of Site 38
S38-MT1-F0	CATGGGTAATGGTGATATCCCAGTTTTAGAGCTAGAAATAGC	
S38-MT2-R0	AACTTGGTCTAATGCGCGCTTCTTGGTGCC	
S38-MT2-BsR	ATTATTGGTCTCTAAACTTGGTCTAATGCGC	
S40-MT1-BsF	ATATATGGTCTCTGGCACGACGATGACCATGTCCACGGTT	Constructing vector expressing sgRNA-dsgRNA of Site 40

S40-MT1-F0	CACGACGATGACCATGTCCACGGTTTTAGAGCTAGAAATAGC	
S40-MT2-R0	AACTGGTTGGTCGCGCCGCTTCTTGGTGCC	
S40-MT2-BsR	ATTATTGGTCTCTAAACTGGTTGGTCGCGCCG	
S34-2-MT2-R0	AACGTCTTCCATGTAGGCGCTTCTTGGTGCC	Constructing vector expressing sgRNA-dsgRNA2 of Site 34
S34-2-MT2-BsR	ATTATTGGTCTCTAAACGTCTTCCATGTAGGCG	
S34-3-MT2-R0	AACAGGCGTTCTGTTCCGCTTCTTGGTGCC	Constructing vector expressing sgRNA-dsgRNA3 of Site 34
S34-3-MT2-BsR	ATTATTGGTCTCTAAACAGGCGTTCTGTTCCG	
S34-4-MT2-R0	AACCGCTCACTACAGCTCGCTTCTTGGTGCC	Constructing vector expressing sgRNA-dsgRNA4 of Site 34
S34-4-MT2-BsR	ATTATTGGTCTCTAAACCGCTCACTACAGCTCG	
S34-5-MT2-R0	AACTGCATGCATCGCTGCGCTTCTTGGTGCC	Constructing vector expressing sgRNA-dsgRNA5 of Site 34
S34-5-MT2-BsR	ATTATTGGTCTCTAAACTGCATGCATCGCTGCG	
S34-6-MT2-R0	AACCGTCCCCTTTCCACGCTTCTTGGTGCC	Constructing vector expressing sgRNA-dsgRNA6 of Site 34
S34-6-MT2-BsR	ATTATTGGTCTCTAAACCGTCCCCTTTCCACG	
MT0-BsR2	ATTATTGGTCTCTGCTTCTTGGTGCCGC	Constructing vector expressing paired dsgRNAs of Site 34
13-MT2-BsF	ATATATGGTCTCTAAGCGGCCACGTCGCTCGGTT	Constructing vector expressing sgRNA-dsgRNA13 of Site 34
13-MT2-F0	GGCCACGTCGCTCGGTTTTAGAGCTAGAAATAGC	
46-MT2-BsF	ATATATGGTCTCTAAGCGTGGAAAGGGGACGGTT	Constructing vector expressing sgRNA-dsgRNA46 of Site 34
46-MT2-F0	GTGGAAAGGGGACGGTTTTAGAGCTAGAAATAGC	
25-MT2-BsF	ATATATGGTCTCTAAGCGCCTACATGGAAGACGTT	Constructing vector expressing sgRNA-dsgRNA25 of Site 34
25-MT2-F0	GCCTACATGGAAGACGTTTTAGAGCTAGAAATAGC	
S34-D-F	GTGGCCGCCGCTGCGATAGGCAGCTA	2nd PCR for deep sequencing of Site 34 treated by Cas9/sgRNA-dsgRNA1 in rice protoplasts (Fig. S7)
S34-D-R	GTTTCGGGTGGCGATATTCGCGGGGT	
S34-E-F	CGTACGGCCGCTGCGATAGGCAGCTA	2nd PCR for deep sequencing of Site 34 treated by Cas9/sgRNA-dsgRNA2 in rice protoplasts (Fig. S7)
S34-E-R	GAGTGGGGTGGCGATATTCGCGGGGT	

S34-F-F	GGTAGCGCCGCTGCGATAGGCAGCTA	2nd PCR for deep sequencing of Site 34 treated by Cas9/sgRNA-dsgRNA3 in rice protoplasts (Fig. S7)
S34-F-R	ACTGATGGTGGCGATATTCGCGGGGT	
S34-G-F	ATGAGCGCCGCTGCGATAGGCAGCTA	2nd PCR for deep sequencing of Site 34 treated by Cas9/sgRNA-dsgRNA4 in rice protoplasts (Fig. S7)
S34-G-R	ATTCCTGGTGGCGATATTCGCGGGGT	
S34-H-F	CAAAAGGCCGCTGCGATAGGCAGCTA	2nd PCR for deep sequencing of Site 34 treated by Cas9/sgRNA-dsgRNA5 in rice protoplasts (Fig. S7)
S34-H-R	CAACTAGGTGGCGATATTCGCGGGGT	
S34-I-F	CACCGGGCCGCTGCGATAGGCAGCTA	2nd PCR for deep sequencing of Site 34 treated by Cas9/sgRNA-dsgRNA1&3 in rice protoplasts (Fig. S7)
S34-I-R	CACGATGGTGGCGATATTCGCGGGGT	
S34-J-F	CACTCAGCCGCTGCGATAGGCAGCTA	2nd PCR for deep sequencing of Site 34 treated by Cas9/sgRNA-dsgRNA4&6 in rice protoplasts (Fig. S7)
S34-J-R	CAGGCGGGTGGCGATATTCGCGGGGT	
S34-K-F	CATGGCGCCGCTGCGATAGGCAGCTA	2nd PCR for deep sequencing of Site 34 treated by Cas9/sgRNA-dsgRNA2&5 in rice protoplasts (Fig. S7)
S34-K-R	CATTTTGGTGGCGATATTCGCGGGGT	
S34-L-F	CCAACAGCCGCTGCGATAGGCAGCTA	2nd PCR for deep sequencing of Site 34 treated by Cas9-TV/sgRNA-dsgRNA1 in rice protoplasts (Fig. 5)
S34-L-R	CGGAATGGTGGCGATATTCGCGGGGT	
S34-M-F	CTAGCTGCCGCTGCGATAGGCAGCTA	2nd PCR for deep sequencing of Site 34 treated by Cas9-TV/sgRNA-dsgRNA2 in rice protoplasts (Fig. 5)
S34-M-R	CTATACGGTGGCGATATTCGCGGGGT	
S34-N-F	TCGGCAGCCGCTGCGATAGGCAGCTA	2nd PCR for deep sequencing of Site 34 treated by Cas9-TV/sgRNA-dsgRNA3 in rice protoplasts (Fig. 5)
S34-N-R	AAACACGGTGGCGATATTCGCGGGGT	
S34-O-F	TGAAGGGCCGCTGCGATAGGCAGCTA	2nd PCR for deep sequencing of Site 34 treated by Cas9-TV/sgRNA-dsgRNA4 in rice protoplasts (Fig. 5)
S34-O-R	AACATAGGTGGCGATATTCGCGGGGT	
S34-P-F	CGCGTCGCCGCTGCGATAGGCAGCTA	2nd PCR for deep sequencing of Site 34 treated by Cas9-TV/sgRNA-dsgRNA5 in rice protoplasts (Fig. 5)
S34-P-R	GATACAGGTGGCGATATTCGCGGGGT	
S34-Q-F	GGTGTGGCCGCTGCGATAGGCAGCTA	2nd PCR for deep sequencing of Site 34 treated by Cas9-TV/sgRNA-dsgRNA1&3 in rice protoplasts (Fig. 5)
S34-Q-R	TAAGAAGGTGGCGATATTCGCGGGGT	
S34-R-F	AGCGAGGCCGCTGCGATAGGCAGCTA	2nd PCR for deep sequencing of Site 34 treated by Cas9-TV/sgRNA-

S34-R-R	CGGTTAGGTGGCGATATTCGCGGGGT	dsgRNA4&6 in rice protoplasts (Fig. 5)
S34-S-F	AGCTTTGCCGCTGCGATAGGCAGCTA	2nd PCR for deep sequencing of Site 34 treated by Cas9-TV/sgRNA-dsgRNA2&5 in rice protoplasts (Fig. 5)
S34-S-R	TGGTCTGGTGGCGATATTCGCGGGGT	
S34-T-F	AGAATCGCCGCTGCGATAGGCAGCTA	2nd PCR for deep sequencing of Site 34 treated by Cas9/sgRNA-dsgRNA6 in rice protoplasts (Fig. S7)
S34-T-R	ATTGGGGGTGGCGATATTCGCGGGGT	
S34-U-F	CCGCGTGCCGCTGCGATAGGCAGCTA	2nd PCR for deep sequencing of Site 34 treated by Cas9-TV/sgRNA-dsgRNA6 in rice protoplasts (Fig. 5)
S34-U-R	CGCCCTGGTGGCGATATTCGCGGGGT	
S34-V-F	GTATTAGCCGCTGCGATAGGCAGCTA	2nd PCR for deep sequencing of Site 34 treated by Cas9/sgRNA in rice protoplasts (Fig. 5)
S34-V-R	TACGTGGGTGGCGATATTCGCGGGGT	
OT24-1-F	TCCAGCCGCCACATGCACAA	1st PCR for deep sequencing of OT24-1
OT24-1-R	GGCGCCCTTCATGGCGAATG	
OT24-2-F	GCGCAACTCACAGCAGAGGGA	1st PCR for deep sequencing of OT24-2
OT24-2-R	TTGCCTTCGAGAACCCGGACC	
OT24-3-F	CGCACGACGAGCTCCAAGAGA	1st PCR for deep sequencing of OT24-3
OT24-3-R	TCACCTACGCTCATTCATCCACA	
OT28-1-F	TAAACGTCGGTCTCGCGCTCTT	1st PCR for deep sequencing of OT28-1
OT28-1-R	GCCGTATCCGCCTGCACAAAG	
OT28-2-F	TCTGCTGCAGGCCGAGATTGAA	1st PCR for deep sequencing of OT28-2
OT28-2-R	TCTGATCTCCCTGCCAACGCC	
OT28-3-F	CACGCGATGATCGACAGAGA	1st PCR for deep sequencing of OT28-3
OT28-3-R	AAGGAAGCCACTGAAGACCG	
OT34-1-F	CGATGCGCTTCACCTCCGAG	1st PCR for deep sequencing of OT34-1
OT34-1-R	AGTCGGCAACCTTCGGGGTG	
OT34-2-F	ATCATGGCGTACTACGCCTGGAG	1st PCR for deep sequencing of OT34-2
OT34-2-R	TGCTAAAGTGGGAGGGTGTGCG	

OT34-3-F	ACGCGACAGGGATCGTCCAC	1st PCR for deep sequencing of OT34-3
OT34-3-R	CGGAGGTGGCGACGCAGAC	
OT34-4-F	TCACCGCCTACGACATCACGAC	1st PCR for deep sequencing of OT34-4
OT34-4-R	TCAAGGTCATGCGATGCAACCAC	
OT34-5-F	GTGCTGCAGGTACGACCCCTC	1st PCR for deep sequencing of OT34-5
OT34-5-R	CTTGGAAGGTGAACTTCTGCCCAAC	
OT38-1-F	CCAAGCTAGCAACAACAAGCTC	1st PCR for deep sequencing of OT38-1
OT38-1-R	GTCGTCGCTAGCTATGCGTATC	
OT38-2-F	TGTGCTTGTGTTAAAGGGACACACT	1st PCR for deep sequencing of OT38-2
OT38-2-R	GTGTCTCGGTGTCGTCATCTTG	
OT38-3-F	CTCGACGACCCTCAGCTCGC	1st PCR for deep sequencing of OT38-3
OT38-3-R	GTGCAACGGTCTCCCCCTCC	
OT38-4-F	GCGGATGCCAGCTTTGAGTTC	1st PCR for deep sequencing of OT38-4
OT38-4-R	GGAGCTTTGAGTATGGCACAGC	
OT24-1-F1	GACGACCGTCCGCATGGAGGACGACA	2nd PCR for deep sequencing of OT24-1 for sgRNA24 treated by Cas9 in rice protoplasts (Fig. 7)
OT24-1-R1	CGATGTCCTTCATGGCGAATGCGGCG	
OT24-1-F2	TGACCACGTCCGCATGGAGGACGACA	2nd PCR for deep sequencing of OT24-1 for sgRNA24 treated by Cas9-TV/sgRNA in rice protoplasts (Fig. 7)
OT24-1-R2	ACAGTGCCTTCATGGCGAATGCGGCG	
OT24-1-F3	GCCAATCGTCCGCATGGAGGACGACA	2nd PCR for deep sequencing of OT24-1 for sgRNA 24 in rice protoplasts treated by Cas9-TV/sgRNA-dsgRNA (Fig. 7)
OT24-1-R3	CAGATCCCTTCATGGCGAATGCGGCG	
OT24-1-F4	CTTGTACGTCCGCATGGAGGACGACA	2nd PCR for deep sequencing of OT24-1 for sgRNA 24 in control rice protoplasts (Fig. 7)
OT24-1-R4	ATCACGCCTTCATGGCGAATGCGGCG	
OT24-2-F1	TTAGGCGCGCAACTCACAGCAGAGGGA	2nd PCR for deep sequencing of OT24-2 for sgRNA 24 treated by Cas9 in rice protoplasts (Fig. 7)
OT24-2-R1	ACTTGATTGCCTTCGAGAACCCGGACC	
OT24-2-F2	GATCAGGCGCAACTCACAGCAGAGGGA	2nd PCR for deep sequencing of OT24-2 for sgRNA 24 treated by

OT24-2-R2	TAGCTTTTGCCTTCGAGAACCCGGACC	Cas9-TV/sgRNA in rice protoplasts (Fig. 7)
OT24-2-F3	GGCTACGCGCAACTCACAGCAGAGGGA	2nd PCR for deep sequencing of OT24-2 for sgRNA 24 treated by Cas9-TV/sgRNA-dsgRNA in rice protoplasts (Fig. 7)
OT24-2-R3	AGTCAATTGCCTTCGAGAACCCGGACC	
OT24-2-F4	AGTTCCGCGCAACTCACAGCAGAGGGA	2nd PCR for deep sequencing of OT24-2 for sgRNA 24 in control rice protoplasts (Fig. 7)
OT24-2-R4	ATGTCATTGCCTTCGAGAACCCGGACC	
OT24-3-F1	CCGTCCGCACGACGAGCTCCAAGAGAG	2nd PCR for deep sequencing of OT24-3 for sgRNA 24 treated by Cas9 in rice protoplasts (Fig. 7)
OT24-3-R1	GTAGAGGCAGCCTCTGCTCCGAGTC	
OT24-3-F2	GTCCGCGCACGACGAGCTCCAAGAGAG	2nd PCR for deep sequencing of OT24-3 for sgRNA 24 treated by Cas9-TV/sgRNA in rice protoplasts (Fig. 7)
OT24-3-R2	GTGAAAGCAGCCTCTGCTCCGAGTC	
OT24-3-F3	GTGGCCGCACGACGAGCTCCAAGAGAG	2nd PCR for deep sequencing of OT24-3 for sgRNA 24 treated by Cas9-TV/sgRNA-dsgRNA in rice protoplasts (Fig. 7)
OT24-3-R3	GTTTCGGCAGCCTCTGCTCCGAGTC	
OT24-3-F4	CGTACGGCACGACGAGCTCCAAGAGAG	2nd PCR for deep sequencing of OT24-3 for sgRNA 24 in control rice protoplasts (Fig. 7)
OT24-3-R4	GAGTGGGCAGCCTCTGCTCCGAGTC	
OT28-1-F1	GGTAGCACCGATTATAGCTGACCGGCG	2nd PCR for deep sequencing of OT28-1 for sgRNA 28 treated by Cas9 in rice protoplasts (Fig. 7)
OT28-1-R1	ACTGATTCAACGCAAGGGCGTATATCA	
OT28-1-F2	ATGAGCACCGATTATAGCTGACCGGCG	2nd PCR for deep sequencing of OT28-1 for sgRNA 28 treated by Cas9-TV/sgRNA in rice protoplasts (Fig. 7)
OT28-1-R2	ATTCCTTCAACGCAAGGGCGTATATCA	
OT28-1-F3	CAAAAGACCGATTATAGCTGACCGGCG	2nd PCR for deep sequencing of OT28-1 for sgRNA 28 treated by Cas9-TV/sgRNA-dsgRNA in rice protoplasts (Fig. 7)
OT28-1-R3	CAACTATCAACGCAAGGGCGTATATCA	
OT28-1-F4	CACCGGACCGATTATAGCTGACCGGCG	2nd PCR for deep sequencing of OT28-1 for sgRNA 28 in control rice protoplasts (Fig. 7)
OT28-1-R4	CACGATTCAACGCAAGGGCGTATATCA	
OT28-2-F1	CACTCACTGCAGGCCGAGATTGAAAGGT	2nd PCR for deep sequencing of OT28-2 for sgRNA 28 treated by Cas9 in rice protoplasts (Fig. 7)
OT28-2-R1	CAGGCGTGCCAACGCCTTTTCCTTGCTTG	
OT28-2-F2	CATGGCCTGCAGGCCGAGATTGAAAGGT	2nd PCR for deep sequencing of OT28-2 for sgRNA 28 treated by Cas9-TV/sgRNA in rice protoplasts (Fig. 7)
OT28-2-R2	CATTTTTGCCAACGCCTTTTCCTTGCTTG	

OT28-2-F3	CCAACACTGCAGGCCGAGATTGAAAGGT	2nd PCR for deep sequencing of OT28-2 for sgRNA 28 treated by Cas9-TV/sgRNA-dsgRNA in rice protoplasts (Fig. 7)
OT28-2-R3	CGGAATTGCCAACGCCTTTTCCTTGCTTG	
OT28-2-F4	CTAGCTCTGCAGGCCGAGATTGAAAGGT	2nd PCR for deep sequencing of OT28-2 for sgRNA 28 in control rice protoplasts (Fig. 7)
OT28-2-R4	CTATACTGCCAACGCCTTTTCCTTGCTTG	
OT28-3-F1	TCGGCACACGCGATGATCGACAGAGA	2nd PCR for deep sequencing of OT28-3 for sgRNA 28 treated by Cas9 in rice protoplasts (Fig. 7)
OT28-3-R1	AAACACAAGGAAGCCACTGAAGACCG	
OT28-3-F2	TGAAGGCACGCGATGATCGACAGAGA	2nd PCR for deep sequencing of OT28-3 for sgRNA 28 treated by Cas9-TV/sgRNA in rice protoplasts (Fig. 7)
OT28-3-R2	AACATAAAGGAAGCCACTGAAGACCG	
OT28-3-F3	CGCGTCCACGCGATGATCGACAGAGA	2nd PCR for deep sequencing of OT28-3 for sgRNA 28 treated by Cas9-TV/sgRNA-dsgRNA in rice protoplasts (Fig. 7)
OT28-3-R3	GATACAAAGGAAGCCACTGAAGACCG	
OT28-3-F4	GGTGTGCACGCGATGATCGACAGAGA	2nd PCR for deep sequencing of OT28-3 for sgRNA 28 in control rice protoplasts (Fig. 7)
OT28-3-R4	TAAGAAAAGGAAGCCACTGAAGACCG	
OT34-1-F1	AGAATCTGAAGGTGCTCAAGGTGTCC	2nd PCR for deep sequencing of OT34-1 for sgRNA 34 treated by Cas9 in rice protoplasts (Fig. 7)
OT34-1-R1	ATTGGGGAGGTACTTCTCCAGCGAGC	
OT34-1-F2	CCGCGTTGAAGGTGCTCAAGGTGTCC	2nd PCR for deep sequencing of OT34-1 for sgRNA 34 treated by Cas9-TV/sgRNA in rice protoplasts (Fig. 7)
OT34-1-R2	CGCCCTGAGGTACTTCTCCAGCGAGC	
OT34-1-F3	CTGCAGTGAAGGTGCTCAAGGTGTCC	2nd PCR for deep sequencing of OT34-1 for sgRNA 34 treated by Cas9-TV/sgRNA-dsgRNA in rice protoplasts (Fig. 7)
OT34-1-R3	GAAGTTGAGGTACTTCTCCAGCGAGC	
OT34-1-F4	GCCGCGTGAAGGTGCTCAAGGTGTCC	2nd PCR for deep sequencing of OT34-1 for sgRNA 34 in control rice protoplasts (Fig. 7)
OT34-1-R4	GGCGGTGAGGTACTTCTCCAGCGAGC	
OT34-2-F1	GTATTATACTACGCCTGGAGCCATTA	2nd PCR for deep sequencing of OT34-2 for sgRNA 34 treated by Cas9 in rice protoplasts (Fig. 7)
OT34-2-R1	TACGTGGGCTACATAGGAGGTGACTGT	
OT34-2-F2	TCACATTACTACGCCTGGAGCCATTA	2nd PCR for deep sequencing of OT34-2 for sgRNA 34 treated by Cas9-TV/sgRNA in rice protoplasts (Fig. 7)
OT34-2-R2	TCTATAGGCTACATAGGAGGTGACTGT	
OT34-2-F3	TGCAAATACTACGCCTGGAGCCATTA	2nd PCR for deep sequencing of OT34-2 for sgRNA 34 treated by

OT34-2-R3	TGGCACGGCTACATAGGAGGTGACTGT	Cas9-TV/sgRNA-dsgRNA in rice protoplasts (Fig. 7)
OT34-2-F4	GGTACGTACTACGCCTGGAGCCATTA	2nd PCR for deep sequencing of OT34-2 for sgRNA 34 in control rice protoplasts (Fig. 7)
OT34-2-R4	GCTACGGGCTACATAGGAGGTGACTGT	
OT34-3-F1	GAGATGCGAGAGGTGCTCGACGAC	2nd PCR for deep sequencing of OT34-3 for sgRNA 34 treated by Cas9 in rice protoplasts (Fig. 7)
OT34-3-R1	GATACCCCTGGTGGATCTCCATTGAC	
OT34-3-F2	TCTAACCGAGAGGTGCTCGACGAC	2nd PCR for deep sequencing of OT34-3 for sgRNA 34 treated by Cas9-TV/sgRNA in rice protoplasts (Fig. 7)
OT34-3-R2	ACTGCACCTGGTGGATCTCCATTGAC	
OT34-3-F3	CGGATCCGAGAGGTGCTCGACGAC	2nd PCR for deep sequencing of OT34-3 for sgRNA 34 treated by Cas9-TV/sgRNA-dsgRNA in rice protoplasts (Fig. 7)
OT34-3-R3	TAGTGCCCTGGTGGATCTCCATTGAC	
OT34-3-F4	ACCAACCGAGAGGTGCTCGACGAC	2nd PCR for deep sequencing of OT34-3 for sgRNA 34 in control rice protoplasts (Fig. 7)
OT34-3-R4	GACCGGCCTGGTGGATCTCCATTGAC	
OT34-4-F1	ATCGATGTGGGCAACGCGGCGTTC	2nd PCR for deep sequencing of OT34-4 for sgRNA 34 treated by Cas9 in rice protoplasts (Fig. 7)
OT34-4-R1	CGCTCACAGCCACCTCGGCAACCTC	
OT34-4-F2	TCGGCGGTGGGCAACGCGGCGTTC	2nd PCR for deep sequencing of OT34-4 for sgRNA 34 treated by Cas9-TV/sgRNA in rice protoplasts (Fig. 7)
OT34-4-R2	ACTGGCCAGCCACCTCGGCAACCTC	
OT34-4-F3	GATTTTGTGGGCAACGCGGCGTTC	2nd PCR for deep sequencing of OT34-4 for sgRNA 34 treated by Cas9-TV/sgRNA-dsgRNA in rice protoplasts (Fig. 7)
OT34-4-R3	ACGATTCAGCCACCTCGGCAACCTC	
OT34-4-F4	ACGCCGGTGGGCAACGCGGCGTTC	2nd PCR for deep sequencing of OT34-4 for sgRNA 34 in control rice protoplasts (Fig. 7)
OT34-4-R4	ACTCTCCAGCCACCTCGGCAACCTC	
OT34-5-F1	GCACCCCCGTCGTCGGAGGTGCAAGT	2nd PCR for deep sequencing of OT34-5 for sgRNA 34 treated by Cas9 in rice protoplasts (Fig. 7)
OT34-5-R1	GCAGGATGGAAGGTGAACTTCTGCCCAACA	
OT34-5-F2	TGGAATCCGTCGTCGGAGGTGCAAGT	2nd PCR for deep sequencing of OT34-5 for sgRNA 34 treated by Cas9-TV/sgRNA in rice protoplasts (Fig. 7)
OT34-5-R2	CATGTGTGGAAGGTGAACTTCTGCCCAACA	
OT34-5-F3	GTTATTCCGTCGTCGGAGGTGCAAGT	2nd PCR for deep sequencing of OT34-5 for sgRNA 34 treated by Cas9-TV/sgRNA-dsgRNA in rice protoplasts (Fig. 7)
OT34-5-R3	AAAAGTTGGAAGGTGAACTTCTGCCCAACA	

OT34-5-F4	AAGGGACCGTCGTCGGAGGTGCAAGT	2nd PCR for deep sequencing of OT34-5 for sgRNA 34 in control rice protoplasts (Fig. 7)
OT34-5-R4	AAGTATTGGAAGGTGAACTTCTGCCCAACA	
OT38-1-F1	AGCGAGAAAAATGGCTGGCCTGTCCC	2nd PCR for deep sequencing of OT38-1 for sgRNA 38 treated by Cas9 in rice protoplasts (Fig. 7)
OT38-1-R1	CGGTTACTATGCGTATCTGCCAGCAAG	
OT38-1-F2	AGCTTTAAAAATGGCTGGCCTGTCCC	2nd PCR for deep sequencing of OT38-1 for sgRNA 38 treated by Cas9-TV/sgRNA in rice protoplasts (Fig. 7)
OT38-1-R2	TGGTCTCTATGCGTATCTGCCAGCAAG	
OT38-1-F3	TATCCCAAAAATGGCTGGCCTGTCCC	2nd PCR for deep sequencing of OT38-1 for sgRNA 38 treated by Cas9-TV/sgRNA-dsgRNA in rice protoplasts (Fig. 7)
OT38-1-R3	TGTCGTCTATGCGTATCTGCCAGCAAG	
OT38-1-F4	CCCCACAAAAATGGCTGGCCTGTCCC	2nd PCR for deep sequencing of OT38-1 for sgRNA 38 in control rice protoplasts (Fig. 7)
OT38-1-R4	ATACGACTATGCGTATCTGCCAGCAAG	
OT38-2-F1	CCCTTGCTTGTGCACTCTTGCGTGTC	2nd PCR for deep sequencing of OT38-2 for sgRNA 38 treated by Cas9 in rice protoplasts (Fig. 7)
OT38-2-R1	ACCGGCTTGATGGTTTAGCCTGGCCC	
OT38-2-F2	TTACTGCTTGTGCACTCTTGCGTGTC	2nd PCR for deep sequencing of OT38-2 for sgRNA 38 treated by Cas9-TV/sgRNA in rice protoplasts (Fig. 7)
OT38-2-R2	GGAACTTTGATGGTTTAGCCTGGCCC	
OT38-2-F3	GTTATTCTTGTGCACTCTTGCGTGTC	2nd PCR for deep sequencing of OT38-2 for sgRNA 38 treated by Cas9-TV/sgRNA-dsgRNA in rice protoplasts (Fig. 7)
OT38-2-R3	AAAAGTTTGATGGTTTAGCCTGGCCC	
OT38-2-F4	AAGGGACTTGTGCACTCTTGCGTGTC	2nd PCR for deep sequencing of OT38-2 for sgRNA 38 in control rice protoplasts (Fig. 7)
OT38-2-R4	AAGTATTTGATGGTTTAGCCTGGCCC	
OT38-3-F1	ACATCTCGCTGATTGTGATCGTTGCAT	2nd PCR for deep sequencing of OT38-3 for sgRNA 38 treated by Cas9 in rice protoplasts (Fig. 7)
OT38-3-R1	ACGATTGTTCCCCACACGAACACTGTA	
OT38-3-F2	ACGCCGCGCTGATTGTGATCGTTGCAT	2nd PCR for deep sequencing of OT38-3 for sgRNA 38 treated by Cas9-TV/sgRNA in rice protoplasts (Fig. 7)
OT38-3-R2	ACTCTCGTTCCCCACACGAACACTGTA	
OT38-3-F3	AGAATCCGCTGATTGTGATCGTTGCAT	2nd PCR for deep sequencing of OT38-3 for sgRNA 38 treaetd by Cas9-TV/sgRNA-dsgRNA in rice protoplasts (Fig. 7)
OT38-3-R3	ATTGGGGTTCCCCACACGAACACTGTA	
OT38-3-F4	CCGCGTCGCTGATTGTGATCGTTGCAT	2nd PCR for deep sequencing of OT38-3 for sgRNA 38 in control rice

OT38-3-R4	CGCCCTGTTCCCCACACGAACACTGTA	protoplasts (Fig. 7)
OT38-4-F1	CTGCAGAGCTCGCACCTTGCAGACTGT	2nd PCR for deep sequencing of OT38-4 for sgRNA 38 treated by Cas9 in rice protoplasts (Fig. 7)
OT38-4-R1	GAAGTTTGTTTTCCCGGGTGGCGGA	
OT38-4-F2	GCACCCAGCTCGCACCTTGCAGACTGT	2nd PCR for deep sequencing of OT38-4 for sgRNA 38 treated by Cas9-TV/sgRNA in rice protoplasts (Fig. 7)
OT38-4-R2	GCAGGATGTTTTCCCGGGTGGCGGA	
OT38-4-F3	GCCGCGAGCTCGCACCTTGCAGACTGT	2nd PCR for deep sequencing of OT38-4 for sgRNA 38 treated by Cas9-TV/sgRNA-dsgRNA in rice protoplasts (Fig. 7)
OT38-4-R3	GGCGGTTGTTTTCCCGGGTGGCGGA	
OT38-4-F4	GTATTAAGCTCGCACCTTGCAGACTGT	2nd PCR for deep sequencing of OT38-4 for sgRNA 38 in control rice protoplasts (Fig. 7)
OT38-4-R4	TACGTGTGTTTTCCCGGGTGGCGGA	
S26-DNase-F	TGGAACAGCCTCATCCCTTGGC	Analyze local chromatin accessibility change of Site 26
S26-DNase-R	CATGGTGGGAGTCTGTGGTGGT	
S28-DNase-F	GATACCAACCATGCATCGGCCA	Analyze local chromatin accessibility change of Site 28
S28-DNase-R	CTCTTGCTGCAGGGGCGACAA	
S34-DNase-F	CGACCCCAAGGCGTTCTGTTCCA	Analyze local chromatin accessibility change of Site 34
S34-DNase-R	CTCGTCCCCTTTCCACTGCTCG	
S2-Q-F	GCAACACCTTCAGGAGCAAC	Analyze gene expression using real-time PCR
S2-Q-R	GGTGTTCCCCTTGTCATCGATC	
S16-Q-F	GTTCGGGTTCGTGACGTTC	
S16-Q-R	CCTCGTTGACGGTGATGTTG	
S18-Q-F	GGGTGAAGGATCTGGATGCTG	
S18-Q-R	CTGAGGAAATTTGCTGTCAGCTC	
S26-Q-F	AGGCCTGAACAAAGCGATCT	
S26-Q-R	GTGCTTGACGATTGCCCTTG	
DSG-yuc-F	GGCATCGGCGTCGTTGG	Constructing vector expressing dsgRNA for *OsYUCCA4* promoter region
DSG-yuc-R	AAACCCAACGACGCCGA	

DSG-2-F	GGCAGACATCATCTGGCA	Constructing vector expressing dsgRNA for *LOC_Os04g31340* promoter region
DSG-2-R	AAACTGCCAGATGATGTC	
DSG-16-F	GGCAGGACCTTGGACTCG	Constructing vector expressing dsgRNA for *LOC_Os12g43600* promoter region
DSG-16-R	AAACCGAGTCCAAGGTCC	
DSG-18-F	GGCACCTGATTGGGTGA	Constructing vector expressing dsgRNA for *LOC_Os06g47340* promoter region
DSG-18-R	AAACTCACCCAATCAGG	
DSG-26-F	GGCACTCATCGGTGTGT	Constructing vector expressing dsgRNA for *LOC_Os04g11390* promoter region
DSG-26-R	AAACACACACCGATGAG	
YUCCA-Q-F	GTTGTCATCCTGGCCACAGGATAC	Analyze gene expression using real-time PCR
YUCCA-Q-R	CCAACAGCGTACAGGCCATTCTG	
Ubi-Q-F	GCTCCGTGGCGGTATCATC	
Ubi-Q-R	CGGCAGTTGACAGCCCTAG	

Sequence of the vectors used in this study

pOsU3-sgRNA

AAGGAATCTTTAAACATACGAACAGATCACTTAAAGTTCTTCTGAAGCAACTTAAAGTTATCAGGCATGCATGGATCTTGGAGGAATCAGATGTGCAGTCAGGGACCATA
GCACAAGACAGGCGTCTTCTACTGGTGCTACCAGCAAATGCTGGAAGCCGGGAACACTGGGTACGTCGGAAACCACGTGATGTGAAGAAGTAAGATAAACTGTAGGAGAA
AAGCATTTCGTAGTGGGCCATGAAGCCTTTCAGGACATGTATTGCAGTATGGGCCGGCCCATTACGCAATTGGACGACAACAAAGACTAGTATTAGTACCACCTCGGCTA
TCCACATAGATCAAAGCTGATTTAAAAGAGTTGTGCAGATGATCCGTGGCATGAGACCAACACAAGGTCTCGGTTTTAGAGCTAGAAATAGCAAGTTAAAATAAGGCTAG
TCCGTTATCAACTTGAAAAAGTGGCACCGAGTCGGTGCTTTTTTT

The rice U3 promoter and sgRNA scaffold are highlighted in green and red. The transcription initiation sites of rice U3 promoter is indicated in yellow. Two *Bsa* I sites are underlined. The guide sequence can be inserted between the two *Bsa* I sites using annealed oligos.

2TAL(flanked by *Avr* II and *Nhe* I)

CCTAGGGGCGGAAGCGGCGGCCTCCTGGACCCAGGAACCCCGATGGACGCCGATCTCGTGGCGTCCAGCACAGTGGTCTGGGAGCAGGATGCGGATCCATTCGCGGGAAC
CGCGGACGATTTCCCAGCCTTCAACGAGGAGGAGCTGGCCTGGCTGATGGAGCTCCTGCCACAGGGCGGCTCAGGCGGCCTCCTGGATCCTGGAACACCGATGGACGCGG
ATCTCGTGGCCTCATCTACCGTCGTCTGGGAGCAGGACGCCGATCCTTTCGCCGGCACAGCGGACGATTTCCCGGCCTTCAATGAGGAGGAGCTCGCGTGGCTGATGGAG
CTGCTCCCACAGGCTAGC

VP64(flanked by *Avr* II and *Nhe* I)

CCTAGGGGCTCAGACGCCCTGGATGATTTCGACCTCGATATGCTGGGCTCAGATGCCCTGGACGACTTTGACCTCGACATGCTCGGCTCTGACGCGCTCGACGACTTTGA
CCTCGATATGCTCGGTTCCGATGCCCTGGACGACTTCGACCTCGACATGCTCGCTAGC

Cas9-6TAL-VP128

ATGGCCCCTAAGAAGAAGAGAAAGGTCGGTATTCACGGCGTTCCTGCGGCGATGGACAAGAAGTATAGTATTGGTCTGGACATTGGGACGAATTCCGTTGGCTGGGCCGT

GATCACCGATGAGTACAAGGTCCCTTCCAAGAAGTTTAAGGTTCTGGGGAACACCGATCGGCACAGCATCAAGAAGAATCTCATTGGAGCCCTCCTGTTCGACTCAGGCG
AGACCGCCGAAGCAACAAGGCTCAAGAGAACCGCAAGGAGACGGTATACAAGAAGGAAGAATAGGATCTGCTACCTGCAGGAGATTTTCAGCAACGAAATGGCGAAGGTG
GACGATTCGTTCTTTCATAGATTGGAGGAGAGTTTCCTCGTCGAGGAAGATAAGAAGCACGAGAGGCATCCTATCTTTGGCAACATTGTCGACGAGGTTGCCTATCACGA
AAAGTACCCCACAATCTATCATCTGCGGAAGAAGCTTGTGGACTCGACTGATAAGGCGGACCTTAGATTGATCTACCTCGCTCTGGCACACATGATTAAGTTCAGGGGCC
ATTTTCTGATCGAGGGGGATCTTAACCCGGACAATAGCGATGTGGACAAGTTGTTCATCCAGCTCGTCCAAACCTACAATCAGCTCTTTGAGGAAAACCCAATTAATGCT
TCAGGCGTCGACGCCAAGGCGATCCTGTCTGCACGCCTTTCAAAGTCTCGCCGGCTTGAGAACTTGATCGCTCAACTCCCGGGCGAAAAGAAGAACGGCTTGTTCGGGAA
TCTCATTGCACTTTCGTTGGGGCTCACACCAAACTTCAAGAGTAATTTTGATCTCGCTGAGGACGCAAAGCTGCAGCTTTCCAAGGACACTTATGACGATGACCTGGATA
ACCTTTTGGCCCAAATCGGCGATCAGTACGCGGACTTGTTCCTCGCCGCGAAGAATTTGTCGGACGCGATCCTCCTGAGTGATATTCTCCGCGTGAACACCGAGATTACA
AAGGCCCCGCTCTCGGCGAGTATGATCAAGCGCTATGACGAGCACCATCAGGATCTGACCCTTTTGAAGGCTTTGGTCCGGCAGCAACTCCCAGAGAAGTACAAGGAAAT
CTTCTTTGATCAATCCAAGAACGGCTACGCTGGTTATATTGACGGCGGGGCATCGCAGGAGGAATTCTACAAGTTTATCAAGCCAATTCTGGAGAAGATGGATGGCACAG
AGGAACTCCTGGTGAAGCTCAATAGGGAGGACCTTTTGCGGAAGCAAAGAACTTTCGATAACGGCAGCATCCCTCACCAGATTCATCTCGGGGAGCTGCACGCCATCCTG
AGAAGGCAGGAAGACTTCTACCCCTTTCTTAAGGATAACCGGGAGAAGATCGAAAAGATTCTGACGTTCAGAATTCCGTACTATGTCGGACCACTCGCCCGGGGTAATTC
CAGATTTGCGTGGATGACCAGAAAGAGCGAGGAAACCATCACACCTTGGAACTTCGAGGAAGTGGTCGATAAGGGCGCTTCCGCACAGAGCTTCATTGAGCGCATGACAA
ATTTTGACAAGAACCTGCCTAATGAGAAGGTCCTTCCCAAGCATTCCCTCCTGTACGAGTATTTCACTGTTTATAACGAACTCACGAAGGTGAAGTATGTGACCGAGGGA
ATGCGCAAGCCCGCCTTCCTGAGCGGCGAGCAAAAGAAGGCGATCGTGGACCTTTTGTTTAAGACCAATCGGAAGGTCACAGTTAAGCAGCTCAAGGAGGACTACTTCAA
GAAGATTGAATGCTTCGATTCCGTTGAGATCAGCGGCGTGGAAGACAGGTTTAACGCGTCACTGGGGACTTACCACGATCTCCTGAAGATCATTAAGGATAAGGACTTCT
TGGACAACGAGGAAAATGAGGATATCCTCGAAGACATTGTCCTGACTCTTACGTTGTTTGAGGATAGGGAAATGATCGAGGAACGCTTGAAGACGTATGCCCATCTCTTC
GATGACAAGGTTATGAAGCAGCTCAAGAGAAGAAGATACACCGGATGGGGAAGGCTGTCCCGCAAGCTTATCAATGGCATTAGAGACAAGCAATCAGGGAAGACAATCCT
TGACTTTTTGAAGTCTGATGGCTTCGCGAACAGGAATTTTATGCAGCTGATTCACGATGACTCACTTACTTTCAAGGAGGATATCCAGAAGGCTCAAGTGTCGGGACAAG
GTGACAGTCTGCACGAGCATATCGCCAACCTTGCGGGATCTCCTGCAATCAAGAAGGGTATTCTGCAGACAGTCAAGGTTGTGGATGAGCTTGTGAAGGTCATGGGACGG
CATAAGCCCGAGAACATCGTTATTGAGATGGCCAGAGAAAATCAGACCACACAAAAGGGTCAGAAGAACTCGAGGGAGCGCATGAAGCGCATCGAGGAAGGCATTAAGGA
GCTGGGGAGTCAGATCCTTAAGGAGCACCCGGTGGAAAACACGCAGTTGCAAAATGAGAAGCTCTATCTGTACTATCTGCAAAATGGCAGGGATATGTATGTGGACCAGG
AGTTGGATATTAACCGCCTCTCGGATTACGACGTCGATCATATCGTTCCTCAGTCCTTCCTTAAGGATGACAGCATTGACAATAAGGTTCTCACCAGGTCCGACAAGAAC
CGCGGGAAGTCCGATAATGTGCCCAGCGAGGAAGTCGTTAAGAAGATGAAGAACTACTGGAGGCAACTTTTGAATGCCAAGTTGATCACACAGAGGAAGTTTGATAACCT
CACTAAGGCCGAGCGCGGAGGTCTCAGCGAACTGGACAAGGCGGGCTTCATTAAGCGGCAACTGGTTGAGACTAGACAGATCACGAAGCACGTGGCGCAGATTCTCGATT
CACGCATGAACACGAAGTACGATGAGAATGACAAGCTGATCCGGGAAGTGAAGGTCATCACCTTGAAGTCAAAGCTCGTTTCTGACTTCAGGAAGGATTTCCAATTTTAT

AAGGTGCGCGAGATCAACAATTATCACCATGCTCATGACGCATACCTCAACGCTGTGGTCGGAACAGCATTGATTAAGAAGTACCCGAAGCTCGAGTCCGAATTCGTGTA
CGGTGACTATAAGGTTTACGATGTGCGCAAGATGATCGCCAAGTCAGAGCAGGAAATTGGCAAGGCCACTGCGAAGTATTTCTTTTACTCTAACATTATGAATTTCTTTA
AGACTGAGATCACGCTGGCTAATGGCGAAATCCGGAAGAGACCACTTATTGAGACCAACGGCGAGACAGGGGAAATCGTGTGGGACAAGGGGAGGGATTTCGCCACAGTC
CGCAAGGTTCTCTCTATGCCTCAAGTGAATATTGTCAAGAAGACTGAAGTCCAGACGGGCGGGTTCTCAAAGGAATCTATTCTGCCCAAGCGGAACTCGGATAAGCTTAT
CGCCAGAAAGAAGGACTGGGACCCGAAGAAGTATGGAGGTTTCGACTCACCAACGGTGGCTTACTCTGTCCTGGTTGTGGCAAAGGTGGAGAAGGGAAAGTCAAAGAAGC
TCAAGTCTGTCAAGGAGCTCCTGGGTATCACCATTATGGAGAGGTCCAGCTTCGAAAAGAATCCGATCGATTTTCTCGAGGCGAAGGGATATAAGGAAGTGAAGAAGGAC
CTGATCATTAAGCTTCCAAAGTACAGTCTTTTCGAGTTGGAAAACGGCAGGAAGCGCATGTTGGCTTCCGCAGGAGAGCTCCAGAAGGGTAACGAGCTTGCTTTGCCGTC
CAAGTATGTGAACTTCCTCTATCTGGCATCCCACTACGAGAAGCTCAAGGGCAGCCCAGAGGATAACGAACAGAAGCAACTGTTTGTGGAGCAACACAAGCATTATCTTG
ACGAGATCATTGAACAGATTTCGGAGTTCAGTAAGCGCGTCATCCTCGCCGACGCGAATTTGGATAAGGTTCTCTCAGCCTACAACAAGCACCGGGACAAGCCTATCAGA
GAGCAGGCGGAAAATATCATTCATCTCTTCACCCTGACAAACCTTGGGGCTCCCGCTGCATTCAAGTATTTTGACACTACGATTGATCGGAAGAGATACACTTCTACGAA
GGAGGTGCTGGATGCAACCCTTATCCACCAATCGATTACTGGCCTCTACGAGACGCGGATCGACTTGAGTCAGCTCGGGGGGGATAAGAGACCAGCGGCAACCAAGAAGG
CAGGACAAGCGAAGAAGAAGAAGCCTAGGGGCGGAAGCGGCGGCCTCCTGGACCCAGGAACCCCGATGGACGCCGATCTCGTGGCGTCCAGCACAGTGGTCTGGGAGCAG
GATGCGGATCCATTCGCGGGAACCGCGGACGATTTCCCAGCCTTCAACGAGGAGGAGCTGGCCTGGCTGATGGAGCTCCTGCCACAGGGCGGCTCAGGCGGCCTCCTGGA
TCCTGGAACACCGATGGACGCGGATCTCGTGGCCTCATCTACCGTCGTCTGGGAGCAGGACGCCGATCCTTTCGCCGGCACAGCGGACGATTTCCCGGCCTTCAATGAGG
AGGAGCTCGCGTGGCTGATGGAGCTGCTCCCACAGGCTAGGGGCGGAAGCGGCGGCCTCCTGGACCCAGGAACCCCGATGGACGCCGATCTCGTGGCGTCCAGCACAGTG
GTCTGGGAGCAGGATGCGGATCCATTCGCGGGAACCGCGGACGATTTCCCAGCCTTCAACGAGGAGGAGCTGGCCTGGCTGATGGAGCTCCTGCCACAGGGCGGCTCAGG
CGGCCTCCTGGATCCTGGAACACCGATGGACGCGGATCTCGTGGCCTCATCTACCGTCGTCTGGGAGCAGGACGCCGATCCTTTCGCCGGCACAGCGGACGATTTCCCGG
CCTTCAATGAGGAGGAGCTCGCGTGGCTGATGGAGCTGCTCCCACAGGCTAGGGGCGGAAGCGGCGGCCTCCTGGACCCAGGAACCCCGATGGACGCCGATCTCGTGGCG
TCCAGCACAGTGGTCTGGGAGCAGGATGCGGATCCATTCGCGGGAACCGCGGACGATTTCCCAGCCTTCAACGAGGAGGAGCTGGCCTGGCTGATGGAGCTCCTGCCACA
GGGCGGCTCAGGCGGCCTCCTGGATCCTGGAACACCGATGGACGCGGATCTCGTGGCCTCATCTACCGTCGTCTGGGAGCAGGACGCCGATCCTTTCGCCGGCACAGCGG
ACGATTTCCCGGCCTTCAATGAGGAGGAGCTCGCGTGGCTGATGGAGCTGCTCCCACAGGCTAGGGGCTCAGACGCCCTGGATGATTTCGACCTCGATATGCTGGGCTCA
GATGCCCTGGACGACTTTGACCTCGACATGCTCGGCTCTGACGCGCTCGACGACTTTGACCTCGATATGCTCGGTTCCGATGCCCTGGACGACTTCGACCTCGACATGCT
CGCTAGGGGCTCAGACGCCCTGGATGATTTCGACCTCGATATGCTGGGCTCAGATGCCCTGGACGACTTTGACCTCGACATGCTCGGCTCTGACGCGCTCGACGACTTTG
ACCTCGATATGCTCGGTTCCGATGCCCTGGACGACTTCGACCTCGACATGCTCGCTAGGTAG

The NLS, Cas9, 6TAL and VP128 are highlighted in purple, blue, red and black, respectively. *Avr* II restriction site is underlined.
