# Supplementary material for: Modulating chromatin accessibility by transactivation and targeting proximal dsgRNAs enhances Cas9 editing efficiency in vivo
Source: Genome Biol. 2019 Jul 26;20:145. doi: 10.1186/s13059-019-1762-8 (PMC6660936; doi:10.1186/s13059-019-1762-8)
Supplement: Supplementary file 2 — Review history. (DOCX 19 kb) [file 13059_2019_1762_MOESM2_ESM.docx]

Review History

**Comments to author and authors’ point-by-point responses to the reviewers’ comments:**

**Reviewer 1**

This short article by Liu and colleagues describes the improvement of CRISPR-Cas9 cleavage at many target sites in the rice genome by fusion of multiple transcriptional activation domains to the Cas9 protein (Cas9-TV). While enhancement is seen at essentially all sequences, it brings modification of some targets in "closed" chromatin into a more useful range. Several other approaches to improving efficiency by influencing chromatin structure have appeared recently, and this one is a useful addition.

The effect of adding activation domains is, however, somewhat paradoxical. It has been shown both in biochemical experiments and in cells that the inhibitory effect of nucleosomes is on the binding of Cas9, not inherently on cleavage. Therefore, Cas9- TV must bind before its effect on chromatin structure can be manifested. The additional enhancement by including a non-cleaving Cas9-TV nearby has the same issue. It seems likely that there is a temporal window in both "open" and "closed" chromatin for binding, and once bound, Cas9-TV enforces more "open" chromatin in the vicinity. The authors should discuss this or other possible mechanisms directly.

*Response*: *We totally agree with this comment. As we mention in our ms “nucleosomes themselves are highly dynamic, and often undergo “site exposure” conformational fluctuations that transiently allow or block access of DNA binding proteins”. Chromatin structure is dynamic and relative in vivo. Thus, when Cas9-TV binds to a target in an “open” temporal window it will promote a more open chromatin state in the vicinity. We have added some more discussion of this matter in the revised ms (lines 219-228).*

In Figure 5, data for dsgRNA6 alone seem to be missing. Surprisingly, it seems to suppress the beneficial effect of dsgRNA3 when used in conjunction. It's hard to know what to make of the differences among the dsgRNAs, since their individual binding capabilities are not known.

*Response*: *We have performed the experiment again, this time including dsgRNA6 alone, and have replaced* *Figure 5 with the new data. We found that dsgRNA6 did further increase Cas9-TV-mediated editing; however, the editing efficiency using the dsgRNA4 and dsgRNA6 pair was lower than that using dsgRNA4 or dsgRNA6 alone. We have altered the text to “In addition, using pairs of dsgRNA rather than single dsgRNA did not further increase Cas9-TV-mediated editing, and sometimes even compromised the effect of a single dsgRNA (Fig. 5).”*

The authors should confess that several of the "closed" chromatin sites were not activated effectively (Figs. 3, 4), so there is still some way to go to produce a good universal tool.

*Response*: *We have added this point to the discussion (lines 254-256).*

Minor issues:

1.Line 103 - there is no editing in vitro; change "edit" to "cleave".

*Response*: *Done.*

2.Figure S6 is not cited in the text. What is it meant to show?

*Response*: *Figure S6 was used to show that proximal dsgRNA targeting also improves*

*Cas9 genome editing in both open and closed chromatin regions. We have now*

*included that data in Figure 4.*

3.Line 224 - Cpf1 should be changed to Cas12a.

*Response*: *Done.*

4.Which specific TALE protein did the TAL domains come from?

*Response*: *The TAL domain is from Xanthomonas campestris AvrBs3. We have included*

*this information in the revised ms (lines 114-116 and lines 269-270).*

**Reviewer 2**

The manuscript introduces the effect chromatin structure has on Cas9 editing and previous experiments that have increased efficiency including proxy- CRISPR and chromatin modulating peptides fused to Cas9. Using previous data and DNase I hypersensitivity, regions of open and closed chromatin were established and sgRNAs with sequences in both regions were compared. A fusion of TAL and VP16 domains were fused to Cas9 to create Cas9-TV which increased editing activity. This system was paired with dead sgRNAs (dsgRNA) to increase further accessibility.

Overall, the experiments are well designed, and the data are solid. The Cas9-TV construct may be very useful for improved genome editing in plants.

Major concern:

Line 207 states that the variability of Cas9-TV-dsgRNA editing suggests that chromatin openness changes in vivo. While this is supported by previous work the authors cited, including chromatin remodeling during human IPS cell reprogramming, variability in editing could be due to several factors. The authors should consider other reasons besides chromatin why differences were seen, especially when comparing Cas9-TV and Cas9 at open chromatin sites. If Cas9-TV is only affecting chromatin, then it would not be expected to outperform Cas9 at open chromatin. If the authors want to further explore changing chromatin in vivo they could consider parallels between IPS induction and the mutagenic process of rice tissue culture.

*Response*: *We quite agree that there are other factors than chromatin structure that affect genome-editing efficiency in vivo. The study of iPS undoubtedly suggests other methods for modulating chromatin structure, and we will explore them in future; we now consider this matter in the discussion (lines 253-255 and lines 225-228).*

There are two issues need to be addressed. First, the chromatin accessibility should be measured for four combinations in Fig. 6, including Cas9/sgRNA, Cas9-TV/sgRNA, Cas9/sgRNA-dsgRNA, and Cas9-TV/sgRNA-dsgRNA. Such data is critical to the conclusion on chromatin openness and genome editing activity. Based on the current Fig. 6, we cannot learn the degree of chromatin openness is contributed by Cas9-TV and sgRNA-dsgRNA respectively.

*Response*: *As the reviewer suggests, we have evaluated the effects of Cas9, Cas9-TV, Cas9/dsgRNA, and Cas9-TV/dsgRNA on chromatin accessibility in parallel. Fig. 6 is now updated with the results for these four combinations.*

Second, it is equally possible that improved NHEJ mutagenesis rate is due to increase target gene expression with the Cas9-TV constructs. All the data sets in this manuscript are more about correlation (rather than causal relationship). Did the authors look at target gene expression to test this alternative hypothesis (e.g. whether gene editing efficiency is positively correlated with gene expression/transcription activity)? This possibility needs to be discussed.

*Response*: *We agree that strong expression of genes facilitates genome editing, but this may not apply to the increased editing efficiencies of the target genes we tested since the sgRNAs we chose target the coding regions of the genes-of-interest. To confirm this, we performed real-time quantitative PCR to analyze the expression levels of the targeted genes, and found that introducing the Cas9-TV constructs did not alter the expression levels of these genes. We have included this result in the revised ms as the new Supplementary Fig. S8, and also discuss it in the text (lines 162-167).*

Minor points:

1.Line 116, please cite the Nature Plant paper describing the dCas9-TV work. It is better to give the paper credit here rather than later, as it will mislead readers that this is a new idea regarding TV fusion.

*Response*: *Thanks for the suggestion. We now cite this paper on line 114 of the main text.*

2.Line 168 states that potential off-target sites were identified. Authors should include how they identified these sites, whether through software or a manual search.

*Response*: *We identified off-target sites with the online tool CRISPR-P and we have included this information in both main text and Methods.*

3.The colors of the figures should be changed or cross-hatching should be added so that the figures are more readable in black and white. Figures that contain the DHS and Non-DHS should explain what the dotted lines and the shape of the graph mean.

*Response*: *We have changed the colors of the figures, and also explain the dotted line and the shape of the graph in the legends to Figure 1, Figure 3 and Figure 4.*

4.Figure 4 describes the effect of dead sgRNA targeting on indel frequency. Data from Cas9-dsgRNA indel frequency should be taken from supplementary and included in this figure for better comparison.

*Response*: *We have transferred the data for Cas9-dsgRNA indel frequencies from Figure S6 to Figure 4.*

5.Tissue culture and agro transformation methods are missing.

*Response*: *This has been added to Methods.*

6.While it is great that sgRNA-dsgRNA improved genome editing in plants, a similar strategy with multiplexing 2 or 3 sgRNAs for targeting the same gene is commonly practiced. In other words, the results from this study suggest multiplexing multiple sgRNAs in close vicinity on the same target gene may have synergist editing effects.

The authors should at least mention this in discussion so that users don't necessarily have to use a sgRNA-dsgRNA strategy.

*Response*: *We have added some discussion of this in the revised ms (lines 238-242).*

Overall, the manuscript was understandable with a few grammar errors at lines 139, 203, 206, 215, 247.

*Response*: *Thank you. We have corrected these grammatical errors.*
